# Fe(iii)-catalysed selective C–N bond cleavage of *N*-phenylamides by an electrochemical method†

**DOI:** 10.1039/d2ra04709h

**Published:** 2022-08-25

**Authors:** Yiwen Xu, Yang Long, Runyou Ye, Qiang Li, Fang Ke, Xiangge Zhou

**Affiliations:** College of Chemistry, Sichuan University Wangjiang Road 29 Chengdu 610064 China zhouxiangge@scu.edu.cn; School of Pharmacy, Fujian Provincial Key Laboratory of Natural Medicine Pharmacology, Fujian Medical University Fuzhou 350004 China kefang@mail.fjmu.edu.cn

## Abstract

An Fe(iii)-catalysed transformation of secondary *N*-phenyl substituted amides to primary amides by an electrochemical method is developed. Regioselective aryl C–H oxygenation occurs during the reaction, promoting selective C(phenyl)-N bond cleavage to form primary amides in yields of up to 92%.

## Abstract 2

An Fe(iii)-catalysed transformation of secondary *N*-phenyl substituted amides to primary amides by an electrochemical method is developed.

## Graphical abstract

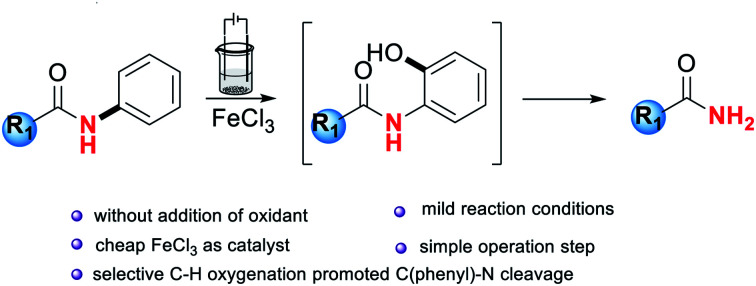

Amido groups are important structural units in organic compounds, which can be widely found in organic molecules, bioactive drugs, polymer functional materials *etc.*^1^ The research on modification or derivation of amides has flourished recently. For example, Szostak and co-workers reported C(O)–N bond activation of amides by nickel, palladium and rhodium catalysts, which could be followed by coupling reactions to form a series of carbonyl compounds including ketones, amides, esters and thioesters (Scheme 1a).^2^ Meanwhile, in the case of *N*-phenyl substituted amide substrates, which contain two C–N bonds in the same molecule, the selective C(phenyl)–N bond cleavage is scarcely reported compared with that of the C(O)–N bond.^3^ One of the possible reasons was reported to be the greater conjugation ability of n_N_ → π*_aryl_ than that of n_N_ → π*_C*=O*_, which might cause the bond energies of the C(phenyl)–N and C(O)–N bonds to be 104 and 95 kcal mol^−1^ respectively.^4^ The selective C(phenyl)–N bond cleavage is highly challenging and of interest to academic as well as industrial chemists. Zhang and co-workers unveiled an example of IBX-promoted selective cleavage of the C(phenyl)–N bond, and the usage of a stoichiometric oxidant during the reaction would limit its practical applications, which makes the selective cleavage of the C(phenyl)–N bond challenging (Scheme 1b).^5^

**Scheme 1:**
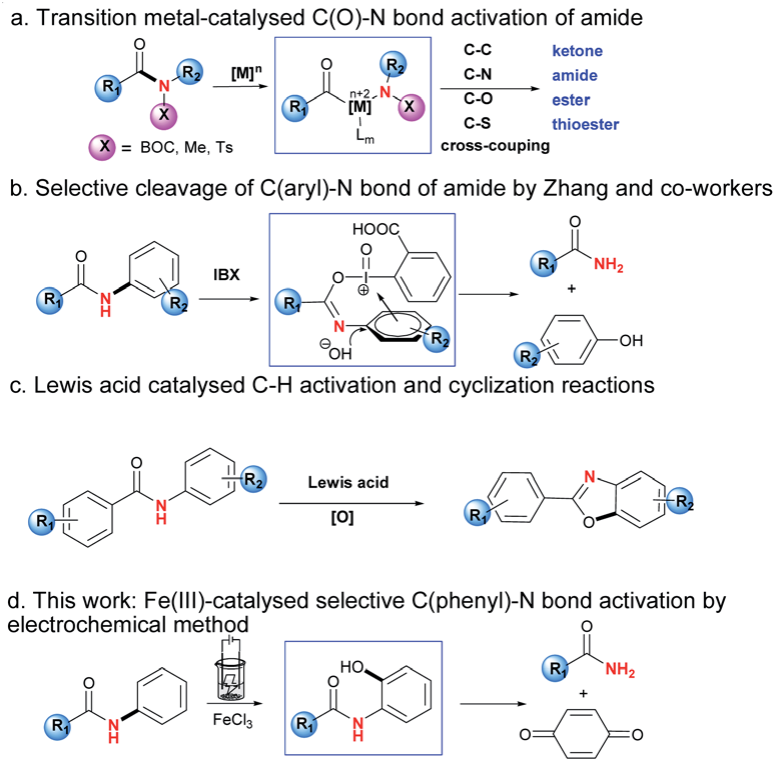
Strategies for activation of amido C–N bonds.

On the other hand, with the rapid revival of electrochemistry, which is regarded as an environmentally benign and powerful tool for a sustainable alternative to conventional redox approaches, impressive progress has been recently achieved for redox art through an electrochemistry.^6^ Especially, metal electrocatalytic reaction could serve as an attractive synthetic route to realize reactions that cannot be performed under conventional metal catalysis.^7^ In these reactions, metal catalyst could be coordinated with substrate to generate stable intermediate under constant current, affording highly value-added products.

Furthermore, Nagasawa and co-workers recently developed a Lewis acid/O_2_ catalytic system, which realised the synthesis of heterocycles by C–H functionalisation/C–O bond formation protocol (Scheme 1c).^8^ In these reactions, ground-state-stabilisation of amide was destroyed by strong affinitie of Lewis acid toward oxygen atom of amido group, lowering the resonance effects and thus electronic delocalisation.^9^

In continuation of our work on transition metal-catalysed inert bond activation and electrochemical work,^10^ herein is reported the selective C(phenyl)–N bond cleavage by the redox direct-oxidation of metal oxygenation catalysts at the electrode surface, which transformed secondary *N*-phenylamides to be primary amides (Scheme 1d). This protocol features the following items: cheap FeCl_3_ as catalyst; regioselective C–H oxygenation assisted chemoselective C(phenyl)–N bond cleavage of secondary phenylamide; avoidance of addition of oxidant; mild reaction conditions including room temperature and without inert atmosphere.

At the beginning of our exploration, *N*–phenylbenzamide (1a) was chosen as template substrate to identify optimal reaction conditions (for details, see Table S1 in ESI†). As shown in Table 1, the desired primary amide product 2a was obtained in 82% yield after 24 h under 20 mA with Fe plate as anode and Pt net as cathode, together with ^*n*^Bu_4_NPF_6_ (0.05 M) as electrolyte in mixture solvent of MeCN (3 mL) and H_2_O (3 mL) at room temperature in the presence of FeCl_3_·6H_2_O (0.2 equiv.) as catalyst (Table 1, entry 1). Control experiments showed that electricity and supporting electrolyte was essential for the reaction, and ^*n*^Bu_4_NPF_6_ was found to be more fitful for the reaction than other phase transfer reagents including ^*n*^Bu_4_NBr and ^*n*^Bu_4_NClO_4_ (Table 1, entries 2–4). After screening of different transition metal salts, FeCl_3_·6H_2_O was proved to be proper choice compared with ZnCl_2_, Cu(OTf)_2_, Fe(OAc)_2_ and Fe(acac)_3_ (Table 1, entries 5–8). Meanwhile, solvent seemed to be an important factor to affect the results, and the mixture solvent of MeCN and H_2_O in a ratio of 1 : 1 was the best one. Attempts to replace H_2_O by other protic solvents resulted in inferior yields (Table 1, entries 9–14). Subsequently, the influence of reaction time was tested, and longer reaction time than 24 h seemed to be unfavourable for the reaction, which might be caused by the hydrolysis of product 2a to be benzoic acid monitored by GC/Mass (Table 1, entries 15 and 16). The electrode material sacrificial anode also had an important impact on the reaction (Table 1 entry 17 and 18). At last, it was reported the addition of DABCO (1,4-diaza-bicyclo[2.2.2]octane) as sacrificial reductant would prevent the hydrolysis of product,^11^ however, it seemed to be unnecessary in this reaction, which would result in inferior yield 30% (Table 1, entry 19).

**Table 1:** Optimisation of reaction conditions a

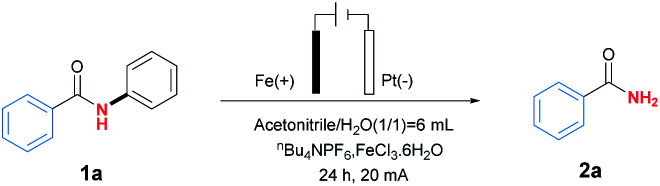		
Entry	Changes from standard conditions	Yield (%)^b^
1	None	89/82^c^
2	Without electricity	N.D
3	^*n*^Bu_4_NBr instead of ^*n*^Bu_4_NPF_6_	10
4	^*n*^Bu_4_NClO_4_ instead of ^*n*^Bu_4_NPF_6_	18
5	ZnCl_2_ instead of FeCl_3_·6H_2_O	58
6	Cu(OTf)_2_ instead of FeCl_3_·6H_2_O	40
7	Fe(OAc)_2_ instead of FeCl_3_·6H_2_O	44
8	Fe(acac)_3_ instead of FeCl_3_·6H_2_O	63
9	Without MeCN	Trace
10	Without H_2_O	N.D.
11	Ethylene carbonate instead of MeCN	18
12	DMF instead of MeCN	11
13	Methanol instead of H_2_O	32
14	Acetic acid instead of H_2_O	Trace
15	14 h instead of 24 h	24
16	36 h instead of 24 h	62
17	Pt(+) instead of Fe(+)	60
18	Ni(+) instead of Fe(+)	87
19	DABCO as additives	30

a Standard conditions: 1a (0.6 mmol), H_2_O (3 mL), MeCN (3 mL), ^*n*^Bu_4_NPF_6_ (0.05 M), FeCl_3_·6H_2_O (0.2 equiv.), Pt net and Fe plate electrodes (1.0 × 1.0 cm^2^), 20 mA, undivided cell, room temperature, air, 24 h.

b Yields were determined using GC analysis with 1H-benzo[*d*]imidazole as internal standard.

c Isolated yield.

With the optimised reaction conditions in hand, the substrate scope was then explored as shown in Table 2. In general, this transformation was amenable to substrates containing different substituents, and a series of *N*-phenylbenzamides efficiently underwent C(aryl)–N bond cleavage dephenylation reactions to afford primary amides 2. Steric hindrance played an important role in the reaction. For example, the *ortho* and *para*-chloro substituted substrates gave the corresponding products 2e and 2o in yields 46% and 63% respectively. Other halogen substituents including Br and I could also be applied in this reaction and delivered the desired products 2d, 2f and 2i in moderate yields ranging from 40%–70%, providing the potentiality for late-stage functionalisation when combing with traditional cross-coupling strategies. Meanwhile, electron-rich substituents seemed to be beneficial to the reaction, and the best yield 92% was obtained in the case of product 2n containing *para*-methoxyl substituent. Furthermore, substrates bearing heterocyclic or naphthalene moiety could also be tolerated in this transformation to give the corresponding products, including niacinamide 2w in yield of 79%. More importantly, the aliphatic amides were also applied in this reaction, affording the desired products 2aa-2hh in yields ranging from 23% to 84%. The stable rigid structure of adamantane could undergo this dephenylation reaction to give 66% yields (2ii). Further studies revealed that chirality could be retained under the reaction conditions (2jj).

**Table 2:** Substrate scope for the electroiron-catalysed selective C–N bond activation of *N*-phenyl amides.^a^^,^^b^^,^^c^

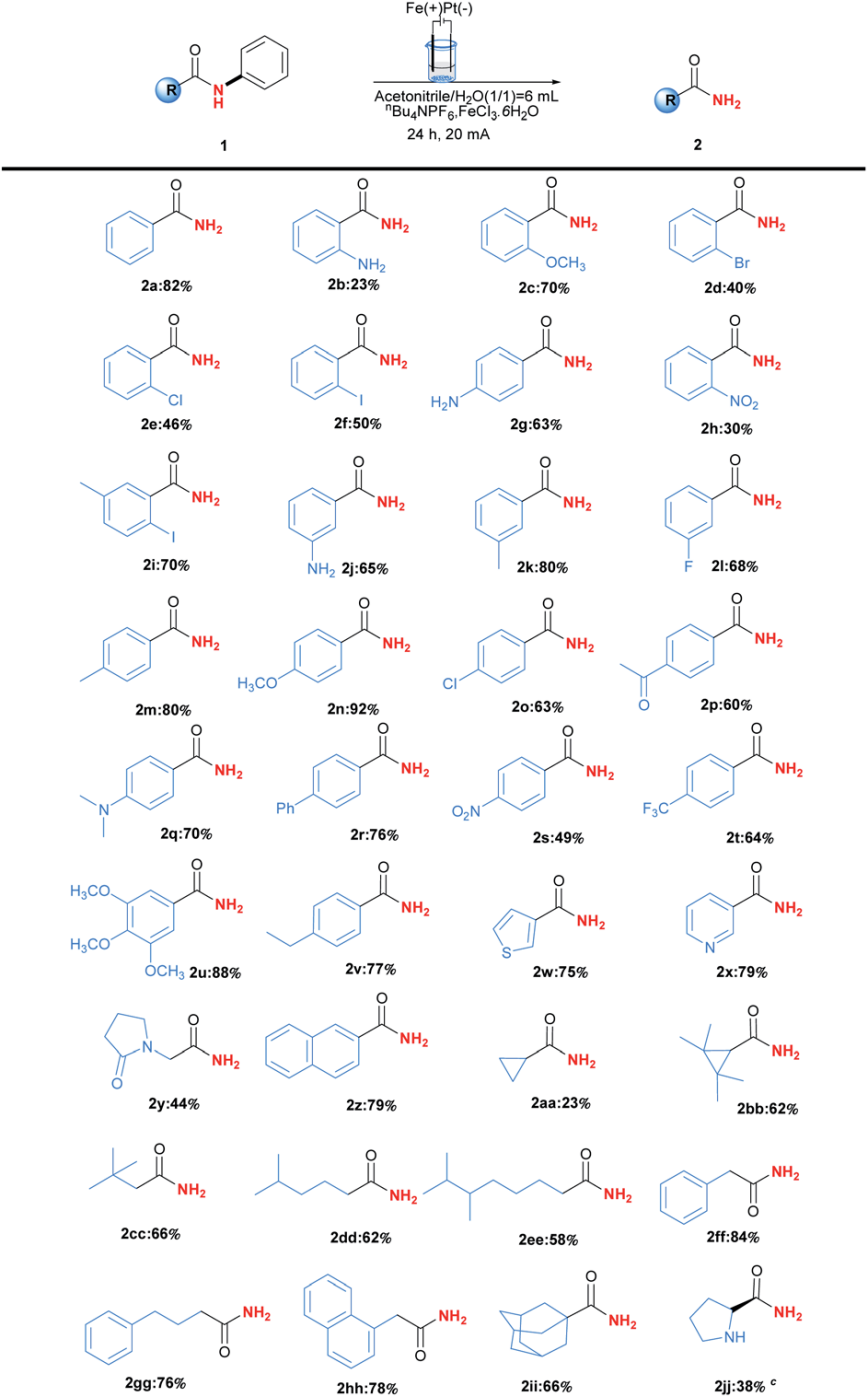

a Standard conditions: 1a (0.6 mmol), H_2_O (3 mL), MeCN (3 mL), ^*n*^Bu_4_NPF_6_ (0.05 M), FeCl_3_·6H_2_O (0.2 equiv.), Pt net and Fe plate electrodes (1.0 × 1.0 cm^2^), 20 mA, undivided cell, room temperature, air, 24 h.

b Yields were determined using GC analysis with 1*H*-benzo[*d*]imidazole as internal standard.

c Isolated yield.

To clarify the possible reaction pathway, a series of experiments were then carried out as shown in Scheme 2. At first, *N*-(2-hydroxyphenyl)benzamide (3a) and *p*-benzoquinone (4a) were detected during the reaction, and decrement of reaction time from 24 hours to 14 hours resulted in the formation of 2a, 3a and 4a in yields 34%, 16% and 30%, which indicated 3a would be intermediate during reaction (Scheme 2a). Further experiment by using 3a as substrate also afforded the corresponding product 2a smoothly (Scheme 2b). Meanwhile, when *ortho* or *meta* position of *N*-phenyl group was occupied by substituents, no product could be formed, which suggested the necessity of vacancy of *ortho* and meta position for C–H oxygenation process (Scheme 2c).

**Scheme 2:**
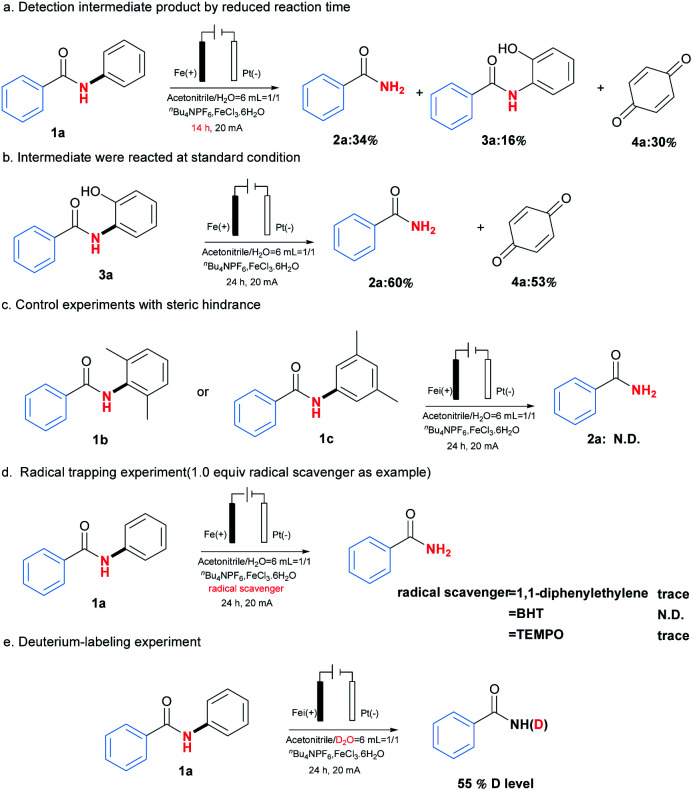
Control experiments for reaction pathway studies.

Next, the addition of radical scavengers such as 1,1-diphenylethylene, butylated hydroxytoluene (BHT) or 2,2,6,6-tetramethylpiperidine-1-oxyl (TEMPO) would completely inhibit the reaction, suggesting the reaction might involve a radical pathway (Scheme 2d).^12^ At last, when D_2_O was used instead of H_2_O as solvent, 55% of deuterium incorporation was observed, which demonstrated the reaction would involve a hydrolysis process (Scheme 2e).

Meanwhile, the cyclic voltammetric (CV) experiments were carried out as shown in Fig. 1. Curves a, b and c exhibited redox peaks for blank, Fe(ii) and Fe(iii), which indicated that Fe(i) would not be involved in this reaction. Meanwhile, curve b showed Fe(ii)_i*pa*_ at −0.49 mA, Fe(ii)_i*pc*_ at 0.15 mA and Fe(iii)_i*pa*_ at −0.10 mA, while curve c showed Fe(ii)_i*pa*_ at −0.30 mA, Fe(ii)_i*pc*_ at 0.07 mA and Fe(iii)_i*pa*_ at −0.30 mA, which indicated Fe(ii) would participate in the reaction to form product from intermediate 3a.

**Fig. 1:**
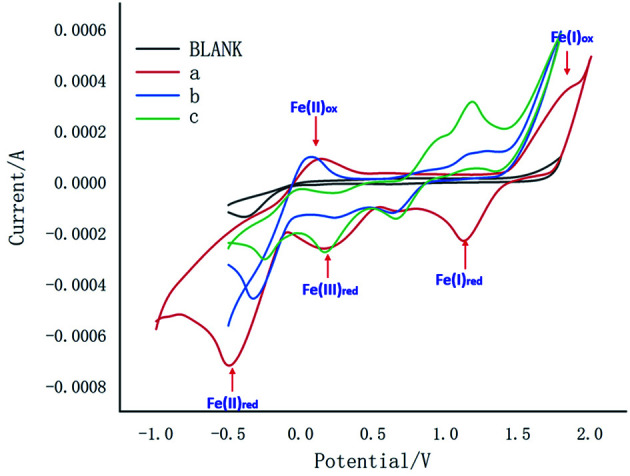
Cyclic voltammograms experiment (Blank: 0.1 M ^*n*^Bu_4_NPF_6_, MeCN/H_2_O = 1/1; a: Blank + FeCl_3_·6H_2_O 0.02 mmol; b: Blank + 1a 0.1 mmol + FeCl_3_·6H_2_O 0.02 mmo; c: Blank + 3a 0.1 mmol + FeCl_3_·6H_2_O 0.02 mmol).

Based on the above experimental results and literature survey,^13^ a plausible reaction pathway for Fe(iii)-catalysed selective C–N bond activation of *N*-phenyl amides promoted by regioselective C–H oxygenation was proposed (Fig. 2). At first, coordination of Fe(iii) with 1a^14^ would activate amide carbonyl to lose electron efficiently at anode, forming free radical A.^15^ Meanwhile, solvent H_2_O would be oxidised to be radical ˙OH in the reaction. Next, the radical coupling reaction between A and ˙OH would form intermediate 3a.^16^ Then, 3a would be reacted with iron salt to form complex B,^9(d)^ which was further transformed to be intermediates C and D after a single electron transfer (SET) process and a radical reaction with ˙OH.^17^ At last, reduction of D resulted in the generation of intermediate E at cathode, which was finally converted to be product 2a by a hydrogen reduction process.^18^

**Fig. 2:**
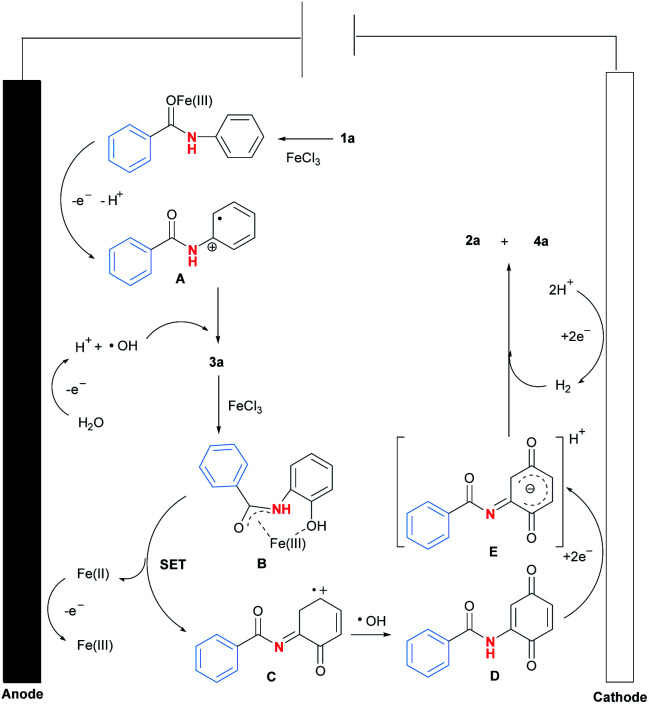
Possible reaction pathway.

The applications of this transformation were then carried out. As shown in Scheme 3a, telmisartan derivative could be easily transformed to be free amide 2kk in 42% yield, which could broaden *N*-phenylamides' further applications. Moreover, this methodology could be applied for the synthesis of trimethobenzamide 5,^19^ a powerful antihistamine, starting from 3,4,5-trimethoxybenzamide (2u) (Scheme 3b). These results indicated this transformation expanded the type of synthetic methodologies with phenyl groups as removable protecting groups under mild reaction conditions. At last, the gram-scale synthesis of 1a (6 mmol) with 60 mA under the optimal reaction conditions was performed, furnishing the desired product 2a in 72% yield (Scheme 4).

**Scheme 3:**
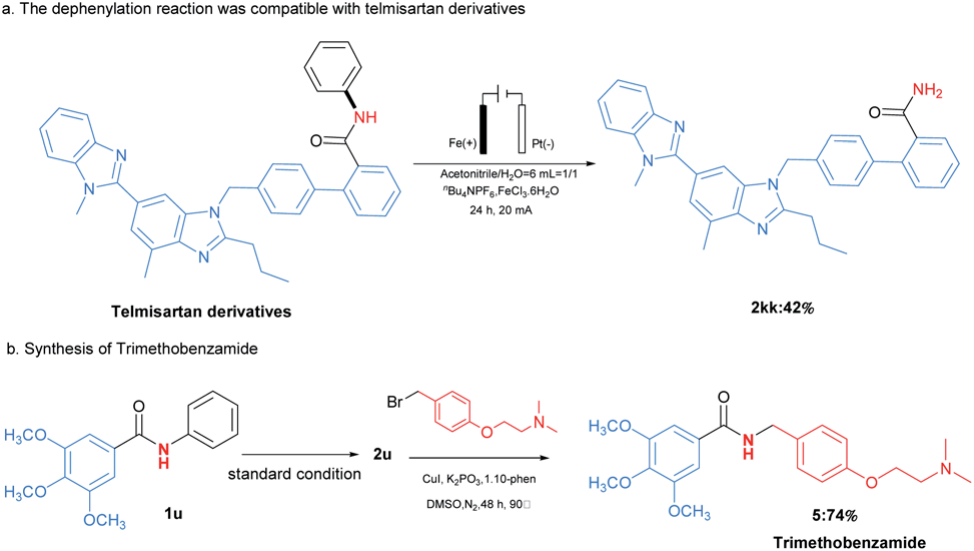
Applications in the synthesis of selected compounds.

**Scheme 4:**
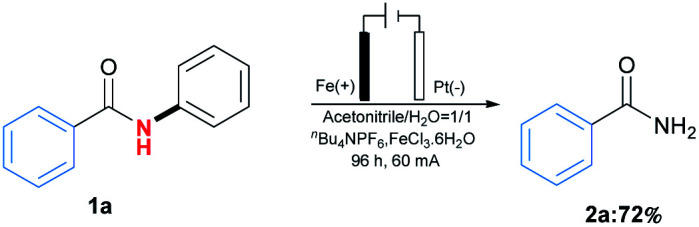
Gram-scale experiment.

In summary, we have developed an iron-catalysed, electrochemical oxidation mediated strategy for selective C–N activation dephenylation of secondary *N*-phenylamide. This transformation exhibits an ample scope, high yield, and broad functional group tolerance. A more distinct feature of this work pertains to its applicability to selective dephenylation of *N*-phenylamides without pre-activated amides. The development of new reactions based on this strategy is currently in progress.

## Conflicts of interest

There are no conflicts to declare.

## Supplementary Material

RA-012-D2RA04709H-s001
