# Supplementary material for: Fe(iii)-catalysed selective C–N bond cleavage of N-phenylamides by an electrochemical method
Source: RSC Adv. 2022 Aug 25;12(37):24217–21. doi: 10.1039/d2ra04709h (PMC9403817; doi:10.1039/d2ra04709h)

Electronic Supplementary Material (ESI) for RSC Advances.
This journal is © The Royal Society of Chemistry 2022

Fe(III) – catalysed selective C–N bond cleavage of *N*–phenylamide by electrochemical method

Yiwen Xu,[a] Yang Long,[a] Runyou Ye,[a] Qiang Li,[a] Fang Ke[b] and Xiangge Zhou*[a]

[a] College of Chemistry, Sichuan University, Chengdu 610064, P. R. China, Fax: +86-28-85412907; Tel: +86-28-85412907; E-mail: zhouxiangge@scu.edu.cn

[b] School of Pharmacy, Fujian Provincial Key Laboratory of Natural Medicine Pharmacology, Fujian Medical University, Fuzhou 350004, P. R. China, Tel: +86-591-22862016; Fax: +86-591-22862016; E-mail: kefang@mail.fjmu.edu.cn

Supporting information

General information........3

Table S1: Optimisation of electrochemical dephenylation of secondary phenylamide [a]........3

Scheme S1: General procedure for the catalytic reactions........4

The setup for constant current electrolysis........5

Scheme S2: General procedure for gram scale reaction........5

Mechanism Studies........5

Scheme S3: Stepwise dephenylation to get intermediate product:........5

Scheme S4: Steric hindrance effect:........7

Table S2: Radical scavenger experiments[a]:........7

Cyclic voltammetry experiment........8

Scheme S5: Deuterium-labeling experiment........8

Scheme S6: Procedure for synthesis of Trimethobenzamide........10

Scheme S7: H_2O^{18} experiment........11

Experimental procedures and characterisation data........12

References........18

^{1}H NMR and ^{13}C NMR spectra for the products........19

General information

All reagents were purchased from commercial suppliers and used without further purification. Column chromatography was carried out with silica gel (100–200 mesh). Thin layer chromatography was carried out using Merck silica gel GF254 plates. All products were characterised by NMR. ^{1}H NMR spectra were recorded at 400 MHz and ^{13}C NMR spectra were recorded at 101 MHz (Bruker DPX) with $CDCl_3$ and DMSO–d_6 as solvent. Chemical shifts are reported in ppm using TMS as internal standard. Gas chromatography–mass spectra (GC/Mass) were recorded on an Agilent Technologies 6890 N instrument with an Agilent 5973N mass detector (EI) and a HP5–MS 30 cm*0.25 mm capillary apolar column (Stationary phase: 5% diphenyldimethylpolysiloxane film, 0.25 µm). GC/Mass method: Initial temperature: 50 °C; Initial time: 3 min; Ramp: about 20°C/min until 280 °C then 10 min. High resolution mass spectra (HR-MS) were obtained with a Shimadzu LCMS–IT–TOF (ESI). Shanghai chenhua CHI600E electrochemical workstation was used in the standard configuration as delivered, including proprietary software. The setup for constant current electrolysis(Shanghai xirui DJS–292B constant potentiometer) consisted of an undivided cell equipped with anode and cathode(Electrodes come from commercial suppliers), if not noted otherwise.

Table S1: Optimisation of electrochemical dephenylation of secondary phenylamide [a]

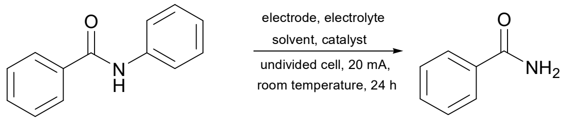

1a 2b

Enry	Solvents	Catalyst	Time	Temperature	Electrolyts	Electrode	Additives	Yields[b]
1	MeCN/H_2O	$FeCl_3·6H_2O$	24 h	room temperature(RT)	$^{n}Bu_4NBr$	Pt(+)Pt(-)	-	6
2	MeCN/H_2O	$FeCl_3·6H_2O$	24 h	RT	$^{n}Bu_4NClO_4$	Pt(+)Pt(-)	-	18
3	MeCN/H_2O	$FeCl_3·6H_2O$	24 h	RT	$^{n}Bu_4NF_3O_3S$	Pt(+)Pt(-)	-	7
4	MeCN/H_2O	$FeCl_3·6H_2O$	24 h	RT	$^{n}Bu_4NB$	Pt(+)Pt(-)	-	5
**5**	**MeCN/H_2O**	**$FeCl_3·6H_2O$**	**24 h**	**RT**	**$^{n}Bu_4NPF_6$**	**Pt(+)Pt(-)**	**-**	**60**
6	MeCN/H_2O	$FeCl_3·6H_2O$	24 h	RT	$^{n}Bu_4NBF_4$	Pt(+)Pt(-)	-	Trace
7	MeCN/H_2O	$FeCl_3·6H_2O$	24 h	RT	$^{n}Bu_4NI$	Pt(+)Pt(-)	-	19
8	MeCN/H_2O	$FeCl_3·6H_2O$	24 h	RT	$^{n}Bu_4NF.3H_2O$	Pt(+)Pt(-)	-	40
9	MeCN/H_2O	$FeCl_3·6H_2O$	24 h	RT	$^{n}Me_4NBr$	Pt(+)Pt(-)	-	Trace
10	MeCN/H_2O	$FeCl_3·6H_2O$	24 h	RT	$^{n}Bu_4NBr_3$	Pt(+)Pt(-)	-	Trace
11	MeCN/H_2O	$FeCl_3·6H_2O$	24 h	RT	$^{n}Pro_4NBr$	Pt(+)Pt(-)	-	Trace
12	MeCN/H_2O	$FeCl_3·6H_2O$	24 h	RT	$^{n}Et_4NI$	Pt(+)Pt(-)	-	26
13	MeCN/H_2O	$FeCl_3·6H_2O$	24 h	RT	$^{n}Bu_4NBH_4$	Pt(+)Pt(-)	-	Trace
14	MeCN/H_2O	$FeCl_3·6H_2O$	24 h	RT	-	Pt(+)Pt(-)	-	N.D.
15	MeCN/H_2O	$AlCl_3$·	24 h	RT	$^{n}Bu_4NPF_6$	Pt(+)Pt(-)	-	30
16	MeCN/H_2O	$ZnCl_2$	24 h	RT	$^{n}Bu_4NPF_6$	Pt(+)Pt(-)	-	58
17	MeCN/H_2O	$Zn(OTf)_2$	24 h	RT	$^{n}Bu_4NPF_6$	Pt(+)Pt(-)	-	Trace
18	MeCN/H_2O	$SbCl_3$	24 h	RT	$^{n}Bu_4NPF_6$	Pt(+)Pt(-)	-	Trace
19	MeCN/H_2O	$InCl_3$	24 h	RT	$^{n}Bu_4NPF_6$	Pt(+)Pt(-)	-	Trace
20	MeCN/H_2O	$Cu(OTf)_2$	24 h	RT	$^{n}Bu_4NPF_6$	Pt(+)Pt(-)	-	40
21	MeCN/H_2O	$In(OTf)_3$	24 h	RT	$^{n}Bu_4NPF_6$	Pt(+)Pt(-)	-	Trace
22	MeCN/H_2O	$Ni(OTf)_2$	24 h	RT	$^{n}Bu_4NPF_6$	Pt(+)Pt(-)	-	8
23	MeCN/H_2O	$RuCl_3$	24 h	RT	$^{n}Bu_4NPF_6$	Pt(+)Pt(-)	-	42
24	MeCN/H_2O	AgOTf	24 h	RT	$^{n}Bu_4NPF_6$	Pt(+)Pt(-)	-	30
25	MeCN/H_2O	Ag_2CO_3	24 h	RT	$^{n}Bu_4NPF_6$	Pt(+)Pt(-)	-	Trace
26	MeCN/H_2O	$FeAc_2$	24 h	RT	$^{n}Bu_4NPF_6$	Pt(+)Pt(-)	-	44
27	MeCN/H_2O	$Fe(acac)_3$	24 h	RT	$^{n}Bu_4NPF_6$	Pt(+)Pt(-)	-	63
28	MeCN/H_2O	$FeCp_2$	24 h	RT	$^{n}Bu_4NPF_6$	Pt(+)Pt(-)	-	12
29	MeCN/H_2O	$FeSO_4$	24 h	RT	$^{n}Bu_4NPF_6$	Pt(+)Pt(-)	-	44
30	MeCN/H_2O	$FeCl_2$	24 h	RT	$^{n}Bu_4NPF_6$	Pt(+)Pt(-)	-	53
31	MeCN/H_2O	D-i-prpf	24 h	RT	$^{n}Bu_4NPF_6$	Pt(+)Pt(-)	-	30
32	MeCN/H_2O	-	24 h	RT	$^{n}Bu_4NPF_6$	Pt(+)Pt(-)	-	N.D
33[c]	MeCN	$FeCl_3$	24 h	RT	$^{n}Bu_4NPF_6$	Pt(+)Pt(-)	-	N.D
34	H_2O	$FeCl_3·6H_2O$	24 h	RT	$^{n}Bu_4NPF_6$	Pt(+)Pt(-)	-	Trace
35	DMF/H_2O	$FeCl_3·6H_2O$	24 h	RT	$^{n}Bu_4NPF_6$	Pt(+)Pt(-)	-	11
36	MeCN	$FeCl_3·6H_2O$	24 h	RT	$^{n}Bu_4NPF_6$	Pt(+)Pt(-)	-	Trace
37	acetone /H_2O	$FeCl_3·6H_2O$	24 h	RT	$^{n}Bu_4NPF_6$	Pt(+)Pt(-)	-	8

38	ethylene carbonate /H_2O	$FeCl_3·6H_2O$	24 h	RT	$^{n}Bu_4NPF_6$	Pt(+)Pt(-)	-	18
39	CH_2Cl_2 /H_2O	$FeCl_3·6H_2O$	24 h	RT	$^{n}Bu_4NPF_6$	Pt(+)Pt(-)	-	12
40	MeCN/HFiP	$FeCl_3·6H_2O$	24 h	RT	$^{n}Bu_4NPF_6$	Pt(+)Pt(-)	-	N.D
41	MeCN/Formic acid	$FeCl_3·6H_2O$	24 h	RT	$^{n}Bu_4NPF_6$	Pt(+)Pt(-)	-	20
42	MeCN/HOAc	$FeCl_3·6H_2O$	24 h	RT	$^{n}Bu_4NPF_6$	Pt(+)Pt(-)	-	Trace
43	MeCN/^{t}BuOH	$FeCl_3·6H_2O$	24 h	RT	$^{n}Bu_4NPF_6$	Pt(+)Pt(-)	-	Trace
44	MeCN/EtOH	$FeCl_3·6H_2O$	24 h	RT	$^{n}Bu_4NPF_6$	Pt(+)Pt(-)	-	Trace
45	MeCN/MeOH	$FeCl_3·6H_2O$	24 h	RT	$^{n}Bu_4NPF_6$	Pt(+)Pt(-)	-	32
46	MeCN/H_2O	$FeCl_3·6H_2O$	24 h	RT	$^{n}Bu_4NPF_6$	Pt(+)Pt(-)	TMEDA^{f}	Trace
47	MeCN/H_2O	$FeCl_3·6H_2O$	24 h	RT	$^{n}Bu_4NPF_6$	Pt(+)Pt(-)	TFA·Et_3N^{d}	Trace
48	MeCN/H_2O	$FeCl_3·6H_2O$	24 h	RT	$^{n}Bu_4NPF_6$	Pt(+)Pt(-)	Et_3N^{e}	57
49	MeCN/H_2O	$FeCl_3·6H_2O$	24 h	RT	$^{n}Bu_4NPF_6$	Pt(+)Pt(-)	DABCO^{f}	30
**50**	**MeCN/H_2O**	**$FeCl_3·6H_2O$**	**24 h**	**RT**	**$^{n}Bu_4NPF_6$**	**Fe(+)Pt(-)**	**-**	**89**
**51**	**MeCN/H_2O**	**$FeCl_3·6H_2O$**	**24 h**	**RT**	**$^{n}Bu_4NPF_6$**	**Ni(+)Pt(-)**	**-**	**87**
52	MeCN/H_2O	$FeCl_3·6H_2O$	24 h	RT	$^{n}Bu_4NPF_6$	Al(+)Pt(-)	-	20
53	MeCN/H_2O	$FeCl_3·6H_2O$	24 h	RT	$^{n}Bu_4NPF_6$	Cu(+)Pt(-)	-	79
54	MeCN/H_2O	$FeCl_3·6H_2O$	14 h	RT	$^{n}Bu_4NPF_6$	Fe(+)Pt(-)	-	24
55	MeCN/H_2O	$FeCl_3·6H_2O$	36 h	RT	$^{n}Bu_4NPF_6$	Fe(+)Pt(-)	-	62
56	MeCN/H_2O	$FeCl_3·6H_2O$	48 h	RT	$^{n}Bu_4NPF_6$	Fe(+)Pt(-)	-	58
57	MeCN/H_2O	$FeCl_3·6H_2O$	24 h	35	$^{n}Bu_4NPF_6$	Fe(+)Pt(-)	-	88
58	MeCN/H_2O	$FeCl_3·6H_2O$	24 h	45	$^{n}Bu_4NPF_6$	Fe(+)Pt(-)	-	86
59	MeCN/H_2O	$FeCl_3·6H_2O$	24 h	60	$^{n}Bu_4NPF_6$	Fe(+)Pt(-)	-	87

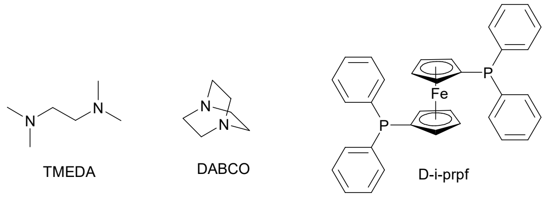

^{a} Standard conditions: Anode (1.0 *1.0 cm^2), Cathode (1.0 *1.0 cm^2), **1a** (0.6 mmol), Solvents (6 mL=1/1), Electrolytes (0.05 M), Catalyst (0.2 equiv), 20 mA, Undivided cell, air, 24 h. ^{b} Yields were determined using GC-Mass analysis with 1H-benzo[*d*]imidazole as an internal standard. ^{c} water removal; ^{d} 2M:2M solution (0.5 mL); ^{e} 0.5 mL; ^{f} 1.0 equiv.

Initially, various electrolytes were screened with water and MeCN as solvent and $FeCl_3·6H_2O$ as catalyst (Entries 1–12). $^{n}Bu_4NPF_6$ proved to be the optimal electrolyte giving product in 60% yield (Entry 5). Next, a series of Lewis acid and iron catalyst were tested (Entries 15–32). The results showed that $FeCl_3·6H_2O$ exhibited the highest catalytic activity than other catalyst. Water and MeCN were found to be the most suitable solvents for this system (Entries 32–45). H_2O plays an important role in this reaction system that cannot proceed for the reaction when removal (Entry 33). Electronic transfer media contribute little to the conversion under constant current (Entries 46–49). Further exploration of electrode (Entries 50-53) showed that Fe(+) Pt(-) were the optimal condition with 89% yield. Finally, the time and temperature screening revealed that 24 h and room temperature were the best choice (Entries 54–59).

Scheme S1: General procedure for the catalytic reactions

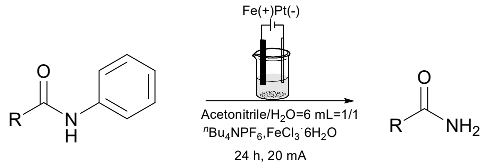

An undivided cell was equipped with a magnet stirrer, Fe plate (1.0 *1.0 cm^2), Pt net (1.0*10 cm^2), as anode electrode and cathode electrode. The substrate secondary phenylamide (0.6 mmol), $^{n}Bu_4NPF_6$ (0.05 M), $FeCl_3·6H_2O$ (20 %) , 3.0 mL of water and 3.0 mL MeCN were placed. The resulting mixture was allowed to stir and electrolyze at constant current conditions (20 mA) at room temperature for 24 hours. Then the reaction mixture was poured into water (10 mL) and extracted with ethyl acetate (10 mL*3). The residue was purified by silica gel column chromatography to obtain the corresponding product. All products were confirmed by nuclear magnetic resonance and mass spectrometry.

The setup for constant current electrolysis

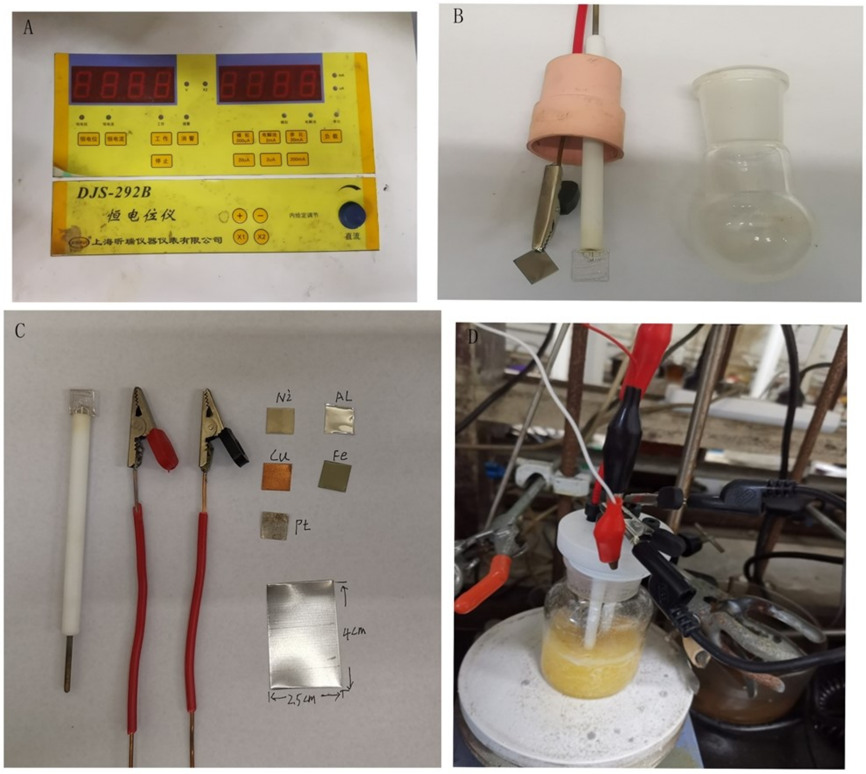

Figure S1. Electrolysis setup (A: Shanghai xirui DJS–292B constant potentiometer; B: Fe plate (1.0 *1.0 cm2), Pt net (1.0*10 cm2), An undivided cell of 10 mL flask; C: Series of electrodes; D: Gram scale reaction device).

Scheme S2: General procedure for gram scale reaction

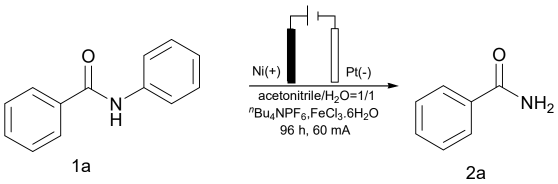

An undivided cell was equipped with a magnet stirrer, Fe plate (25*4.0 cm^2), two Pt net (10*10 cm^2), as the working electrode and counter electrode. The substrate secondary phenylamide (6.0 mmol), $^nBu_4NPF_6$ (0.05 M), $FeCl_3{\cdot}6H_2O$ (20 %) , 20.0 mL of water and 20.0 mL MeCN were placed. The reaction mixture was stirred at a constant current of 60 mA under room temperature for 96 h. Then the reaction mixture was poured into water (150 mL) and extracted with ethyl acetate (100 mL*3). The residue was purified by silica gel column chromatography in a yield around 72%.

Mechanism Studies

Scheme S3: Stepwise dephenylation to get intermediate product:

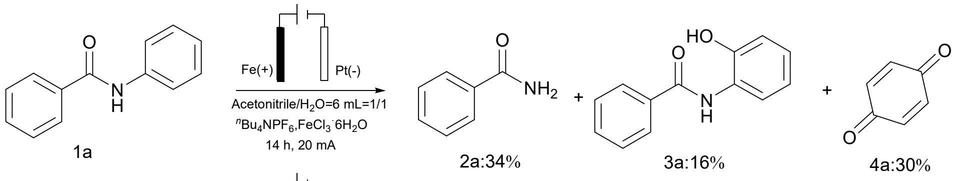

Eq(S1)

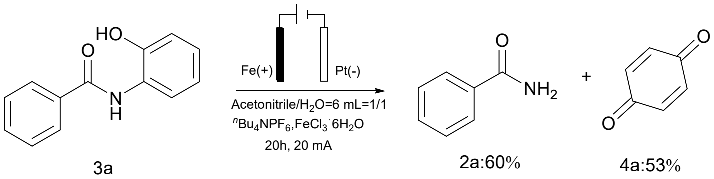

Eq(S2)

We have tried to collection intermediate product by reduce reaction time, *N*– (2–hydroxyphenyl)benzamide (**3a**) and 1,4–benzoquinone (**4a**) were observed by GC/Mass (**Eq S1**). And according to optimised condition, product **2a** was obtained in 60 % yield when 3a was used as starting material (**Eq S2**). **3a**： **^{1}H NMR** (400 MHz, $CDCl_3$) δ 8.64 (s, 1H), 8.12 (s, 1H), 8.00 – 7.90 (m, 2H), 7.68 – 7.50 (m, 3H), 7.20 (dd, *J* = 12.3, 4.6 Hz, 2H), 7.11 (d, *J* = 8.0 Hz, 1H), 6.95 (td, *J* = 7.6, 1.4 Hz, 1H), 5.32 (s, 2H); **4a: ^{1}H NMR** (400 MHz, $CDCl_3$) δ 7.28 (s, 4H).

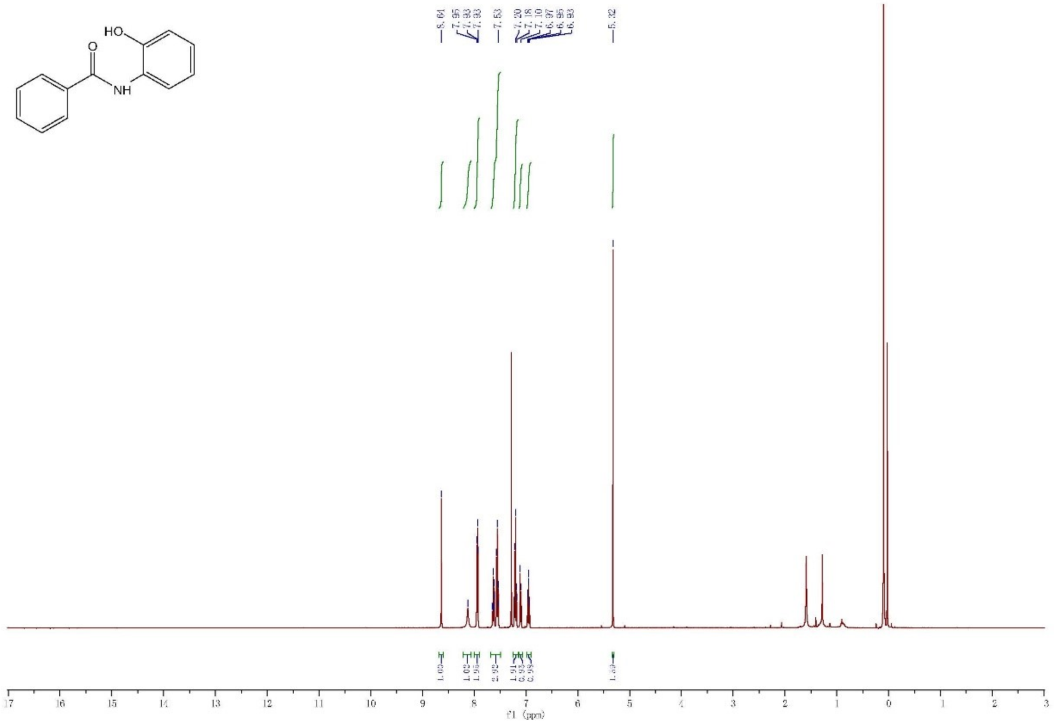

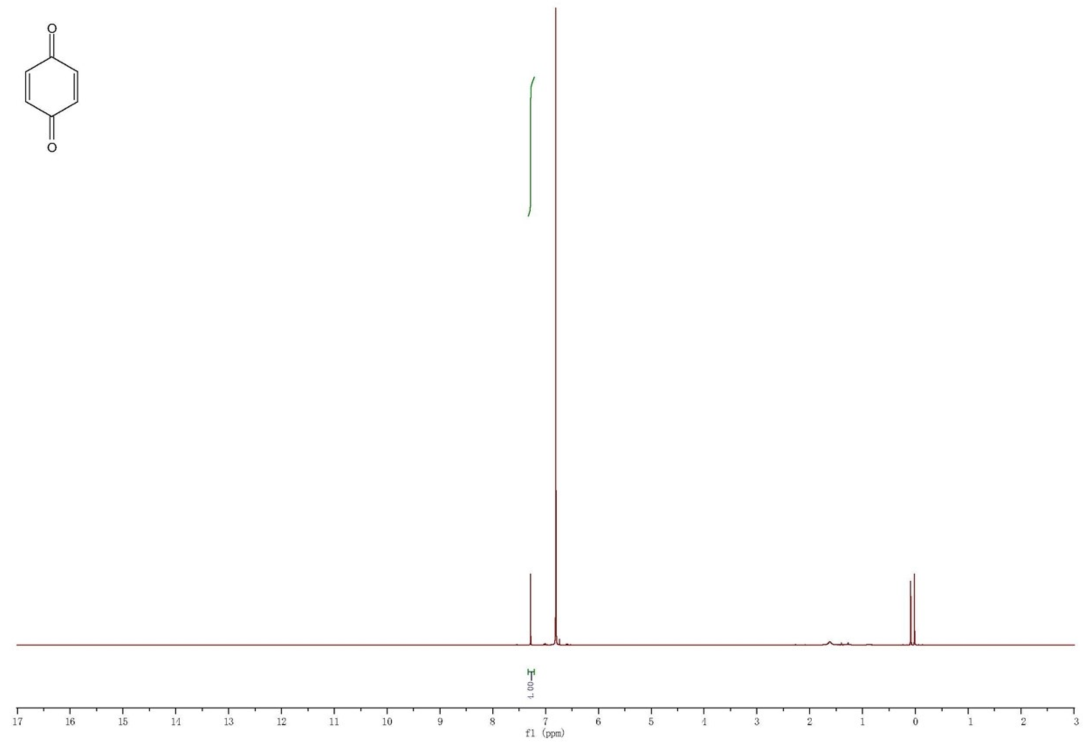

Scheme S4: Steric hindrance effect:

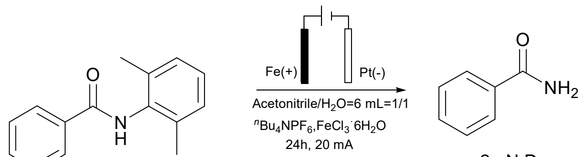

1b 2a:N.D

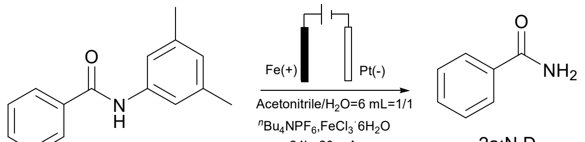

1c 2a:N.D

To elucidate the mechanism of this reaction, some reactive site were instead by methyl under the standard conditions. The experiments indicate that Steric hindrance has been shown to affect reaction of ortho C-H activation and electronic attack.

Table S2: Radical scavenger experiments[a]:

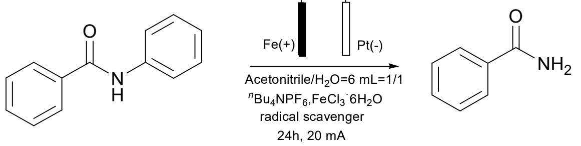

Entry	Scavenger	Equivalent	Yields[b]
1	1,1-diphenylethylene	0.1	66
	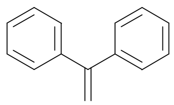	0.2	42
		0.5	18
		1.0	Trace
		2.0	Trace

2	BHT<br>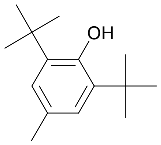	0.1<br>0.2<br>0.5<br>1.0<br>2.0	39<br>23<br>Trace<br>n.d<br>n.d
3	TEMPO<br>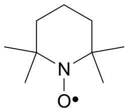	0.1<br>0.2<br>0.5<br>1.0<br>2.0	72<br>55<br>30<br>Trace<br>trace

[a] Standard conditions: substrate (0.6 mmol), H_2O (3 mL), $^nBu_4NPF_6$ (0.05 M), $FeCl_3{\cdot}6H_2O$ (0.2 equiv), MeCN/H_2O (6 mL, 1: 1), air, Pt net and Fe plate electrodes (1.0 cm*1.0 cm^2), 24 h, room temperature and constant current electrolysis at 20 mA in an undivided cell; [b] Yields were determined using GC/Mass analysis with 1H-benzo[*d*]imidazole as an internal standard.

Cyclic voltammetry experiment

Cyclic voltammograms were measured using Shanghai chenhua CHI600E electrochemical workstation with electrochemical analysis software, using a conventional three-electrode cell. The working electrode was a Pt disk electrode, The counter and reference electrodes consisted of a Pt wire and a calomel electrode, espectively. The Pt disk working electrode was polished with a polishing cloth before each measurement. the concentration of all tested compounds was 0.1M, if not noted otherwise. The scan rate was 0.1 V/s.

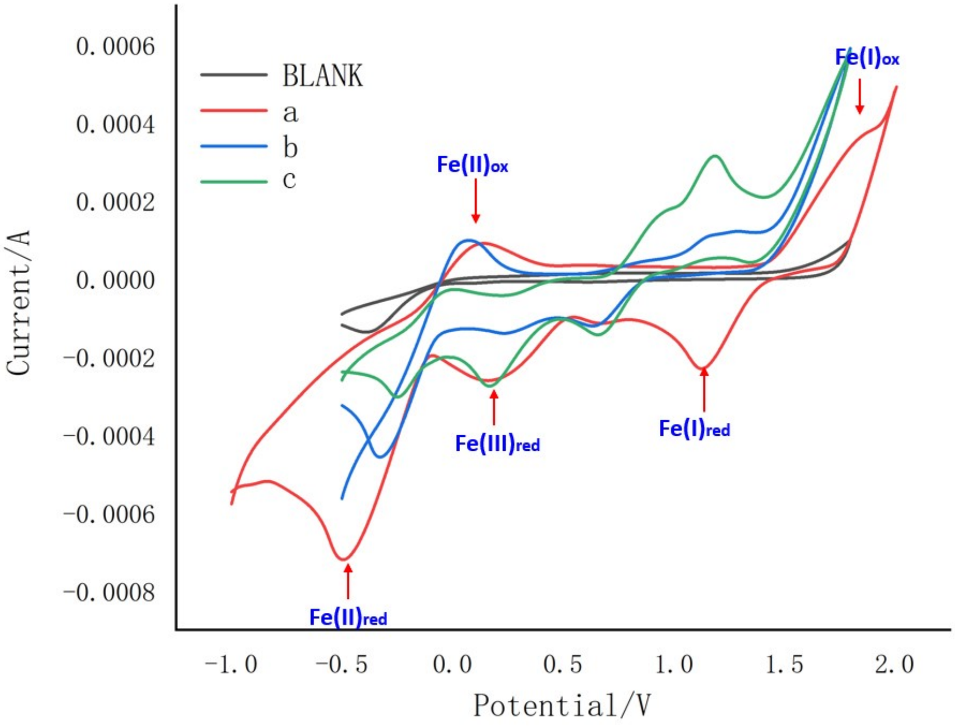

Figure S2. Blank: 0.1M nBu_4NPF_6, MeCN /H2O= 1/1; a: Blank + $FeCl_3{\cdot}6H_2O$ 0.02 mmol; b: Blank + 1a 0.1mmol + $FeCl_3{\cdot}6H_2O$ 0.02 mmol; c: Blank + 3a 0.1 mmol + $FeCl_3{\cdot}6H_2O$ 0.02 mmol

Scheme S5: Deuterium-labeling experiment

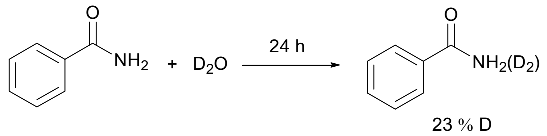

A solution of the benzamide (0.3 mmol) in 3 mL of MeCN was treated with D_2O (3 mL). The solution was allowed to stir at room temperature for 24 hours. After the solution was evaporated in vacuo. The product was confirmed by nuclear magnetic resonance and deuterated benzamide at the 23 % D level.

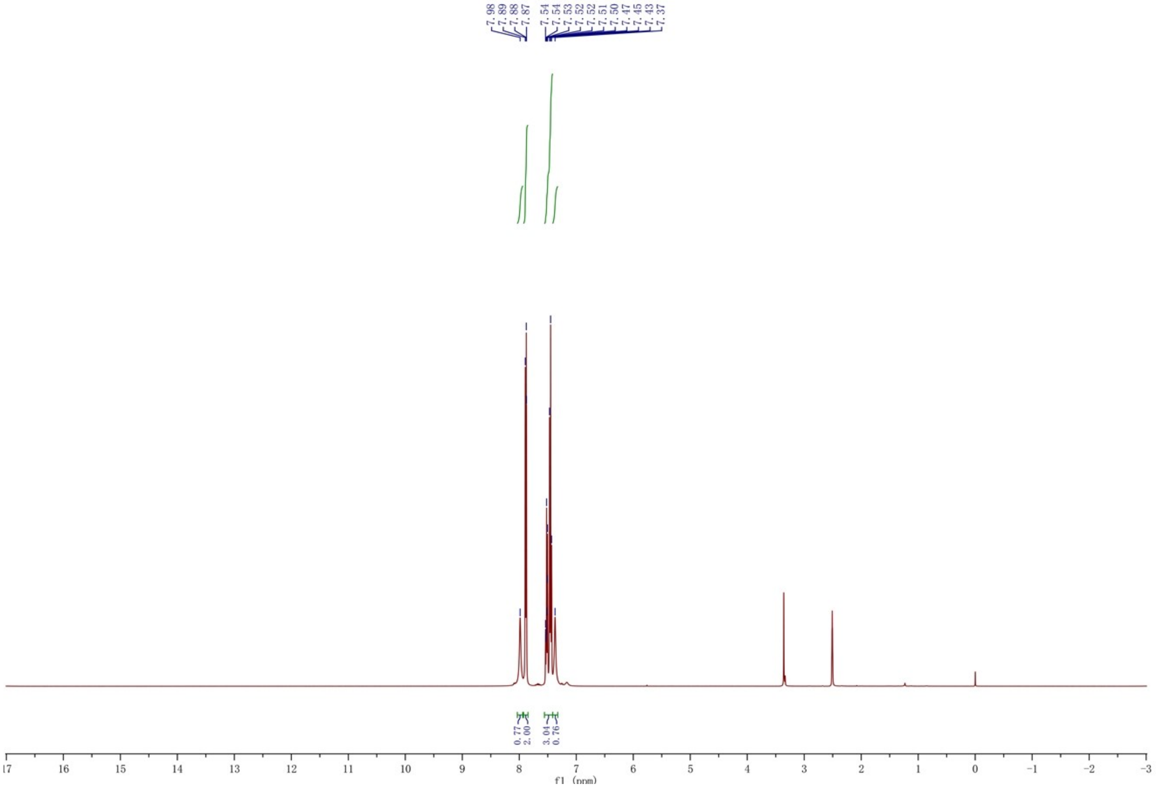

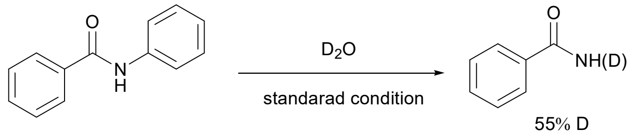

An undivided cell was equipped with a magnet stirrer, Fe plate (1.0 *1.0 cm^2), Pt net (1.0*10 cm^2), as anode electrode and cathode electrode. The substrate secondary phenylamide (0.6 mmol), $^nBu_4NPF_6$ (0.05 M), $FeCl_3{\cdot}6H_2O$ (20 %) , 3.0 mL D_2O and 3.0 mL MeCN were placed. The resulting mixture was allowed to stir and electrolyze at constant current conditions (20 mA) at room temperature for 24 hours. Then the reaction mixture was poured into water (10 mL) and extracted with ethyl acetate (10 mL*3). The residue was purified by silica gel column chromatography to obtain the corresponding product. The product was confirmed by nuclear magnetic resonance and deuterated benzamide at the 55 % D level.

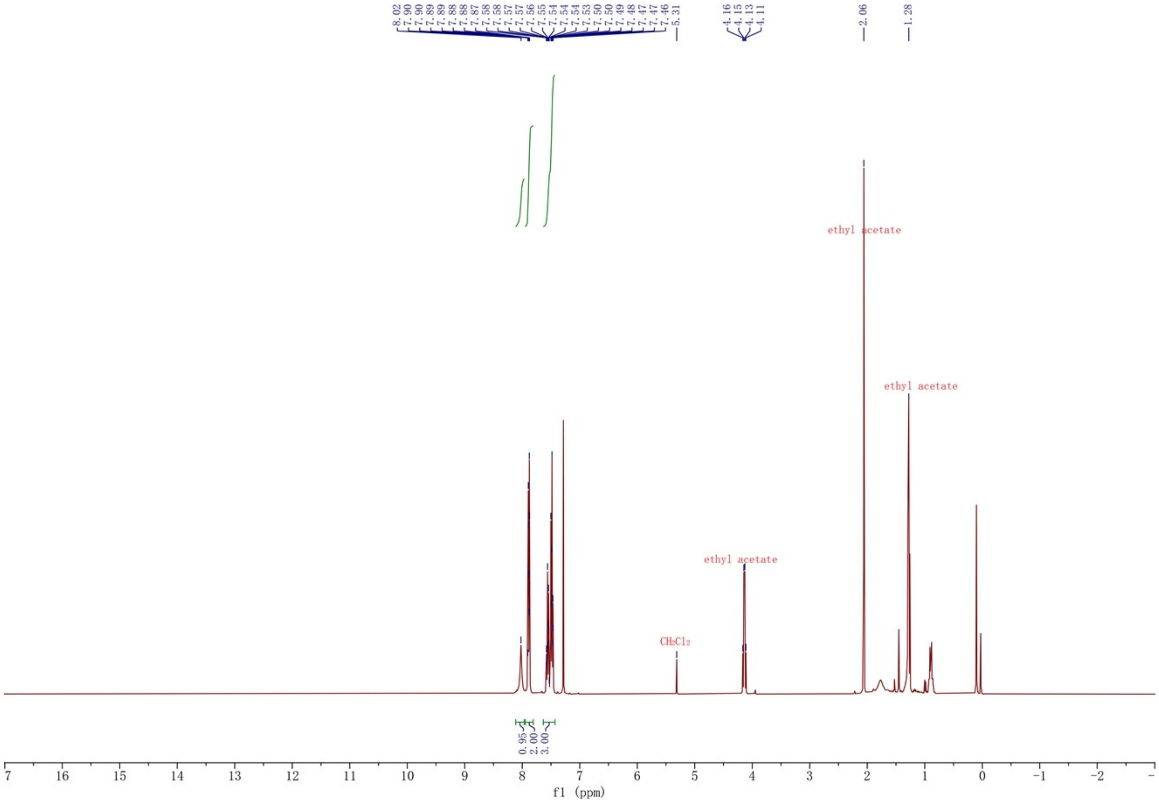

Scheme S6: Procedure for synthesis of Trimethobenzamide

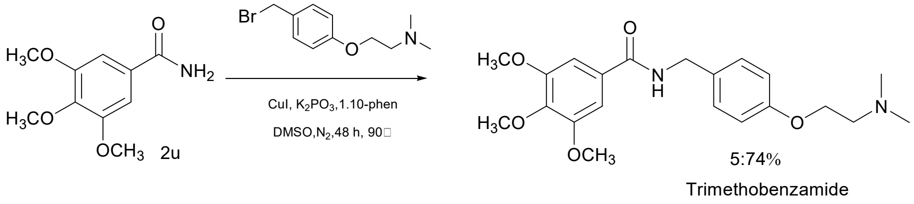

CuI (0.10 equiv), K_2PO_3 (3.0 equiv), 1,10–phenanthroline (0.2 equiv), DMSO (2.0 mL), 3,4,5–trimethoxybenzamide (1.0 equiv) and 2– (4– (bromomethyl)phenoxy) –N,N–dimethylethanamine (1.1 equiv) were added to a Schlenk flask. The reaction mixture was allowed to stir at 90 °C for 48 h. After allowing the reaction to cool to ambient temperature, the mixture was dissolved in MeOH (10 mL) and concentrated under reduced pressure. The crude residue was directly purified by silica gel column chromatography in 72% yield. **3aa:** ^{1}**H NMR** (400 MHz, DMSO) δ 9.23 (t, *J* = 5.9 Hz, 1H), 7.37 – 7.23 (m, 4H), 6.94 (d, *J* = 8.6 Hz, 2H), 4.42 (d, *J* = 5.8 Hz, 2H), 4.26 (t, *J* = 5.3 Hz, 2H), 3.84 (s, 6H), 3.71 (s, 3H), 3.22 (t, *J* = 5.3 Hz, 2H), 2.63 (d, *J* = 5.4 Hz, 6H); ^{13}**C NMR** (101 MHz, DMSO) δ 166.82, 166.06, 165.85, 157.15, 153.00, 140.33, 132.94, 129.95, 129.20, 114.88, 105.38, 63.90, 60.51, 56.50, 56.28, 43.92, 42.56; **MS [EI, m/z]**: 388.20 [M+].

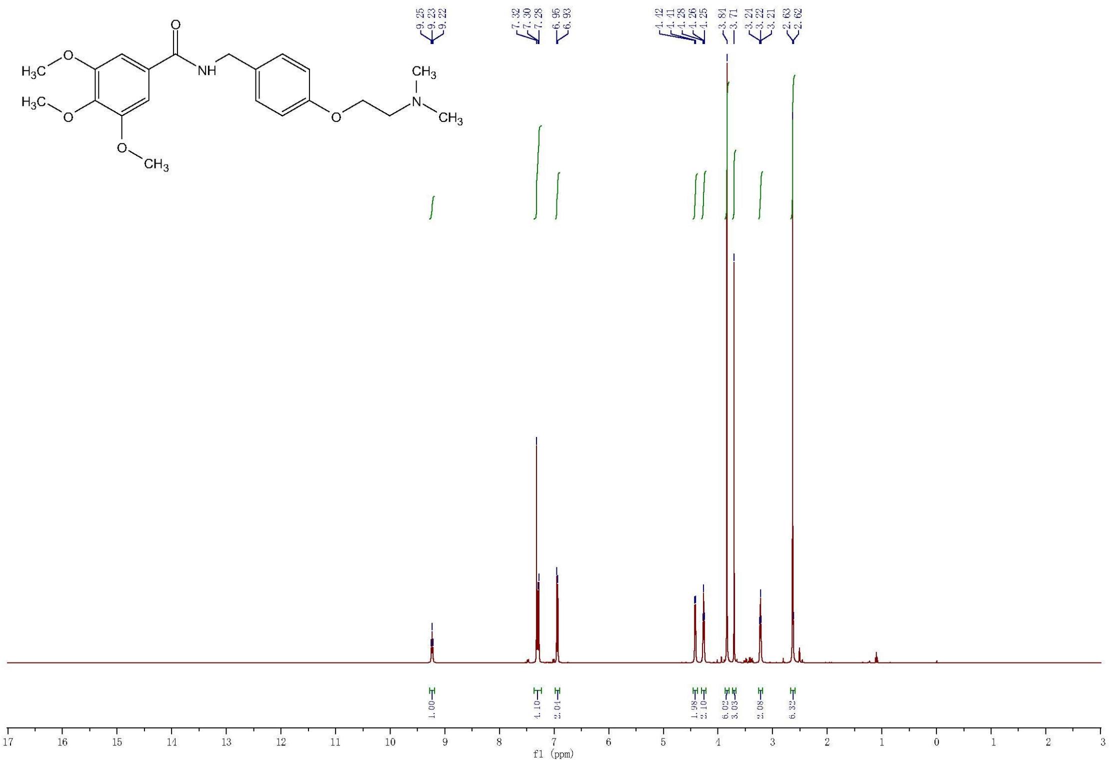

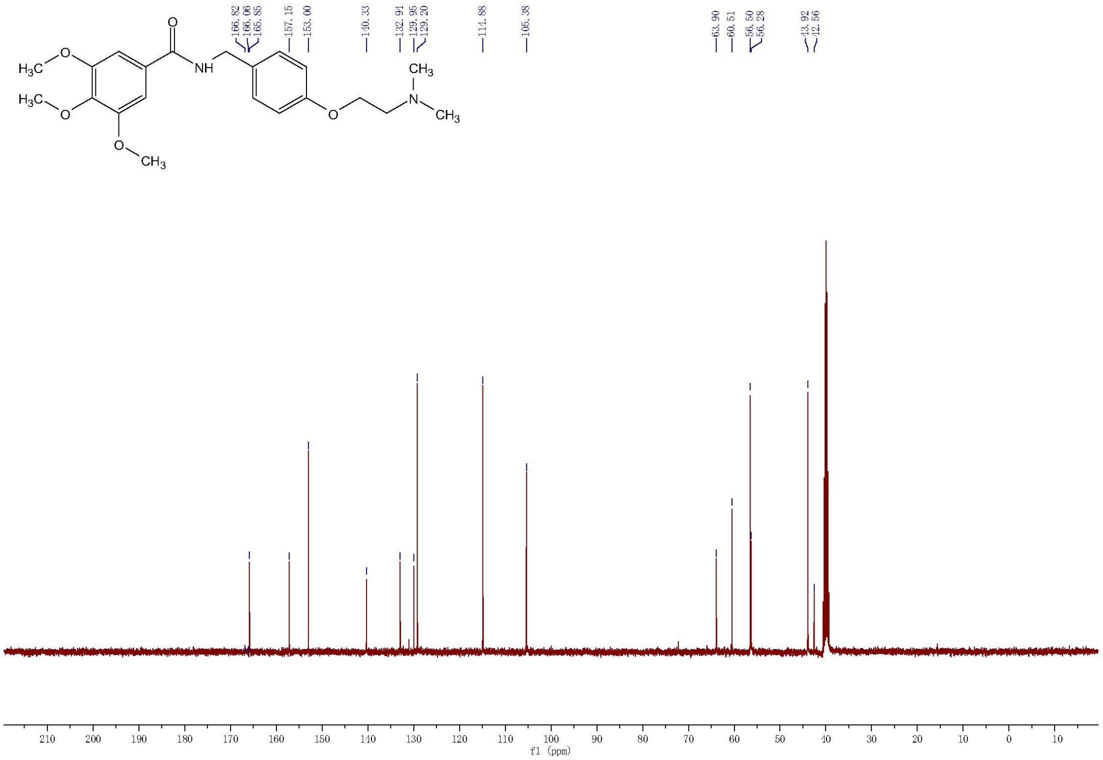

Scheme S7: H_2O^{18} experiment

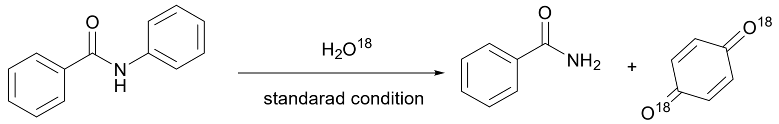

An undivided cell was equipped with a magnet stirrer, Fe plate (1.0 *1.0 cm^2), Pt net (1.0*10 cm^2), as anode electrode and cathode electrode. The substrate secondary phenylamide (0.6 mmol), $^{n}Bu_4NPF_6$ (0.05 M), $FeCl_3·6H_2O$ (20 %) , 3.0 mL of water, 100 uL H_2O^{18} and 3.0 mL MeCN were placed. The resulting mixture was allowed to stir at constant current conditions (20 mA) at room temperature for 24 hours. Then the reaction mixture was poured into water (10 mL) and extracted with ethyl acetate (10 mL*3). The residue was confirmed by Gas chromatography–mass spectra.

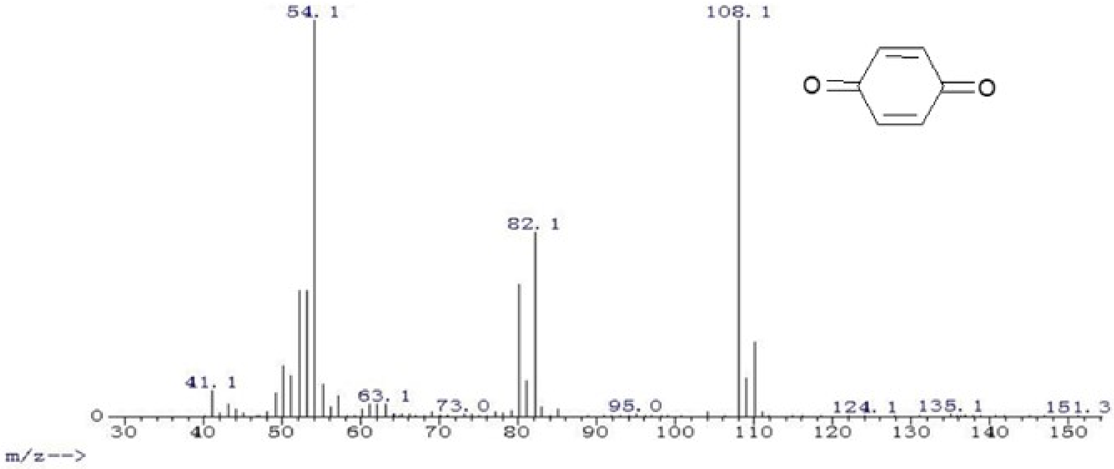

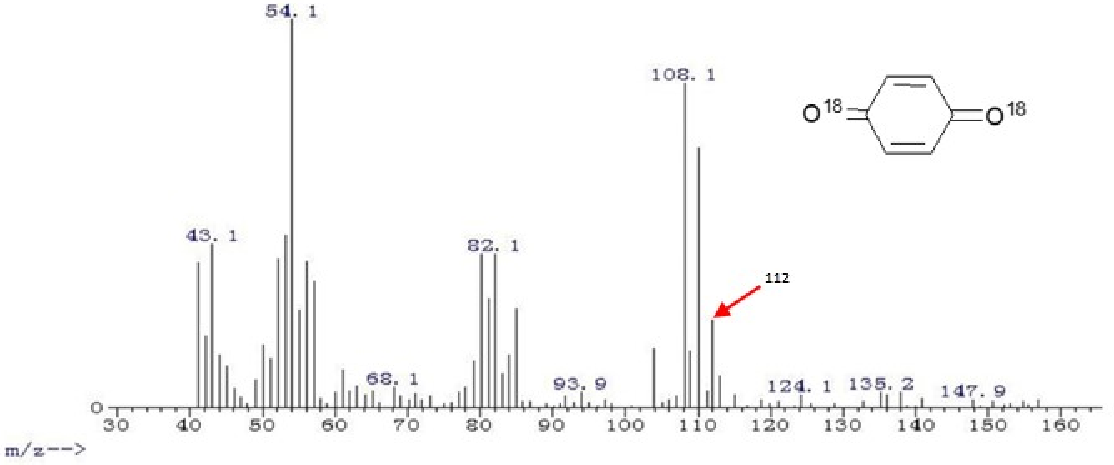

Experimental procedures and characterisation data

2a: benzamide[1]

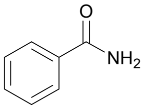

^{1}H NMR (400 MHz, $CDCl_3$) δ 7.84 (d, *J* = 7.1 Hz, 2H), 7.55 (t, *J* = 7.4 Hz, 1H), 7.46 (t, *J* = 7.5 Hz, 2H), 6.22 (s, 2H)；**^{13}C NMR** (101 MHz, $CDCl_3$) δ 169.62, 133.36, 132.01, 128.63, 127.39; **HRMS (ESI) m/z**: $[M+H]^+$ Calcd for C_7H_8NO 122.0606; Found 122.0612.

2b: 2-aminobenzamide[2]

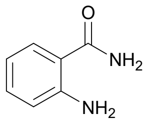

^{1}H NMR (400 MHz, DMSO) δ 7.72 (s, 1H), 7.53 (dd, *J* = 8.0, 1.4 Hz, 1H), 7.13 (ddd, *J* = 8.4, 7.1, 1.5 Hz, 1H), 7.07 (s, 1H), 6.68 (dd, *J* = 8.2, 1.0 Hz, 1H), 6.56 (s, 2H), 6.48 (ddd, *J* = 8.1, 7.1, 1.2 Hz, 1H); **^{13}C NMR** (101 MHz, DMSO) δ 171.76, 150.65, 132.35, 129.20, 116.85, 114.82, 114.12; **HRMS (ESI) m/z**: $[M+H]^+$ Calcd for $C_7H_9N_2O$ 137.0709; Found 137.0701.

2c: 2-methoxybenzamide[3]

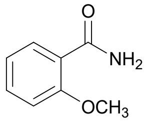

^{1}H NMR (400 MHz, DMSO) δ 7.81 (dd, *J* = 7.7, 1.8 Hz, 1H), 7.64 (s, 1H), 7.56 – 7.44 (m, 2H), 7.13 (d, *J* = 7.9 Hz, 1H), 7.03 (td, *J* = 7.6, 1.0 Hz, 1H), 3.89 (s, 3H); **^{13}C NMR** (101 MHz, DMSO) δ 166.75, 157.67, 132.89, 131.17, 123.17, 120.83, 112.41, 56.25; **HRMS (ESI) m/z**: $[M+H]^+$ Calcd for $C_8H_{10}NO_2$ 152.0706; Found 152.0711.

2d: 2-bromobenzamide[3]

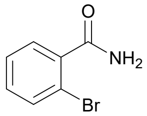

^{1}H NMR (400 MHz, DMSO) δ 7.87 (s, 1H), 7.66 – 7.62 (m, 1H), 7.57 (s, 1H), 7.45 – 7.39 (m, 2H), 7.34 (ddd, *J* = 8.0, 6.1, 3.1 Hz, 1H); **^{13}C NMR** (101 MHz, DMSO) δ 169.51, 139.82, 133.15, 131.09, 129.00, 127.94, 119.06; **HRMS (ESI) m/z**: $[M+H]^+$ Calcd for C_7H_7BrNO 199.9711; Found 199.9719.

2e: 2-chlorobenzamide[2]

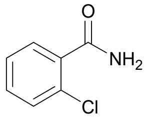

^{1}H NMR (400 MHz, DMSO) δ 7.87 (s, 1H), 7.58 (s, 1H), 7.50 – 7.35 (m, 4H); **^{13}C NMR** (101 MHz, DMSO) δ 168.61, 137.62, 131.00, 130.08, 130.05, 129.11, 127.47; **HRMS (ESI) m/z**: $[M+H]^+$ Calcd for C_7H_7ClNO 156.0211; Found 156.0209.

2f: 2-iodobenzamide[4]

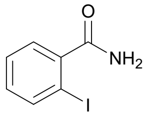

^{1}H NMR (400 MHz, DMSO) δ 7.87 (dd, *J* = 7.9, 0.9 Hz, 1H), 7.82 (s, 1H), 7.51 (s, 1H), 7.43 (td, *J* = 7.5, 1.1 Hz, 1H), 7.34 (dd, *J* = 7.6, 1.7 Hz, 1H), 7.18 – 7.12 (m, 1H); **^{13}C NMR** (101 MHz, DMSO) δ 171.15, 143.60, 139.61, 131.04, 128.38, 128.23, 93.59; **HRMS (ESI) m/z**: $[M+H]^+$ Calcd for C_7H_7INO 247.9572; Found 247.9573.

2g: 4-aminobenzamide[2]

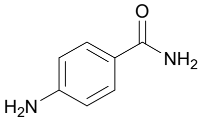

^{1}H NMR (400 MHz, DMSO) δ 7.62 – 7.55 (m, 2H), 7.50 (s, 1H), 6.82 (s, 1H), 6.58 – 6.46 (m, 2H), 5.59 (s, 2H); **^{13}C NMR** (101 MHz, DMSO) δ 168.47, 152.11, 129.57, 121.43, 112.90; **HRMS (ESI) m/z**: $[M+H]^+$ Calcd for $C_7H_8N_2O$ 137.0709; Found 137.0707.

2h: 2-nitrobenzamide[3]

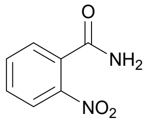

^{1}H NMR (400 MHz, DMSO) δ 8.14 (s, 1H), 8.00 (dd, *J* = 8.0, 1.1 Hz, 1H), 7.77 (td, *J* = 7.5, 1.2 Hz, 1H), 7.73 – 7.59 (m, 3H); **^{13}C NMR** (101 MHz, DMSO) δ 167.60, 147.70, 133.78, 133.04, 131.07, 129.30, 124.40; **HRMS (ESI) m/z**: $[M+Na]^+$ Calcd for $C_7H_6N_2O_3Na$ 189.0271; Found 189.0270.

2i: 2-iodo-5-methylbenzamide[5]

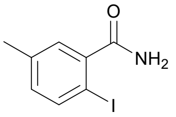

^{1}H NMR (400 MHz, DMSO) δ 7.77 (s, 1H), 7.72 (d, *J* = 8.0 Hz, 1H), 7.47 (s, 1H), 7.18 (d, *J* = 1.7 Hz, 1H), 7.01 – 6.94 (m, 1H), 2.28 (s, 3H); **^{13}C NMR** (101 MHz, DMSO) δ 171.15, 143.40, 139.39, 138.00, 131.81, 128.98, 89.50, 20.79; **HRMS (ESI) m/z**: $[M+Na]^+$ Calcd for C_8H_8INONa 283.9548; Found 283.9550.

2j: 3-aminobenzamide[6]

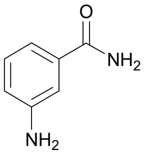

^{1}H NMR (400 MHz, DMSO) δ 7.71 (s, 1H), 7.23 – 6.95 (m, 4H), 6.68 (ddd, *J* = 8.0, 2.3, 1.0 Hz, 1H), 5.18 (s, 2H); **^{13}C NMR** (101 MHz, DMSO) δ 169.16, 149.03, 135.67, 128.97, 116.91, 115.11, 113.56; **HRMS (ESI) m/z**: $[M+H]^+$ Calcd for $C_7H_8N_2O$ 137.0709; Found 137.0715.

2k: 3-methylbenzamide[3]

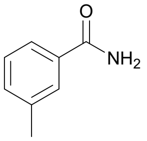

^{1}H NMR (400 MHz, DMSO) δ 7.92 (s, 1H), 7.74 – 7.62 (m, 2H), 7.33 (d, *J* = 4.6 Hz, 3H), 2.35 (s, 3H); **^{13}C NMR** (101 MHz, DMSO) δ 168.45, 137.87, 134.71, 132.20, 128.53, 128.52, 125.03, 21.41; **HRMS (ESI) m/z**: $[M+H]^+$ Calcd for $C_8H_{10}NO$ 136.0757; Found 136.0755.

2l: 3-fluorobenzamide[3]

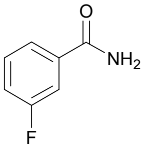

^{1}H NMR (400 MHz, DMSO) δ 8.07 (s, 1H), 7.76 – 7.70 (m, 1H), 7.67 (ddd, *J* = 10.1, 2.5, 1.6 Hz, 1H), 7.58 – 7.46 (m, 2H), 7.42 – 7.33 (m, 1H); **^{13}C NMR** (101 MHz, DMSO) δ 166.94 (d, *J* = 2.4 Hz), 162.42 (d, *J* = 244.42 Hz), 137.16 (d, *J* = 6.6 Hz) 130.84 (d, *J* = 8.0 Hz) 124.06 (d, *J* = 2.8 Hz), 118.57 (d, *J* = 21.2 Hz), 114.65 (d, *J* = 22.4 Hz); **HRMS (ESI) m/z**: $[M+H]^+$ Calcd for C_7H_7FNO 140.0506; Found 140.0505.

2m: 4-methylbenzamide[3]

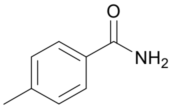

^{1}H NMR (400 MHz, DMSO) δ 7.89 (s, 1H), 7.77 (d, *J* = 8.2 Hz, 2H), 7.25 (d, *J* = 7.9 Hz, 3H), 2.35 (s, 3H); **^{13}C NMR** (101 MHz, DMSO) δ 168.21, 141.49, 131.94, 129.18, 127.95, 21.41; **HRMS (ESI) m/z**: $[M+H]^+$ Calcd for $C_8H_{10}NO$ 136.0762; Found 136.0760.

2n: 4-methoxybenzamide[3]

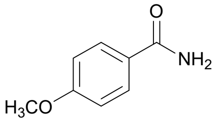

^{1}H NMR (400 MHz, DMSO) δ 7.90 – 7.79 (m, 3H), 7.18 (s, 1H), 7.00 – 6.95 (m, 2H), 3.81 (s, 3H); **^{13}C NMR** (101 MHz, DMSO) δ 167.86, 162.02, 129.79, 126.97, 113.83, 55.77; **HRMS (ESI) m/z**: $[M+H]^+$ Calcd for $C_8H_{10}NO_2$ 152.0711; Found 152.0722.

2o: 4-chlorobenzamide[2]

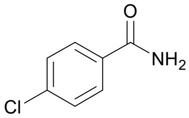

^{1}H NMR (400 MHz, DMSO) δ 8.05 (s, 1H), 7.92 – 7.86 (m, 2H), 7.56 – 7.51 (m, 2H), 7.47 (s, 1H); **^{13}C NMR** (101 MHz, DMSO) δ 167.24, 136.53, 133.48, 129.86, 128.76; **HRMS (ESI) m/z**: $[M+H]^+$ Calcd for C_7H_7ClNO 156.0211; Found 156.0201.

2p: 4-acetylbenzamide[2]

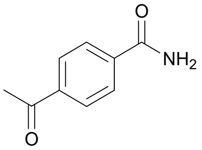

^{1}H NMR (400 MHz, DMSO) δ 8.15 (s, 1H), 8.04 – 7.97 (m, 4H), 7.57 (s, 1H), 2.62 (s, 3H); **^{13}C NMR** (101 MHz, DMSO) δ 198.20, 167.53, 139.10, 138.56, 128.55, 128.21, 27.44; **HRMS (ESI) m/z**: $[M+H]^+$ Calcd for $C_9H_{10}NO_2$ 164.0706; Found 164.0701.

2q: 4-(dimethylamino)benzamide[6]

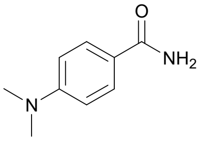

^{1}H NMR (400 MHz, DMSO) δ 7.76 – 7.71 (m, 2H), 7.63 (s, 1H), 6.92 (s, 1H), 6.70 – 6.66 (m, 2H), 2.96 (s, 6H); **^{13}C NMR** (101 MHz, DMSO) δ 168.36, 152.57, 129.36, 121.41, 111.16, 40.19; **HRMS (ESI) m/z**: $[M+H]^+$ Calcd for $C_9H_{12}N_2O$ 165.1022; Found 165.1019.

2r: 4-phenyl benzamide[6]

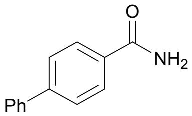

^{1}H NMR (400 MHz, DMSO) δ 7.96 (d, *J* = 8.3 Hz, 2H), 7.72 – 7.67 (m, 2H), 7.63 (d, *J* = 8.1 Hz, 2H), 7.47 (t, *J* = 7.6 Hz, 2H), 7.37 (t, *J* = 7.3 Hz, 1H); **^{13}C NMR** (101 MHz, DMSO) δ 169.29, 140.36, 130.14, 129.40, 128.04, 127.27, 127.20, 126.37, 126.32; **HRMS (ESI) m/z**: $[M+H]^+$ Calcd for $C_{13}H_{12}NO$ 198.0919; Found 198.0922.

2s: 4-nitrobenzamide[3]

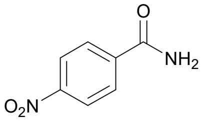

^{1}H NMR (400 MHz, DMSO) δ 8.34 – 8.26 (m, 3H), 8.12 – 8.06 (m, 2H), 7.74 (s, 1H); **^{13}C NMR** (101 MHz, DMSO) δ 166.66, 149.50, 140.43, 129.37, 123.91; **HRMS (ESI) m/z**: $[M+H]^+$ Calcd for $C_7H_7N_2O_3$ 167.0451; Found 167.0458.

2t: 4-(trifluoromethyl)benzamide[3]

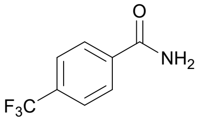

^{1}H NMR (400 MHz, DMSO) δ 8.20 (s, 1H), 8.07 (d, *J* = 8.0 Hz, 2H), 7.84 (d, *J* = 8.2 Hz, 2H), 7.63 (s, 1H); **^{13}C NMR** (101 MHz, DMSO) δ 167.13, 138.55, 131.61 (q, *J* = 31.8 Hz), 128.78, 125.73 (q, *J* = 3.8 Hz), 123.07; **HRMS (ESI) m/z**: $[M+Na]^+$ Calcd for $C_8H_7F_3NONa$ 212.0299; Found 212.0297.

2u: 3,4,5-trimethoxybenzamide[7]

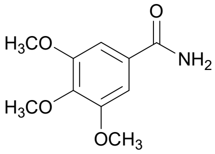

^{1}H NMR (400 MHz, DMSO) δ 7.95 (s, 1H), 7.33 (s, 1H), 7.22 (s, 2H), 3.82 (s, 6H), 3.70 (s, 3H); **^{13}C NMR** (101 MHz, DMSO) δ 167.75, 152.95, 140.40, 129.90, 105.51, 60.50, 56.42; **HRMS (ESI) m/z**: [M+H]$^+$ Calcd for $C_{10}H_{13}NO_4$ 212.0928; Found 212.0929.

2v: 4-ethylbenzamide[15]

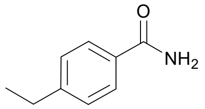

^{1}H NMR (400 MHz, DMSO) δ 7.90 (s, 1H), 7.81 (d, *J* = 8.2 Hz, 2H), 7.28 (d, *J* = 8.2 Hz, 3H), 2.64 (q, *J* = 7.6 Hz, 2H), 1.19 (t, *J* = 7.6 Hz, 3H); **^{13}C NMR** (101 MHz, DMSO) δ 168.28, 147.66, 132.23, 128.04, 128.00, 28.48, 15.82; **HRMS (ESI) m/z**: [M+Na]$^+$ Calcd for $C_9H_{11}NONa$ 172.0738; Found 172.0733.

2w: thiophene-3-carboxamide[8]

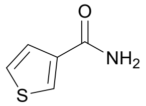

^{1}H NMR (400 MHz, DMSO) δ 8.13 (dd, *J* = 3.0, 1.3 Hz, 1H), 7.78 (s, 1H), 7.56 (dd, *J* = 5.0, 3.0 Hz, 1H), 7.48 (dd, *J* = 5.0, 1.3 Hz, 1H), 7.23 (s, 1H); **^{13}C NMR** (101 MHz, DMSO) δ 164.12, 138.47, 129.45, 127.61, 127.00; **HRMS (ESI) m/z**: [M+H]$^+$ Calcd for C_5H_5NOS 128.0165; Found 125.0168.

2x: nicotinamide[2]

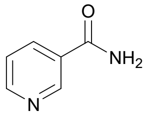

^{1}H NMR (400 MHz, DMSO) δ 9.04 (dd, *J* = 2.2, 0.7 Hz, 1H), 8.70 (dd, *J* = 4.8, 1.7 Hz, 1H), 8.24 – 8.13 (m, 2H), 7.61 (s, 1H), 7.50 (ddd, *J* = 7.9, 4.8, 0.8 Hz, 1H); **^{13}C NMR** (101 MHz, DMSO) δ 166.90, 152.35, 149.13, 135.60, 130.11, 123.86; **HRMS (ESI) m/z**: [M+H]$^+$ Calcd for $C_6H_7N_2O$ 123.0558; Found 123.0555.

2y: 2-(2-oxopyrrolidin-1-yl)acetamide[13]

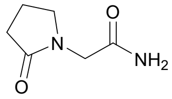

^{1}H NMR (400 MHz, DMSO) δ 7.39 (s, 1H), 7.09 (s, 1H), 3.75 (s, 2H), 3.36 (t, *J* = 7.1 Hz, 2H), 2.27 – 2.18 (m, 2H), 1.98 – 1.88 (m, 2H); **^{13}C NMR** (101 MHz, DMSO) δ 174.86, 170.16, 47.73, 45.27, 30.43, 17.80; **HRMS (ESI) m/z**: [M+Na]$^+$ Calcd for $C_6H_{10}N_2O_2Na$ 165.0634; Found 165.0634.

2z: 2-naphthamide[2]

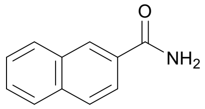

^{1}H NMR (400 MHz, DMSO) δ 8.50 (s, 1H), 8.16 (s, 1H), 8.06 – 7.93 (m, 4H), 7.66 – 7.55 (m, 2H), 7.49 (s, 1H); **^{13}C NMR** (101 MHz, DMSO) δ 168.43, 134.62, 132.61, 132.10, 129.32, 128.27, 128.22, 128.04, 128.02, 127.10, 124.86; **HRMS (ESI) m/z**: [M+H]$^+$ Calcd for $C_{11}H_{10}NO$ 172.0757; Found 172.0756.

2aa: cyclopropanecarboxamide[2]

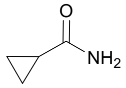

^{1}H NMR (400 MHz, DMSO) δ 7.51 (s, 1H), 6.75 (s, 1H), 1.51 (tt, *J* = 7.3, 5.2 Hz, 1H), 1.20 (dd, *J* = 17.1, 9.9 Hz, 1H), 0.63 (dd, *J* = 6.0, 4.6 Hz, 3H); **^{13}C NMR** (101 MHz, DMSO) δ175.09, 13.69, 6.66; **HRMS (ESI) m/z**: [M+H]$^+$ Calcd for C_4H_8NO 86.0605; Found 86.0599.

2bb: 2,2,3,3-tetramethylcyclopropanecarboxamide[9]

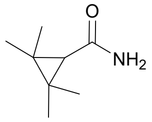

^{1}H NMR (400 MHz, DMSO) δ 7.21 (s, 1H), 6.48 (s, 1H),1.18 (s, 6H), 1.14 (s, 6H), 1.12 (s, 1H); **^{13}C NMR** (101 MHz, DMSO) δ 173.36, 35.30, 23.72, 16.90; **HRMS (ESI) m/z**: [M+H]$^+$ Calcd for $C_8H_{16}NO$ 142.1226; Found 142.1223.

2cc: 3,3-dimethylbutanamide[10]

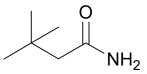

^{1}H NMR (400 MHz, DMSO) δ 7.31 – 7.06 (m, 1H), 6.61 (d, *J* = 42.5 Hz, 1H), 1.92 (s, 2H), 0.96 (s, 9H); **^{13}C NMR** (101 MHz, DMSO) δ 173.49, 48.98, 30.63, 30.16; **HRMS (ESI) m/z**: [M+Na]$^+$ Calcd for $C_6H_{13}NONa$ 138.0895; Found 138.0891.

2dd: 5-methylhexanamide[11]

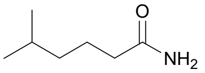

^{1}H NMR (400 MHz, DMSO) δ 7.61 (s, 1H), 7.12 (s, 1H), 3.32 – 3.19 (m, 1H), 2.46 (t, *J* = 7.2 Hz, 2H), 1.75 – 1.37 (m, 4H), 0.81 (dt, *J* = 11.2, 7.4 Hz, 6H); **^{13}C NMR** (101 MHz, DMSO) δ 170.99, 42.78, 21.44, 16.97, 13.93, 12.21; **HRMS (ESI) m/z**: [M+H]$^+$ Calcd for $C_7H_{16}NO$ 130.1226; Found 130.1223.

2ee: 6,7-dimethyloctanamide[12]

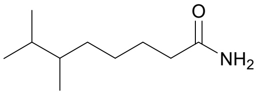

^{1}H NMR (400 MHz, DMSO) δ 7.67 (s, 1H), 7.13 (s, 1H), 3.46 (dd, *J* = 8.7, 5.5 Hz, 1H), 2.53 – 2.50 (m, 3H), 1.69 – 1.08 (m, 6H), 0.86 – 0.82 (m, 9H); **^{13}C NMR** (101 MHz, DMSO) δ 171.08, 38.66, 37.04, 27.46, 26.04, 23.34, 22.78, 22.67, 22.45; **HRMS (ESI) m/z**: [M+Na]$^+$ Calcd for $C_{10}H_{21}NONa$ 194.1521; Found 194.1518.

2ff: 2-phenylacetamide[6]

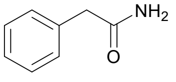

^{1}H NMR (400 MHz, DMSO) δ 7.47 (s, 1H), 7.32 – 7.19 (m, 5H), 6.89 (s, 1H), 3.37 (s, 2H); **^{13}C NMR** (101 MHz, DMSO) δ 172.67, 136.96, 129.51, 128.60, 126.71, 42.72; **HRMS (ESI) m/z**: [M+Na]$^+$ Calcd for C_8H_9NONa 158.0582; Found 158.0576.

2gg: 4-phenylbutanamide[3]

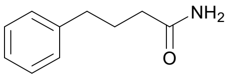

^{1}H NMR (400 MHz, DMSO) δ 7.32 – 7.23 (m, 3H), 7.21 – 7.15 (m, 3H), 6.74 (s, 1H), 2.61 – 2.53 (m, 2H), 2.07 (t, *J* = 7.5 Hz, 2H), 1.85 – 1.73 (m, 2H); **^{13}C NMR** (101 MHz, DMSO) δ 174.48, 142.29, 128.76, 128.72, 126.18, 35.17, 35.01, 27.35; **HRMS (ESI) m/z**: [M+Na]$^+$ Calcd for $C_{10}H_{13}NONa$ 186.0895; Found 186.0890.

2hh: 2-(naphthalen-1-yl)acetamide[6]

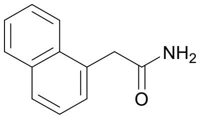

^{1}H NMR (400 MHz, DMSO) δ 8.13 – 8.05 (m, 1H), 7.93 (dd, *J* = 7.1, 2.3 Hz, 1H), 7.82 (dd, *J* = 7.4, 1.8 Hz, 1H), 7.60 – 7.41 (m, 5H), 6.99 (s, 1H), 3.87 (s, 2H); **^{13}C NMR** (101 MHz, DMSO) δ 172.59, 133.79, 133.41, 132.48, 128.80, 128.29, 127.43, 126.36, 126.05, 125.96, 125.94, 124.76, 40.62; **HRMS (ESI) m/z**: [M+Na]$^+$ Calcd for $C_{12}H_{12}NO$ 186.0913; Found 186.0916.

2ii: adamantane-1-carboxamide[6]

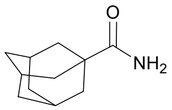

^{1}H NMR (400 MHz, DMSO) δ 6.80 (d, *J* = 111.4 Hz, 2H), 1.94 (s, 3H), 1.74 (d, *J* = 2.7 Hz, 6H), 1.70 – 1.59 (m, 6H); **^{13}C NMR** (101 MHz, DMSO) δ 179.80, 39.30, 39.21, 36.62, 28.14; **HRMS (ESI) m/z**: [M+H]$^+$ Calcd for $C_{11}H_{18}NO$ 180.1388; Found 180.1366.

2jj: (R)-pyrrolidine-2-carboxamide[14]（from (R)-*N*-phenylpyrrolidine-2-carboxamide）

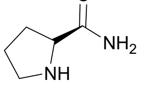

^{1}H NMR (400 MHz, DMSO) δ 7.17 (d, *J* = 144.4 Hz, 2H), 3.42 (dd, *J* = 8.7, 5.5 Hz, 1H), 3.07 – 2.69 (m, 3H), 2.02 – 1.83 (m, 1H), 1.70 – 1.52 (m, 3H); **^{13}C NMR** (101 MHz, DMSO) δ 177.46, 60.67, 47.22, 30.96, 26.29; **HRMS (ESI) m/z**: [M+H]$^+$ Calcd for $C_5H_{11}N_2O$ 115.0866; Found 115.0862.

2kk: 4’-((1,7’-dimethyl-2’-propyl-1H,3’H-[2,5’-bibenzo[d]imidazol]-3’-yl)methyl)-[1,1’-biphenyl]-2-carboxamide[16]

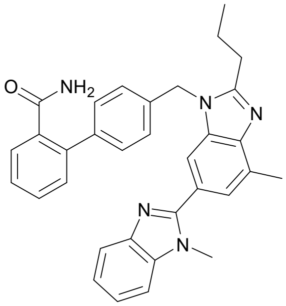

^{1}H NMR (400 MHz, DMSO) δ 7.76 (s, 1H), 7.68 – 7.56 (m, 3H), 7.51 – 7.15 (m, 11H), 5.62 (s, 1H), 3.83 (s, 1H), 2.94 (t, *J* = 7.6 Hz, 1H), 2.64 (s, 1H), 1.91 – 1.76 (m, 1H), 1.02 (t, *J* = 7.4 Hz, 3H); **^{13}C NMR** (101 MHz, DMSO) δ 171.50, 156.63, 154.50, 143.14, 142.96, 140.18, 138.77, 137.81, 137.13, 136.41, 135.17, 130.28, 129.63, 129.19, 128.71, 127.93, 127.50, 126.83, 123.76, 123.68, 122.49, 122.24, 119.15, 110.86, 109.72, 46.49, 32.23, 29.20, 21.21, 16.93, 14.33. **HRMS (ESI) m/z**: $[M+Na]^+$ Calcd for $C_{33}H_{31}N_5ONa$ 536.2421; Found 536.2392.

References

1. B. Xu, Q. Jiang, A. Zhao, J. Jia, Q. Liu, W. Luo and C. Guo, *Chem. Commun.*, 2015, **51**, 11264.
2. H. Chen, W. Dai, Y. Chen, Q. Xu, J. Chen, L. Yu, Y. Zhao, M. Ye and Y. Pan, *Green Chem*., 2014, **16**, 2136.
3. Y.-Q. Miao, J.-X. Kang, Y.-N. Ma and X. Chen, *Green Chem.*, 2021, **23**, 3595.
4. R. G. Chary, G. Dhananjaya, K. V. Prasad, S. Vaishaly, Y. S. S. Ganesh, B. Dulla, K. S. Kumar and M. Pal, *Chem. Commun*., 2014, **50**, 6797.
5. S. B. Munoz, V. Krishnamurti, P. Barrio, T. Mathew and G. K. S. Prakash, *Org. Lett*., 2018, **20**, 1042.
6. J. Chen, Y. Xia and S. Lee, *Org. Lett*., 2020, **22**, 3504.
7. C. Tang and N. Jiao, *Angew. Chem. Int. Ed.*, 2014, **53**, 6528.
8. J. Liu, C. Zhang, Z. Zhang, X. Wen, X. Dou, J. Wei, X. Qiu, S. Song and N. Jiao, *Science*, 2020, **367**, 281.
9. H.-L. Wang, W.-F. Jiang and Z.-Q. Li, *J. Chem. Res*., 2009, **8**, 508.
10. S. Brammer, U. Lüning and C. Kühl, *Eur. J. Org. Chem*., 2002, **23**, 4054.
11. A. Fawcett, M. J. Keller, Z. Herrera and J. F. Hartwig, *Angew. Chem. Int. Ed*., 2021, **60**, 8276.
12. W. Huang, Y. Wang, Y. Weng, M. Shrestha, J. Qu and Y. Chen, *Org. Lett*., 2020, **22**, 3245.
13. T. Liu, W. Jia, Q. Xi, Y. Chen, X. Wang and D. Yin, *J. Org. Chem*., 2018, **83**, 1387.
14. Z. Li, X. Li, X. Ni and J.-P. Cheng, *Org. Lett.*, 2015, **17**, 1196.
15. X. Qi, H.-J. Ai, C.-X. Cai, J.-B. Peng, J. Ying and X.-F. Wu, *Eur. J. Org. Chem.*, 2017, **48**, 7222.
16. A.-M. Schoepf, S. Salcher, P. Obexer and R. Gust, *Eur. J. Med. Chem.*, 2020, **195**, 112258.

^{1}H NMR and ^{13}C NMR spectra for the products

2a: benzamide

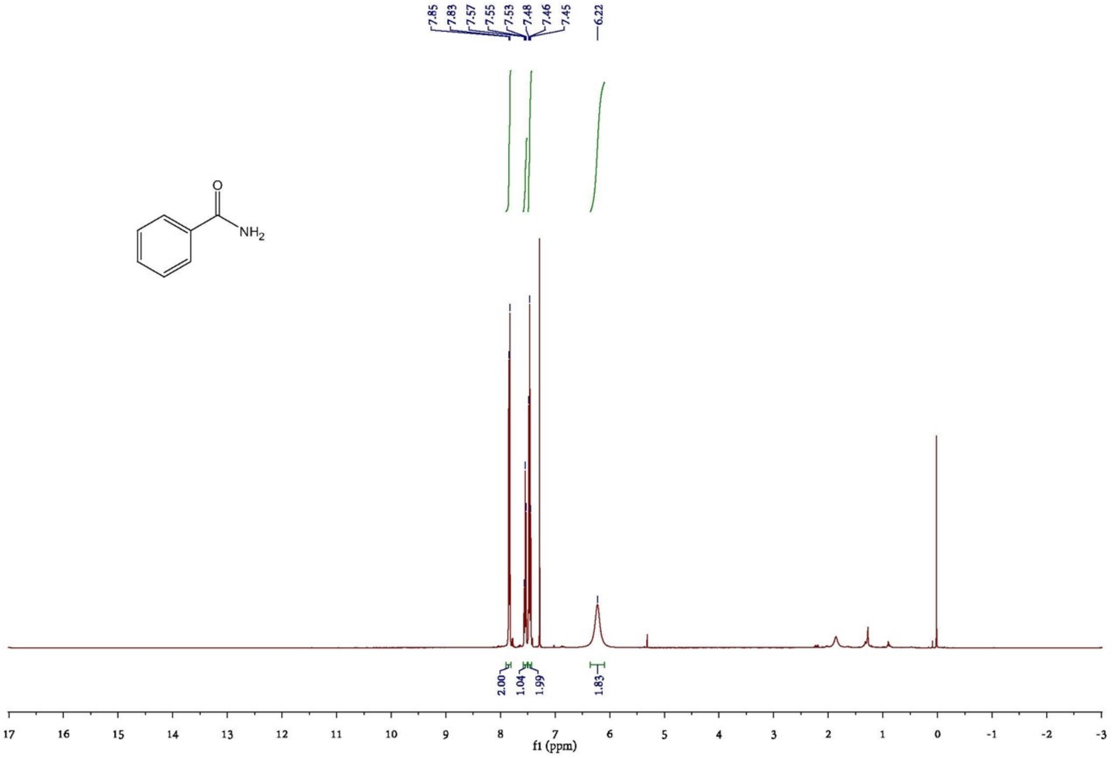

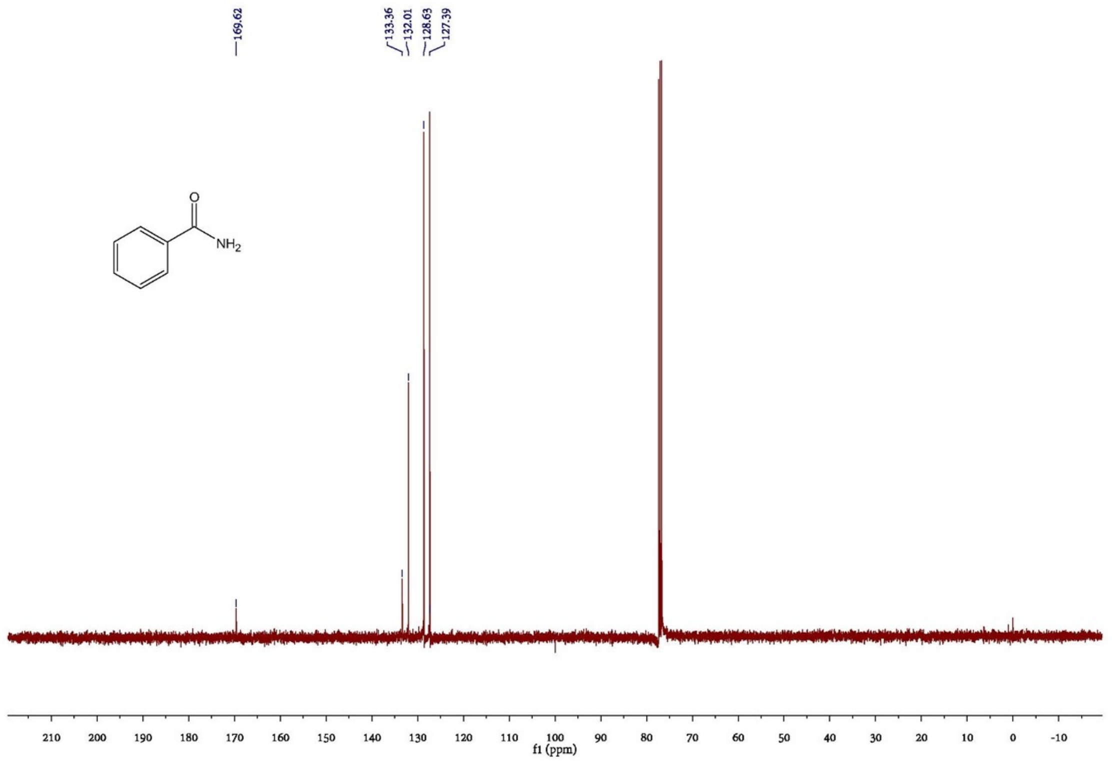

2b: 2-aminobenzamide

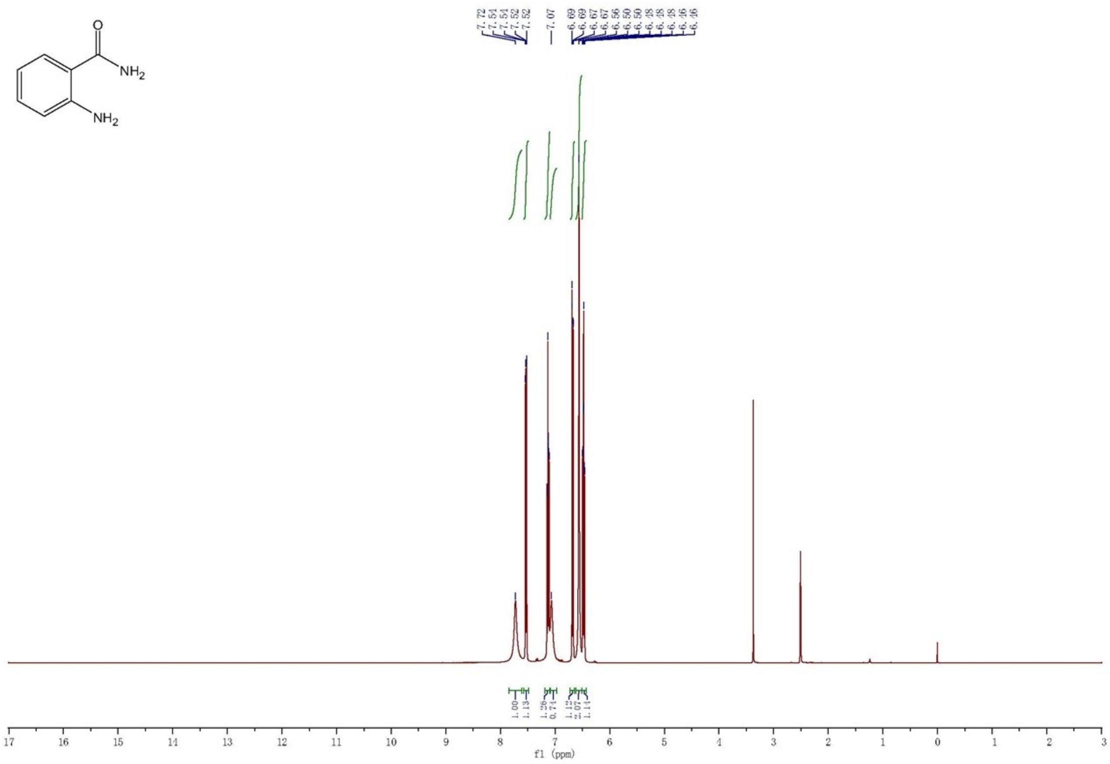

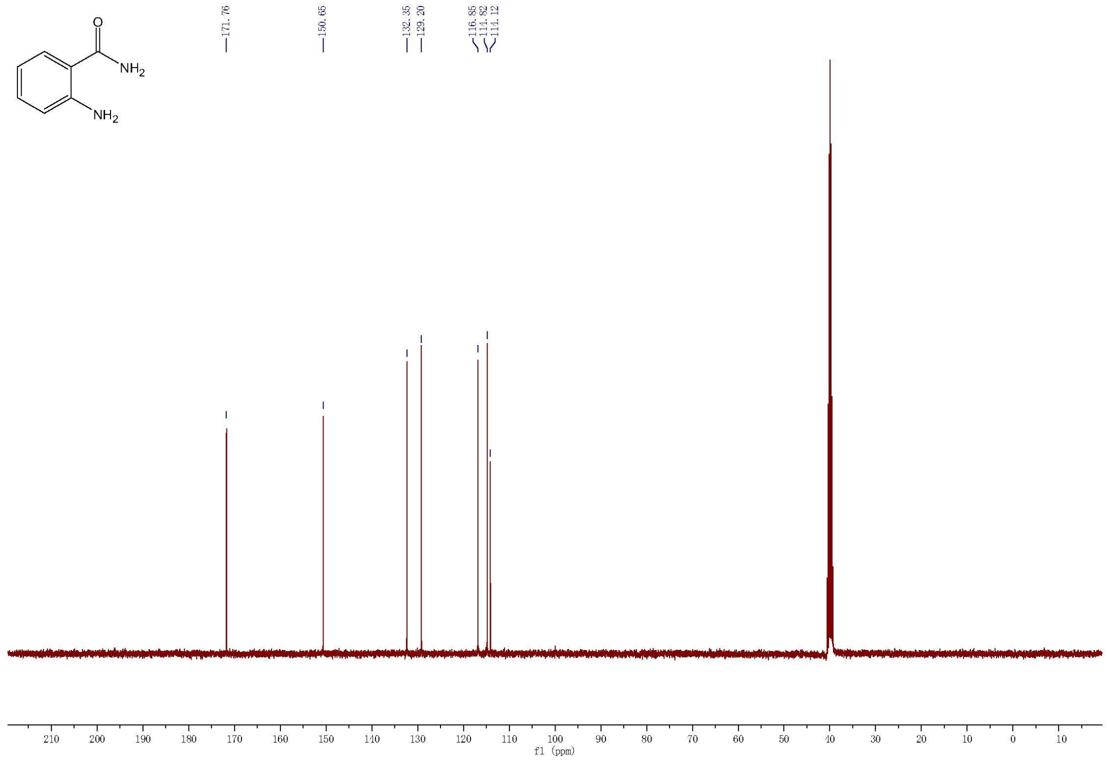

2c: 2-methoxybenzamide

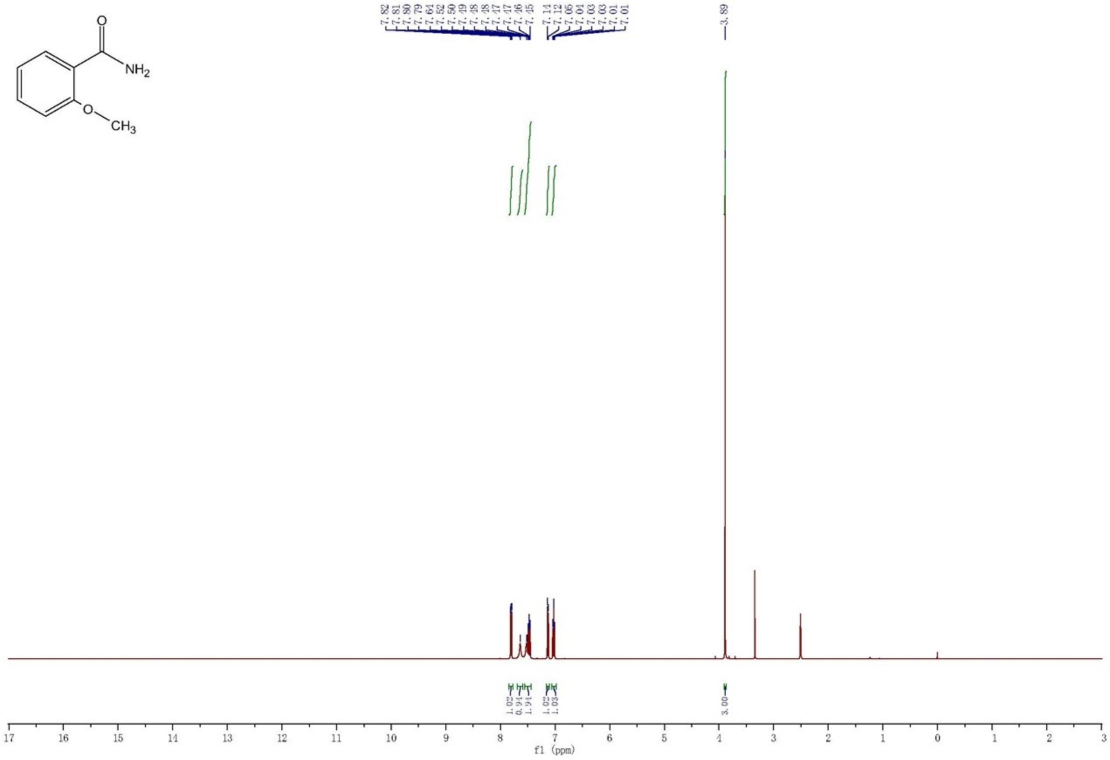

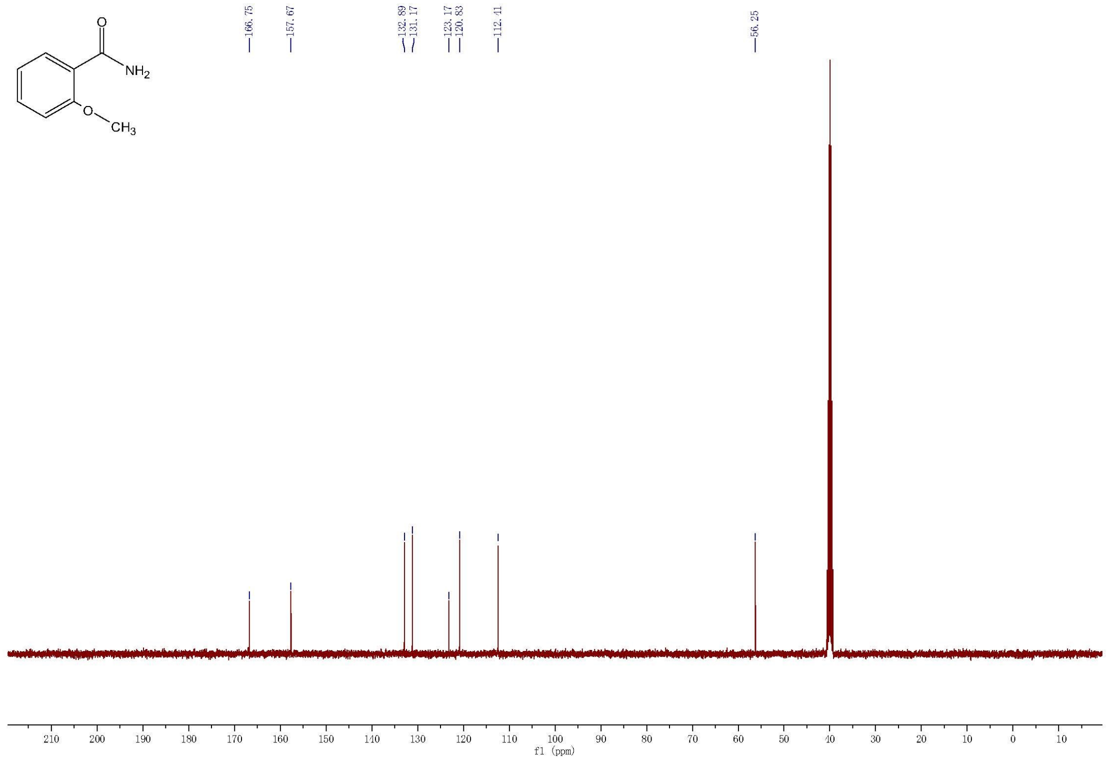

2d: 2-bromobenzamide

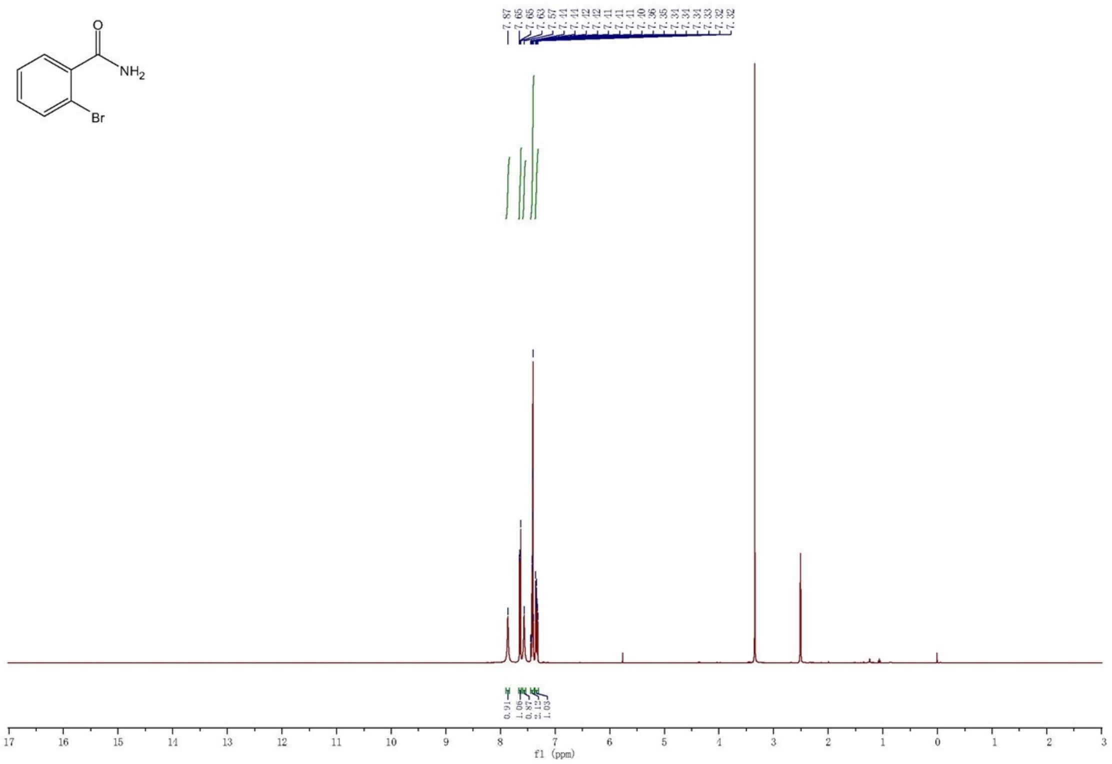

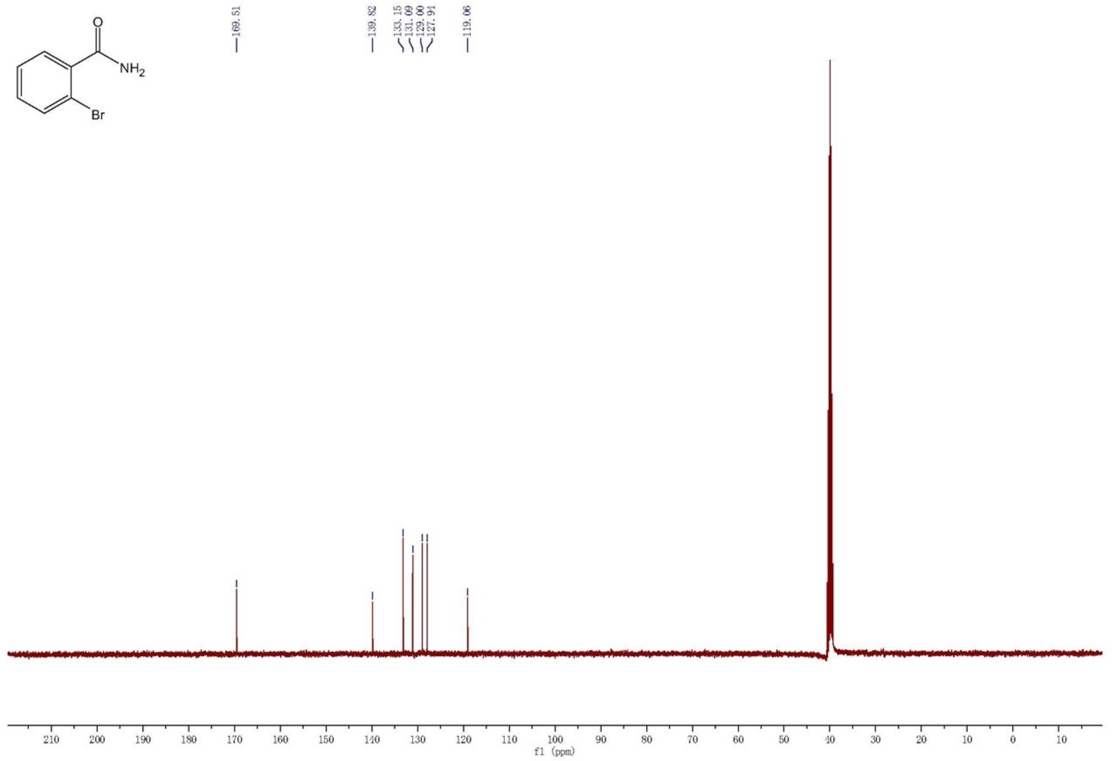

2e: 2-chlorobenzamide

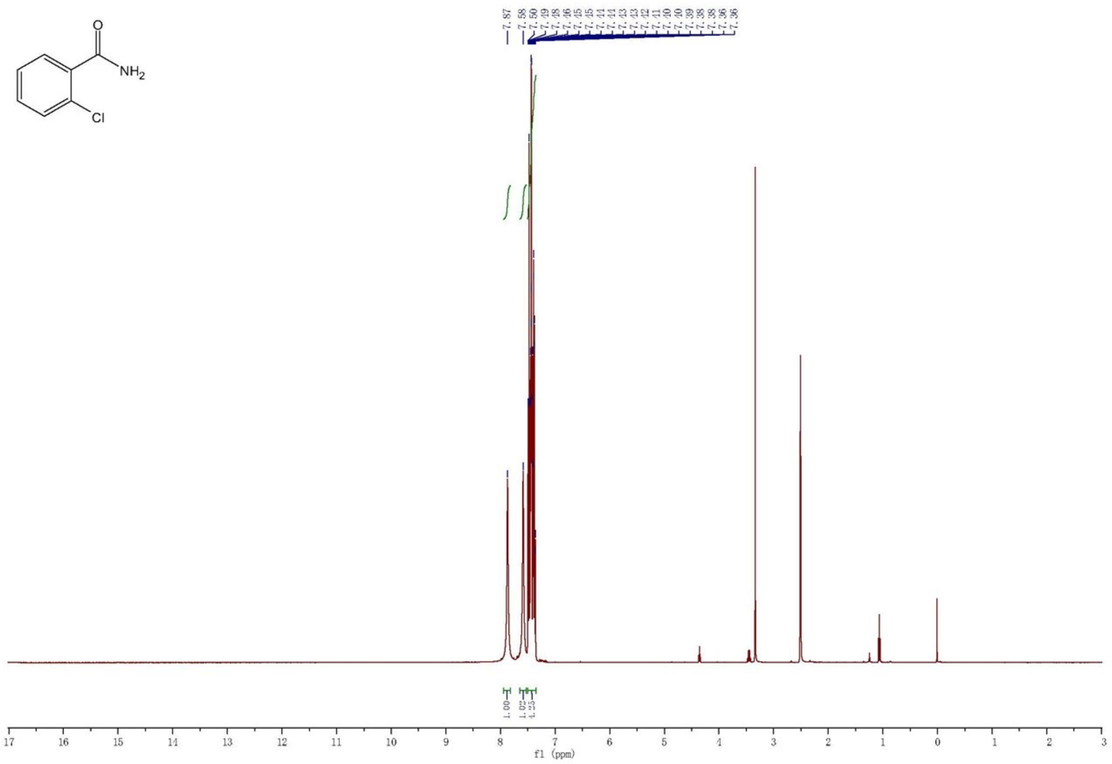

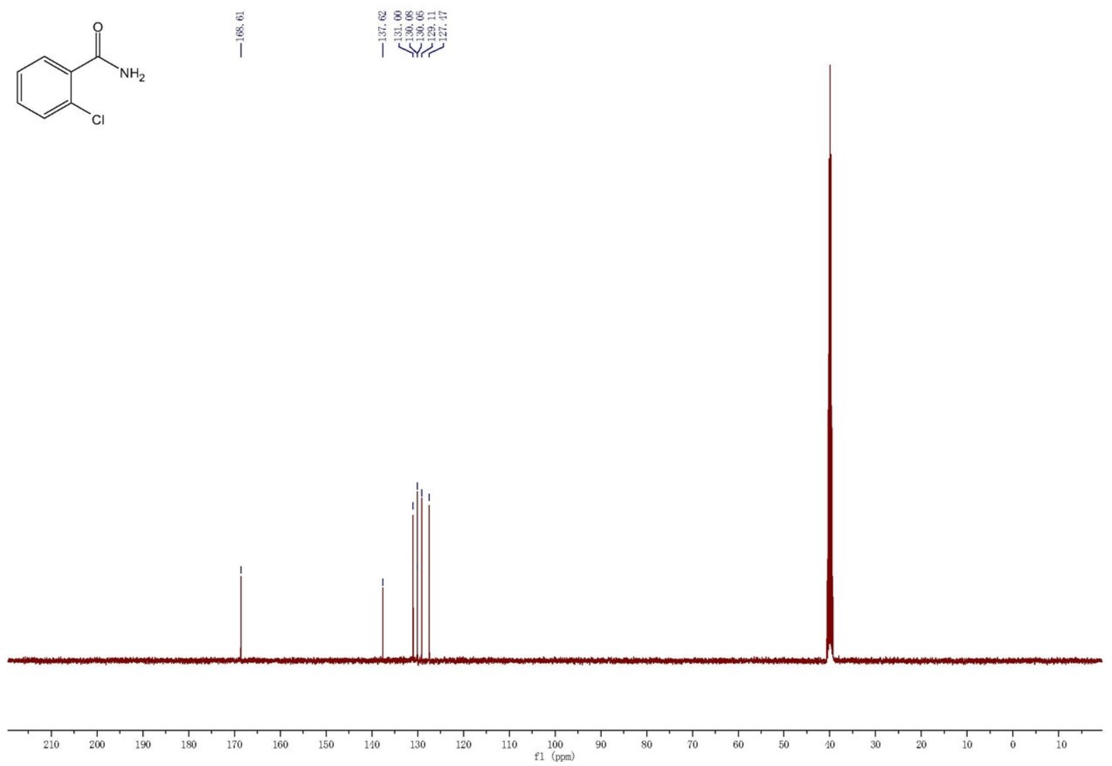

2f: 2-iodobenzamide

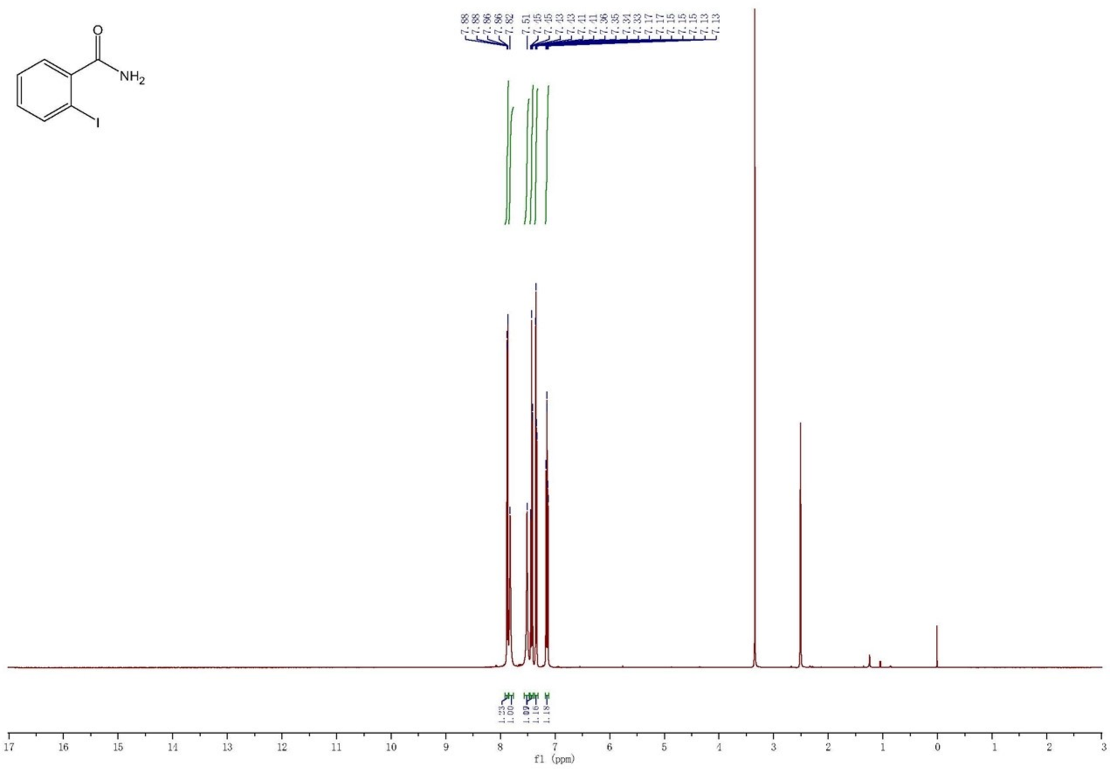

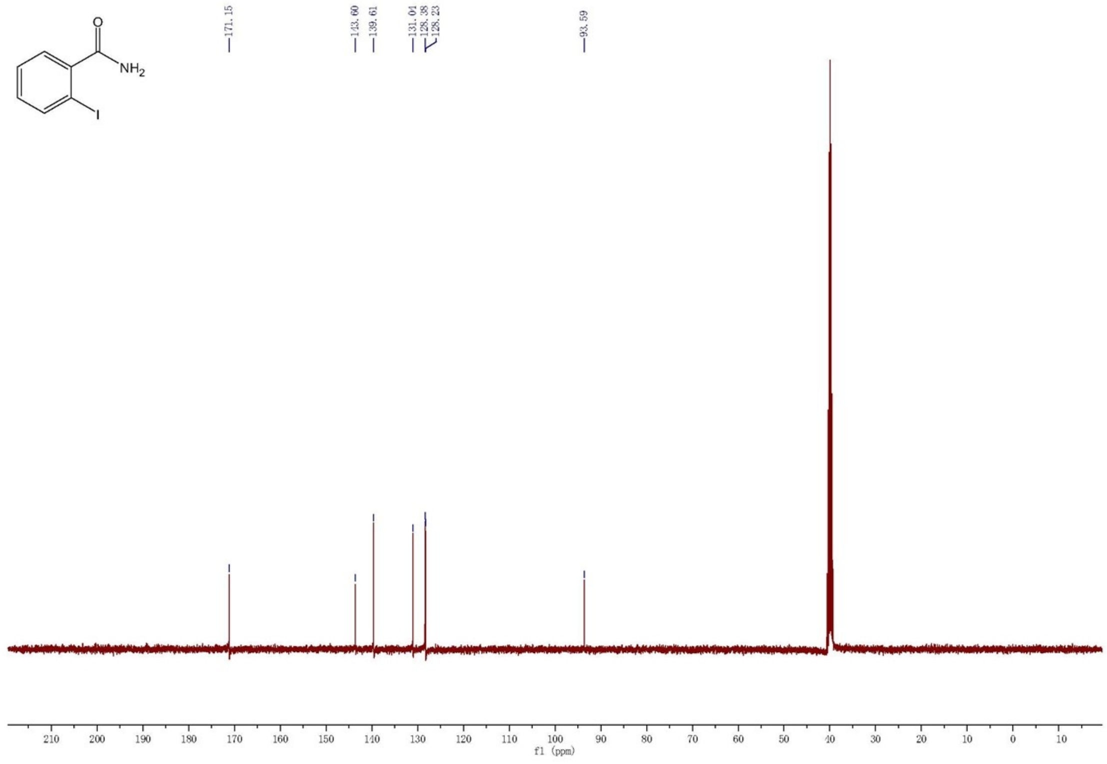

2g: 4-aminobenzamide

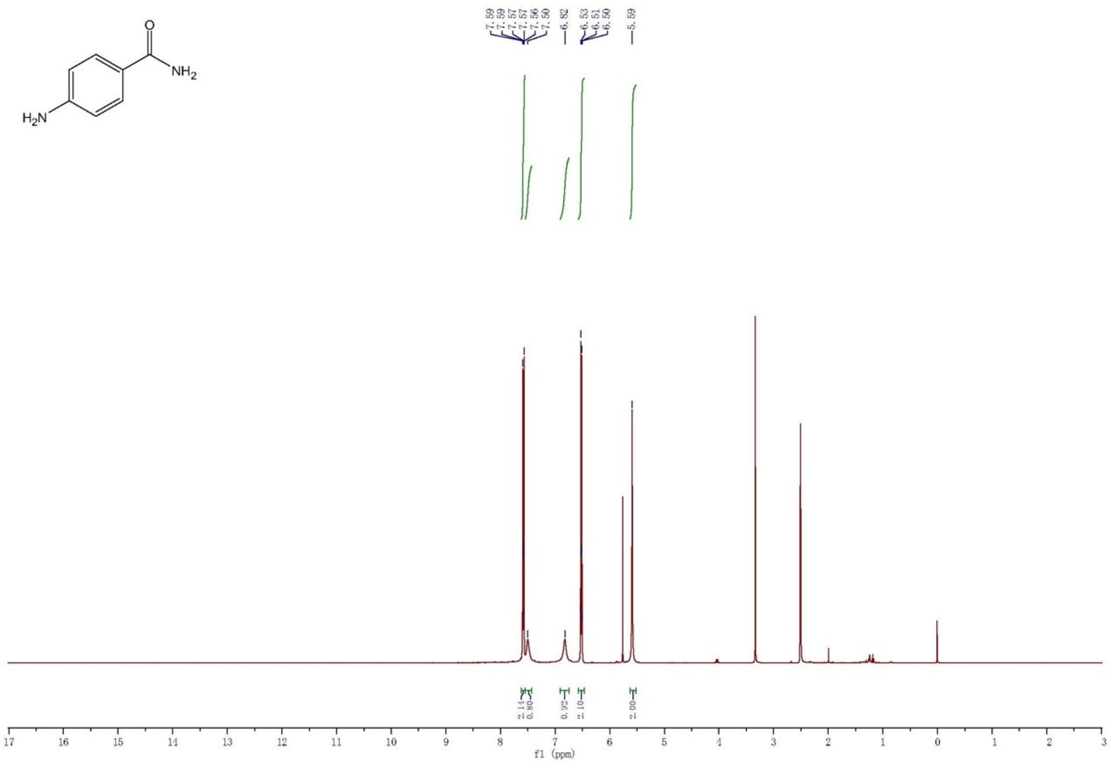

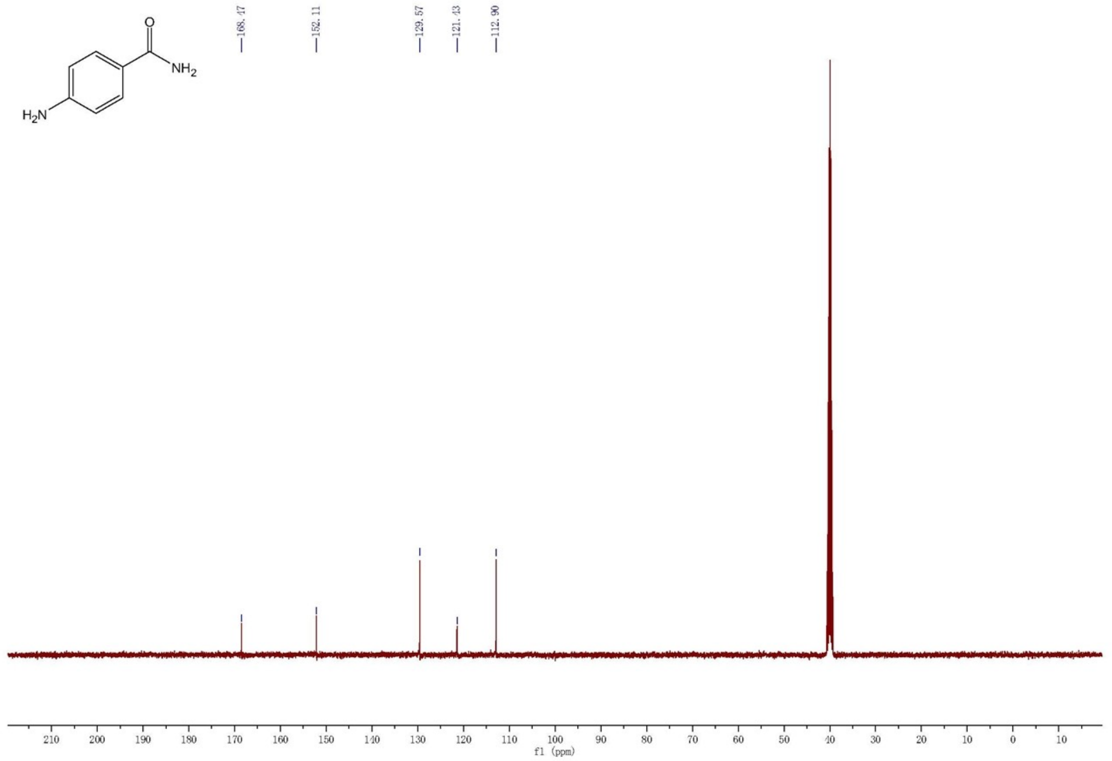

2h: 2-nitrobenzamide

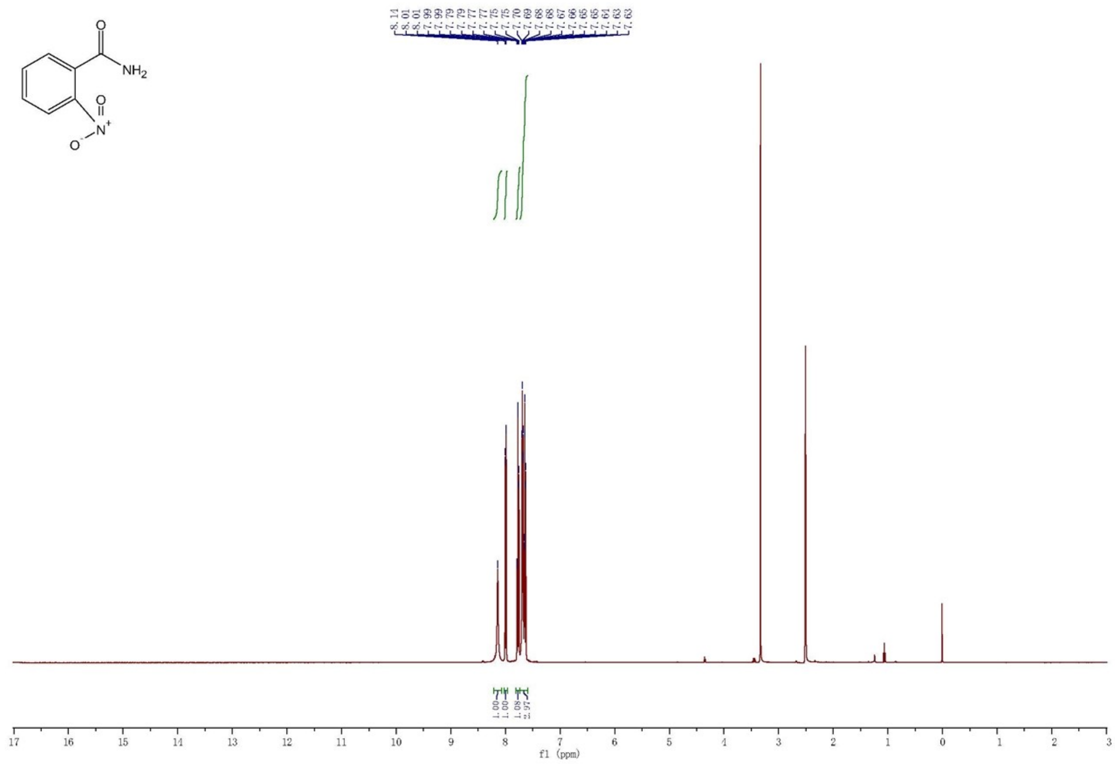

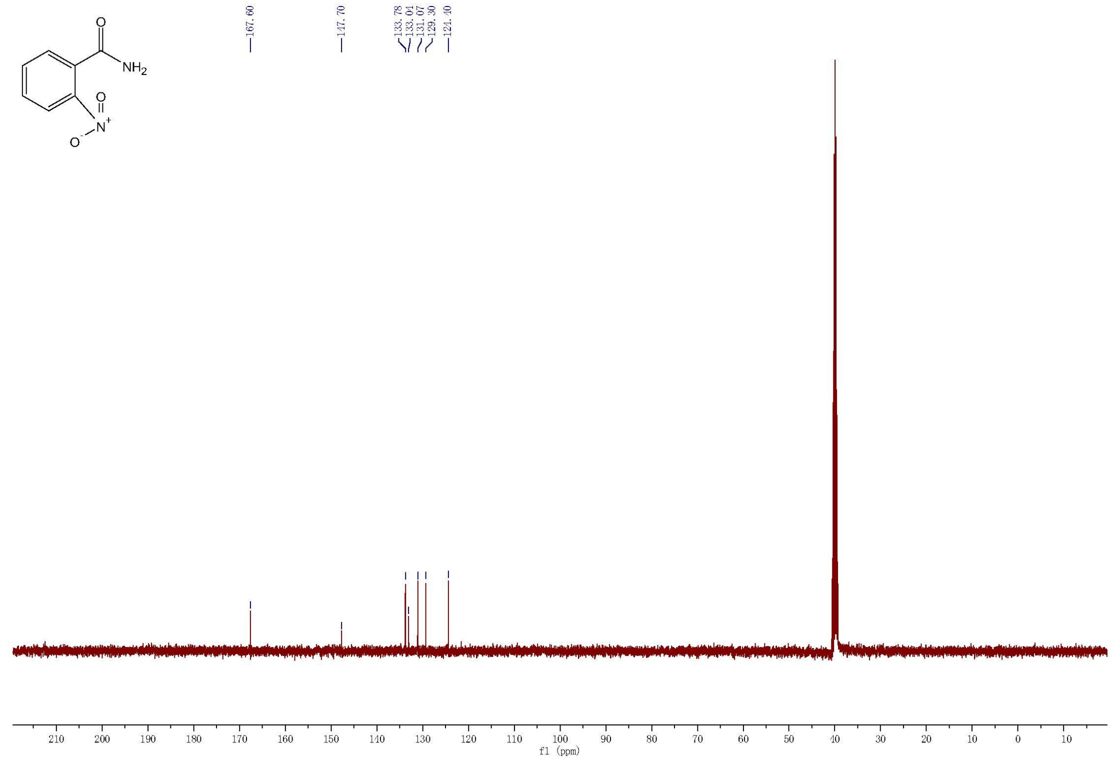

2i: 2-iodo-5-methylbenzamide

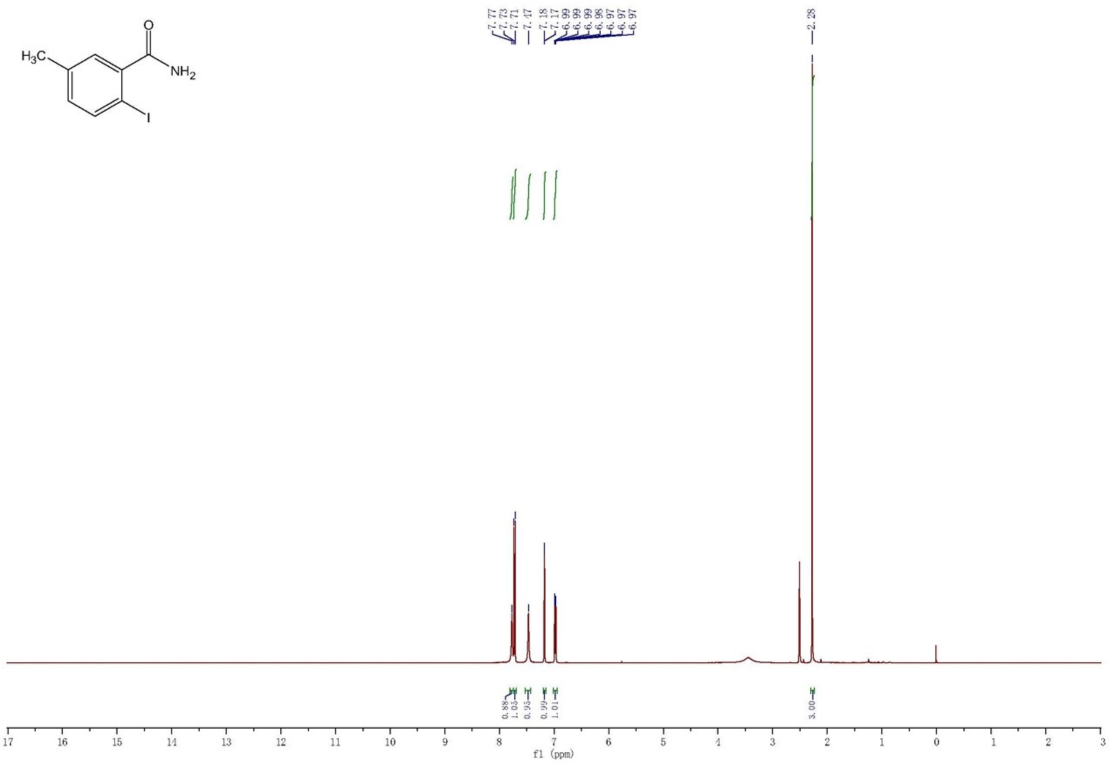

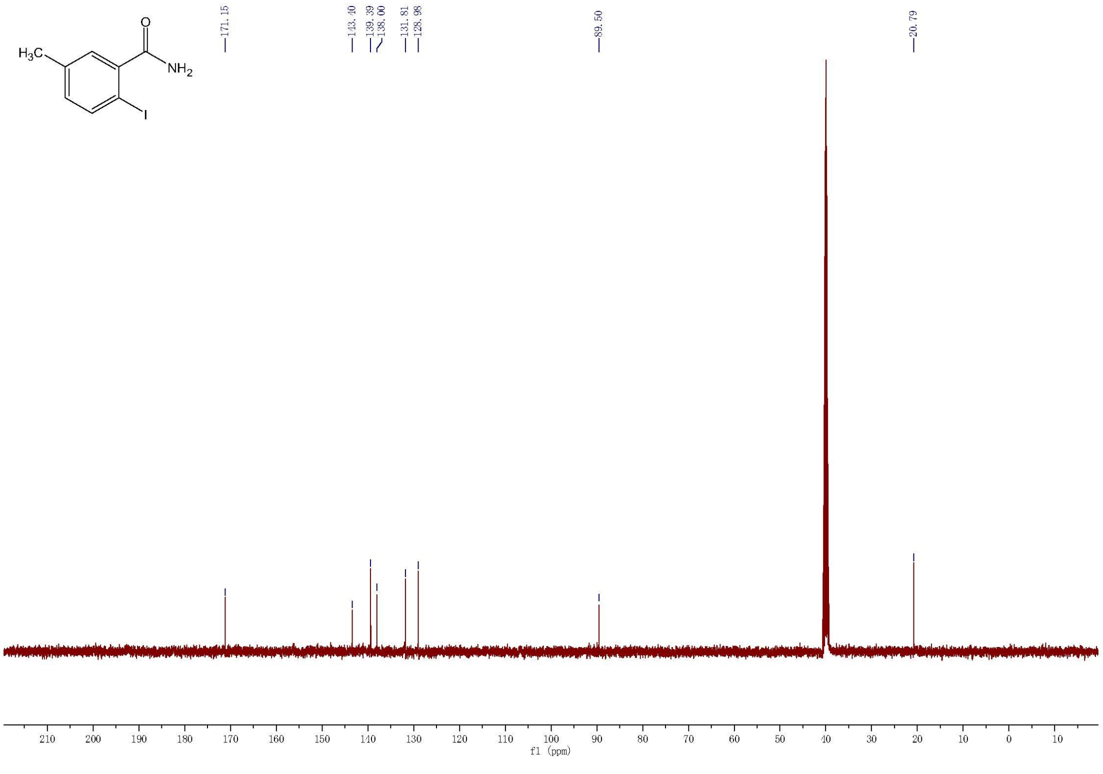

2j: 3-aminobenzamide

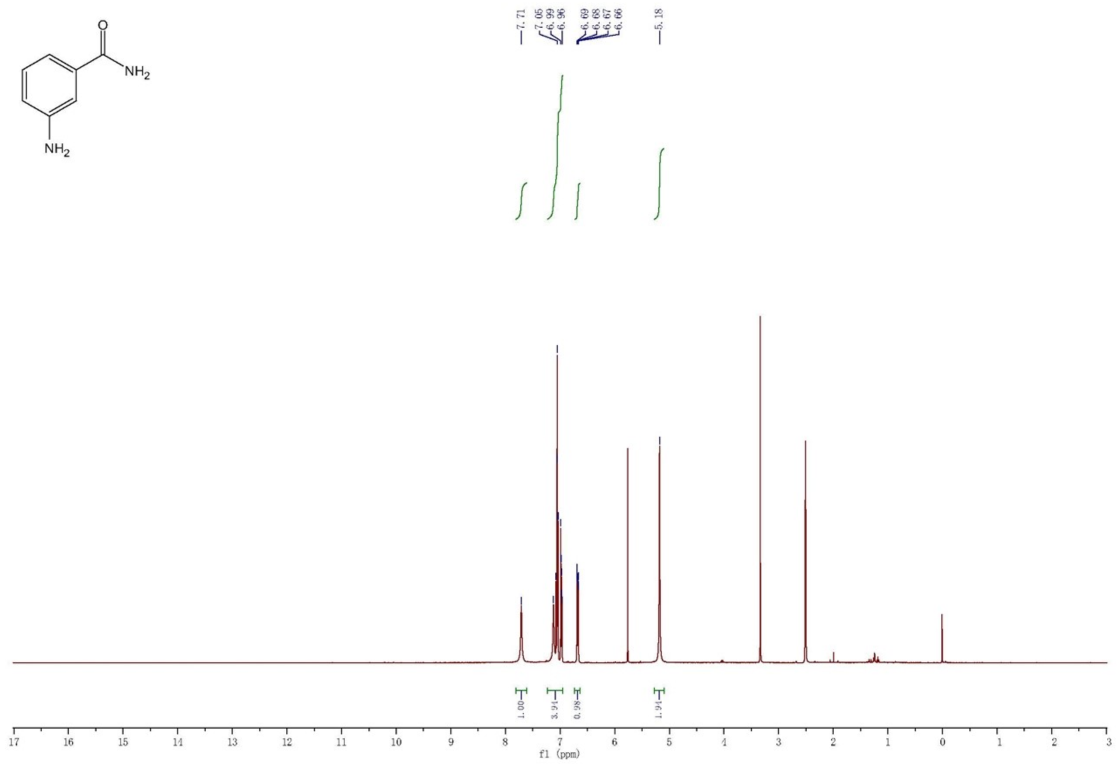

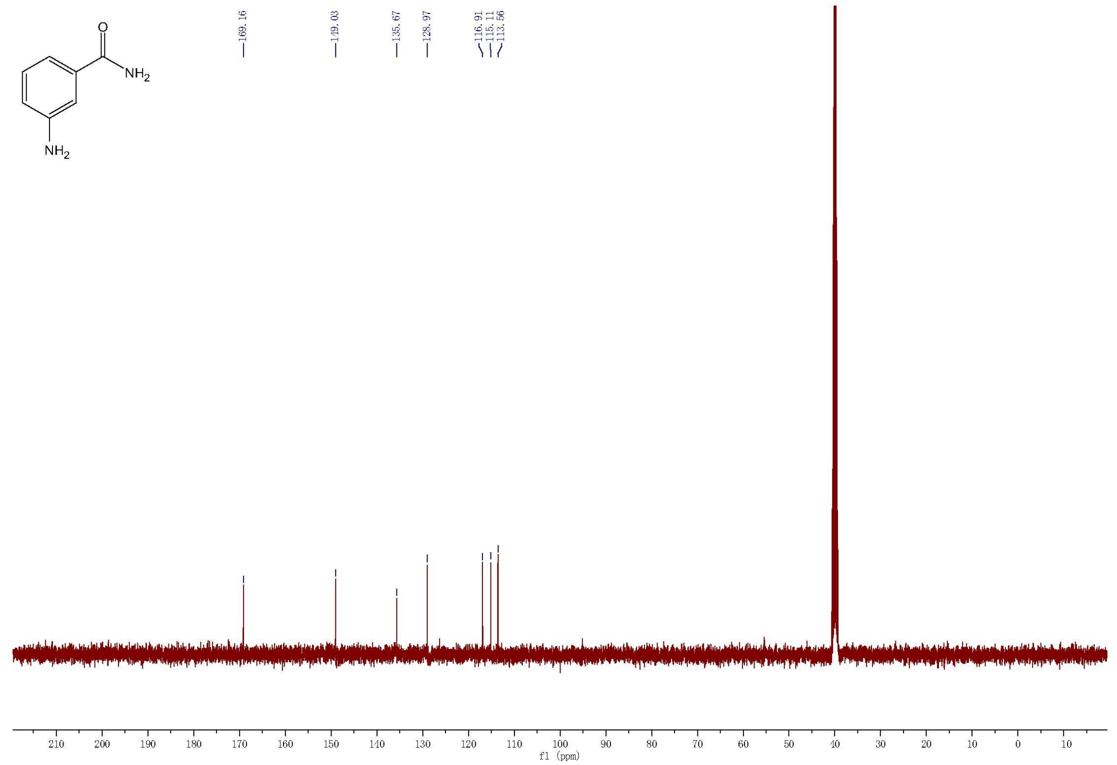

2k: 3-methylbenzamide

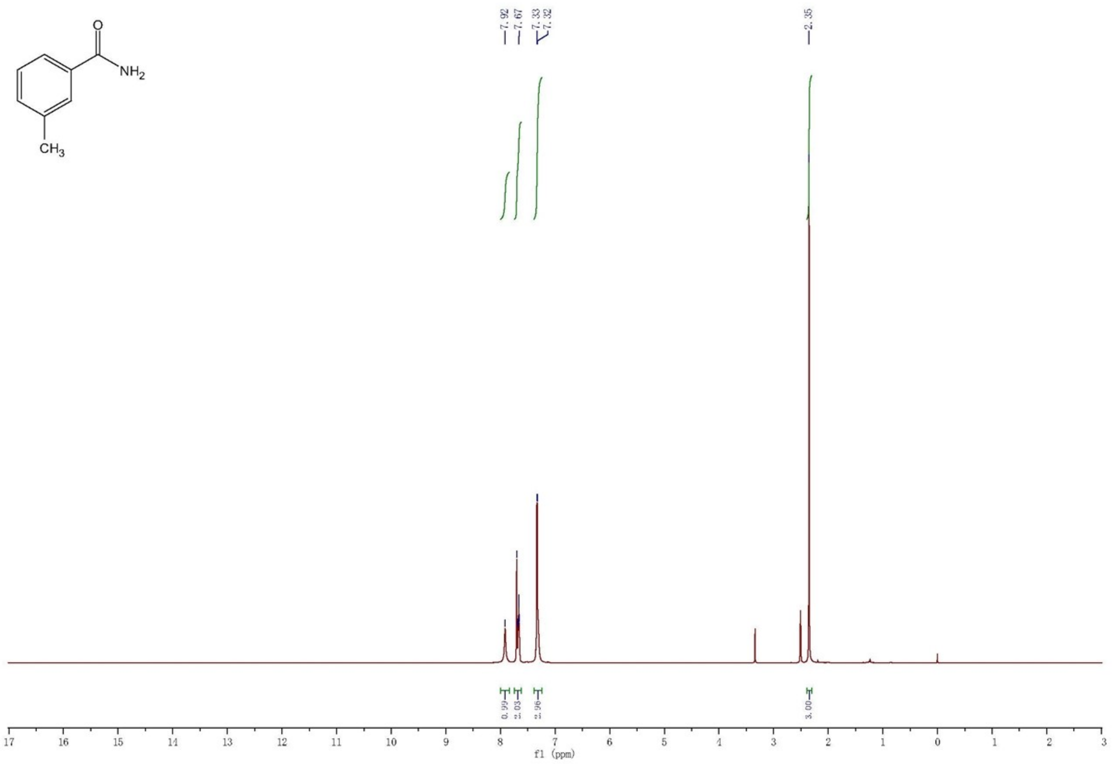

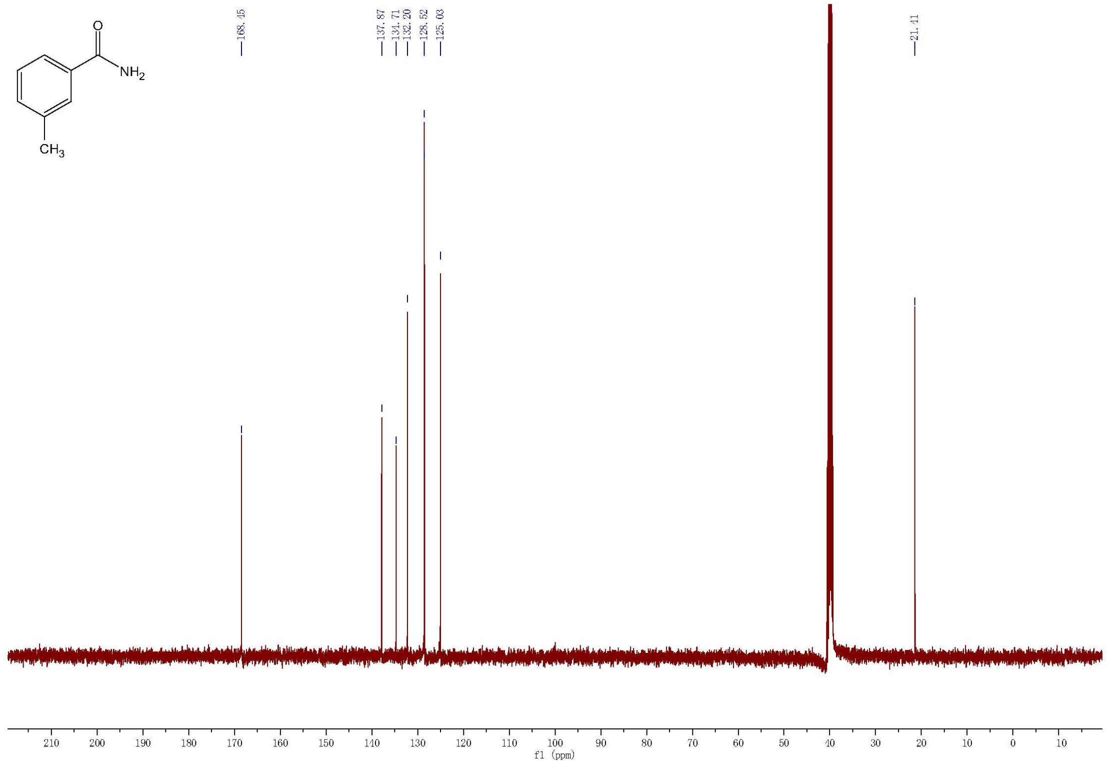

2l: 3-fluorobenzamide

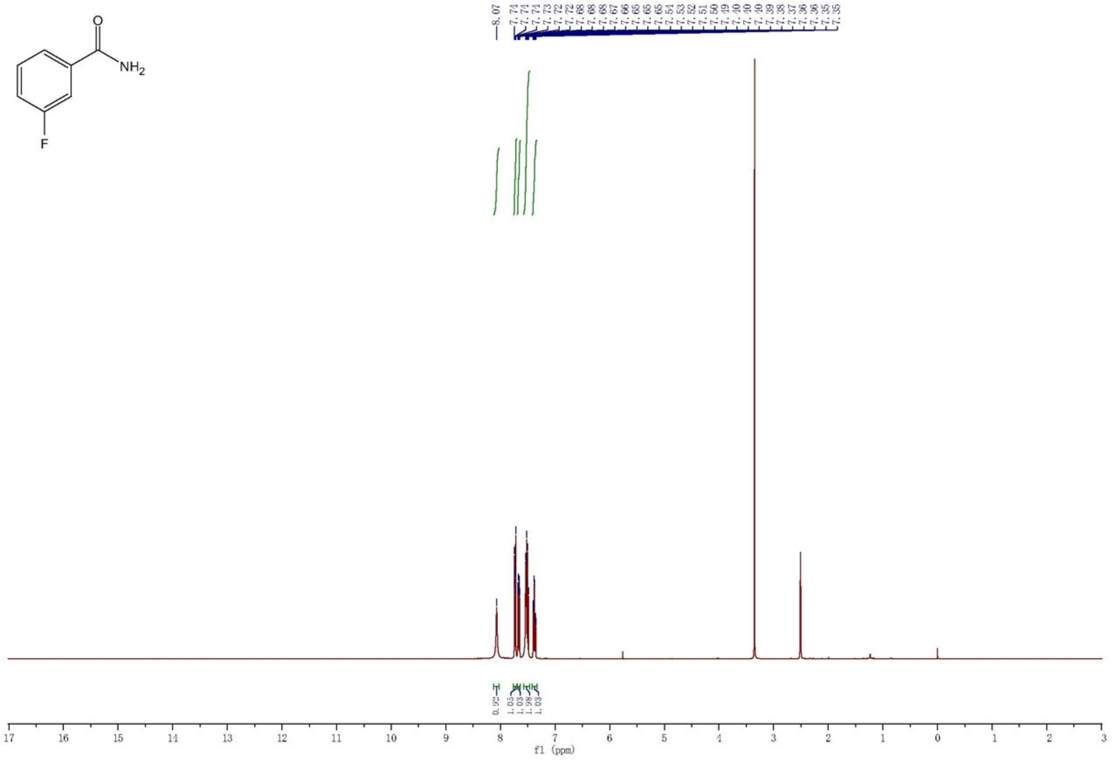

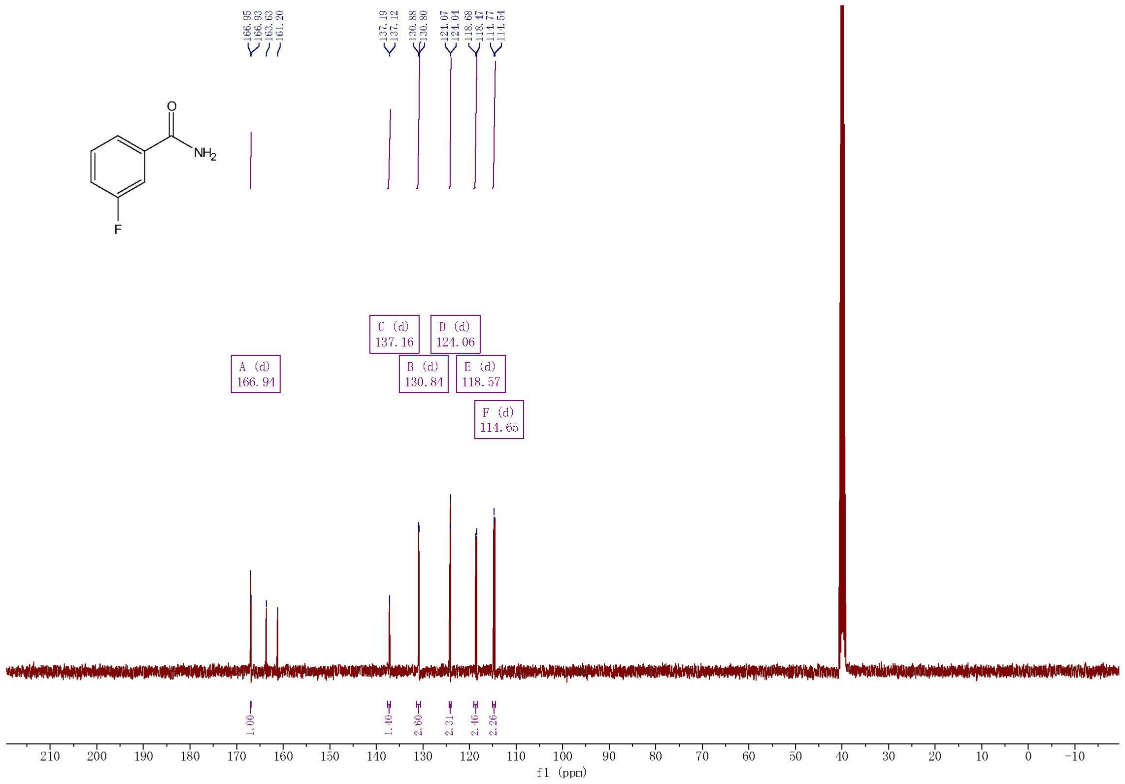

2m: 4-methylbenzamide

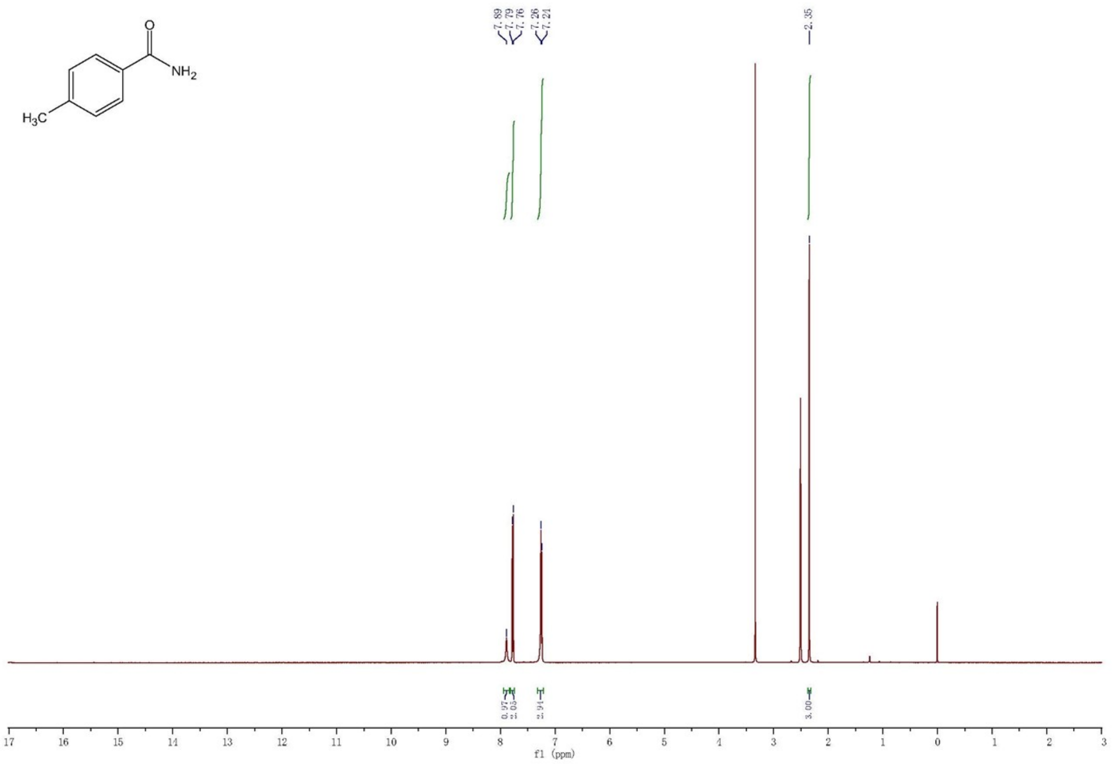

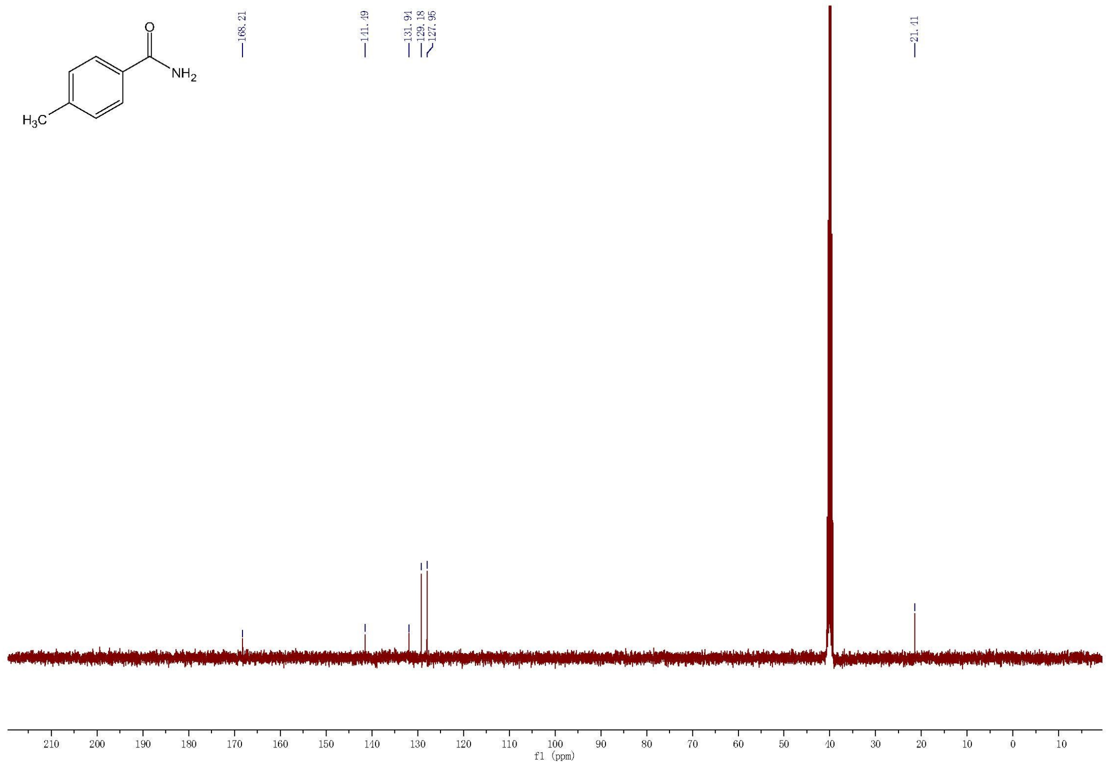

2n: 4-methoxybenzamide

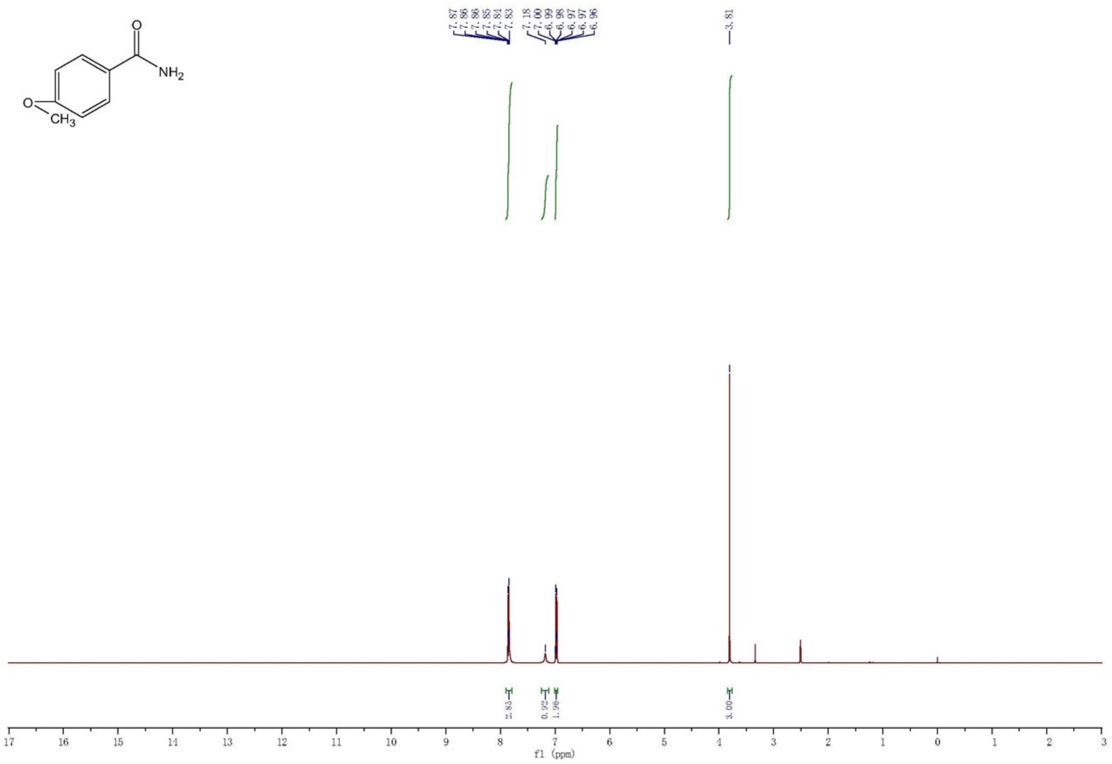

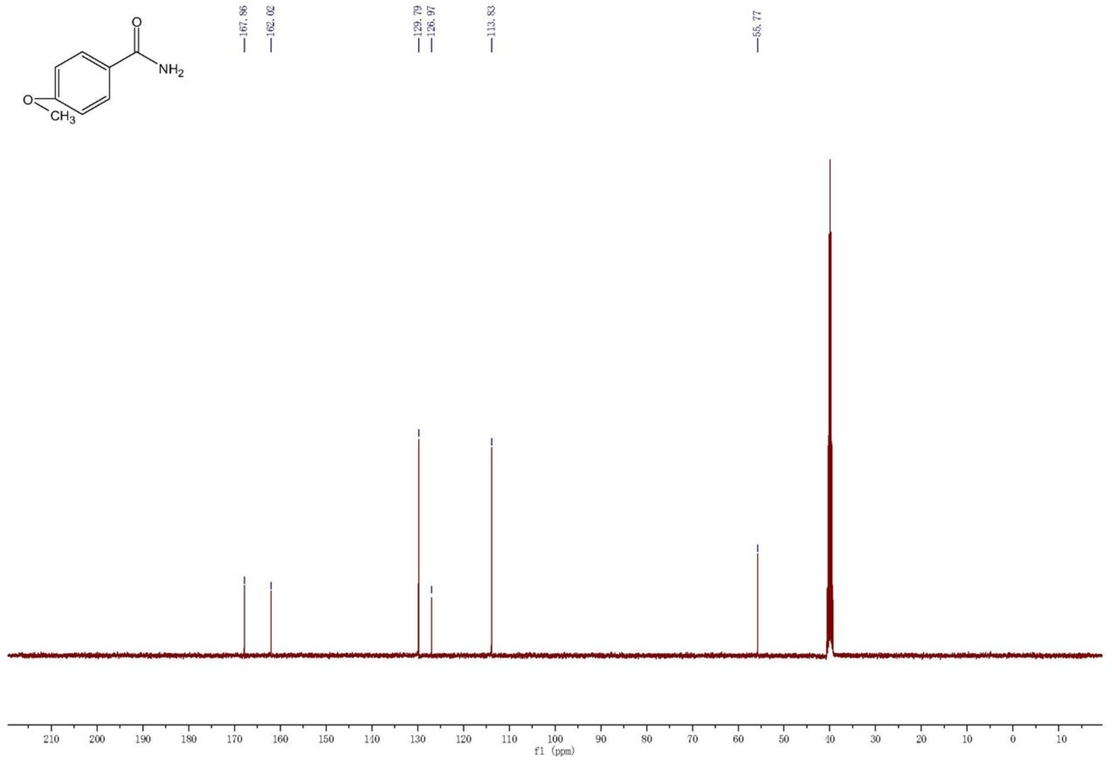

2o: 4-chlorobenzamide

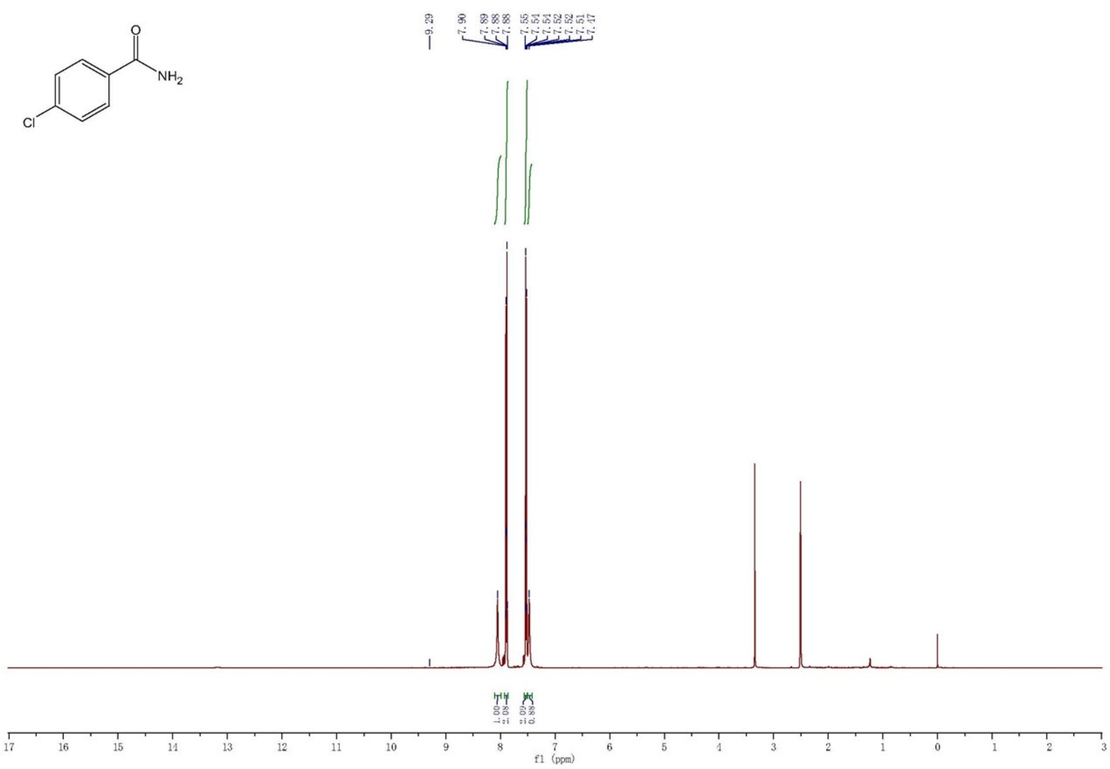

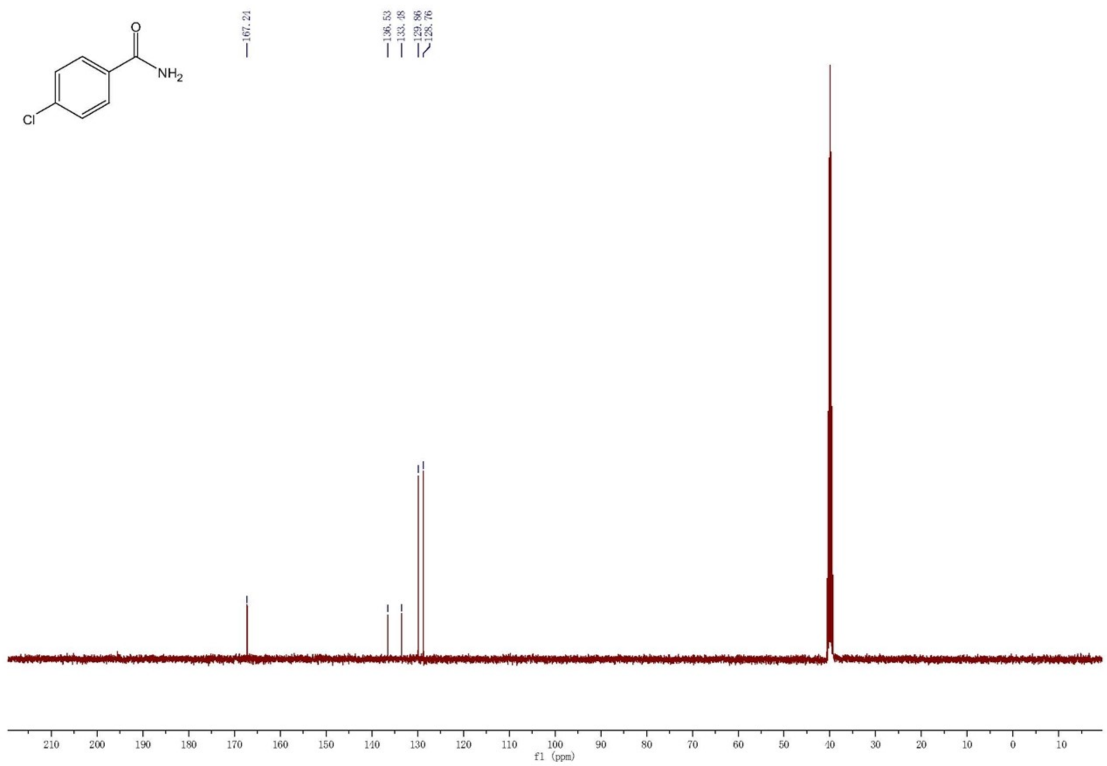

2p: 4-acetylbenzamide

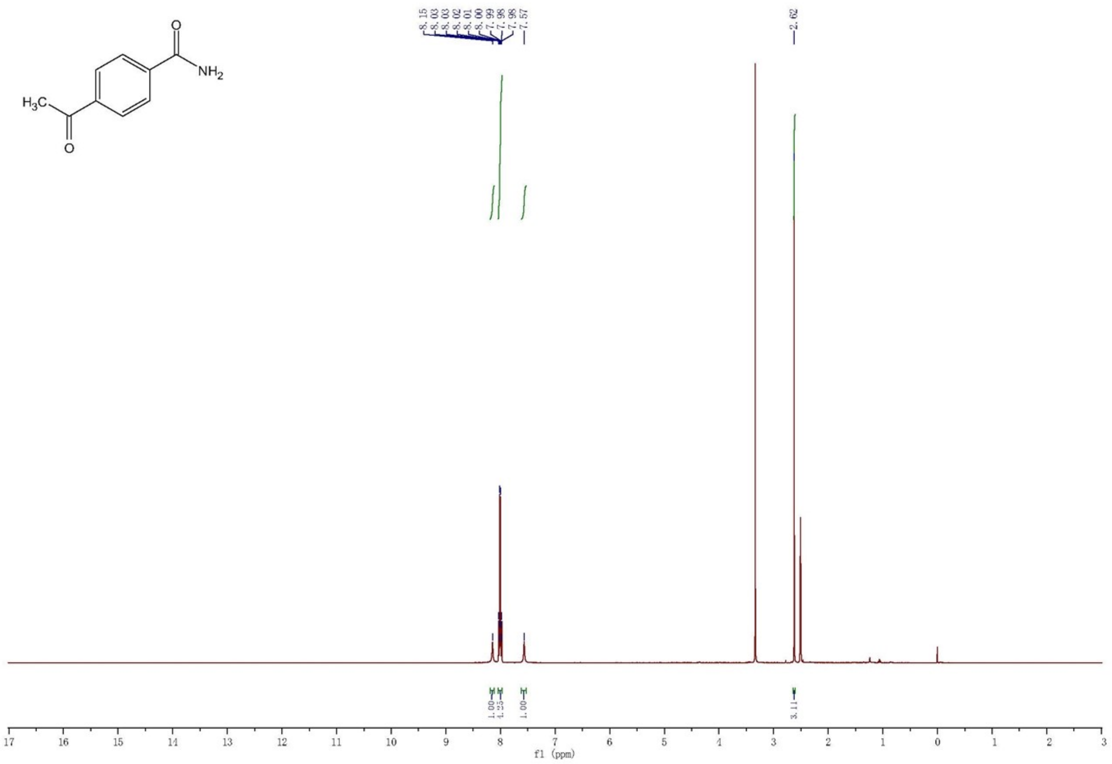

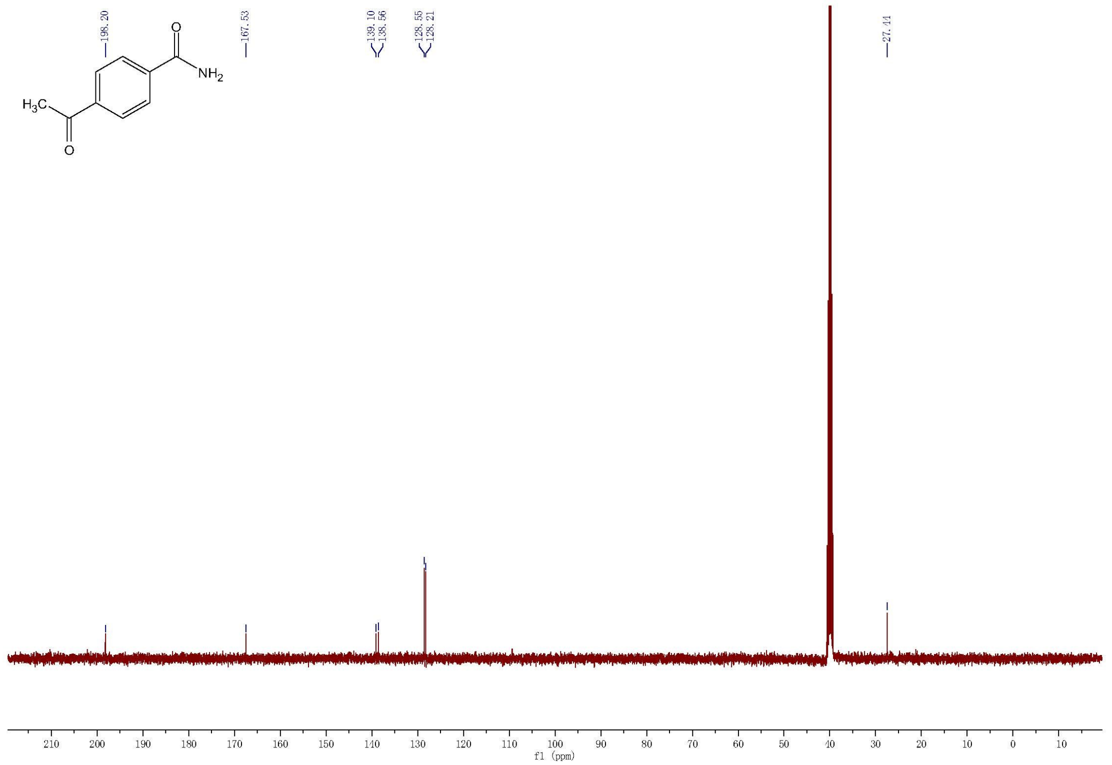

2q: 4-(dimethylamino)benzamide

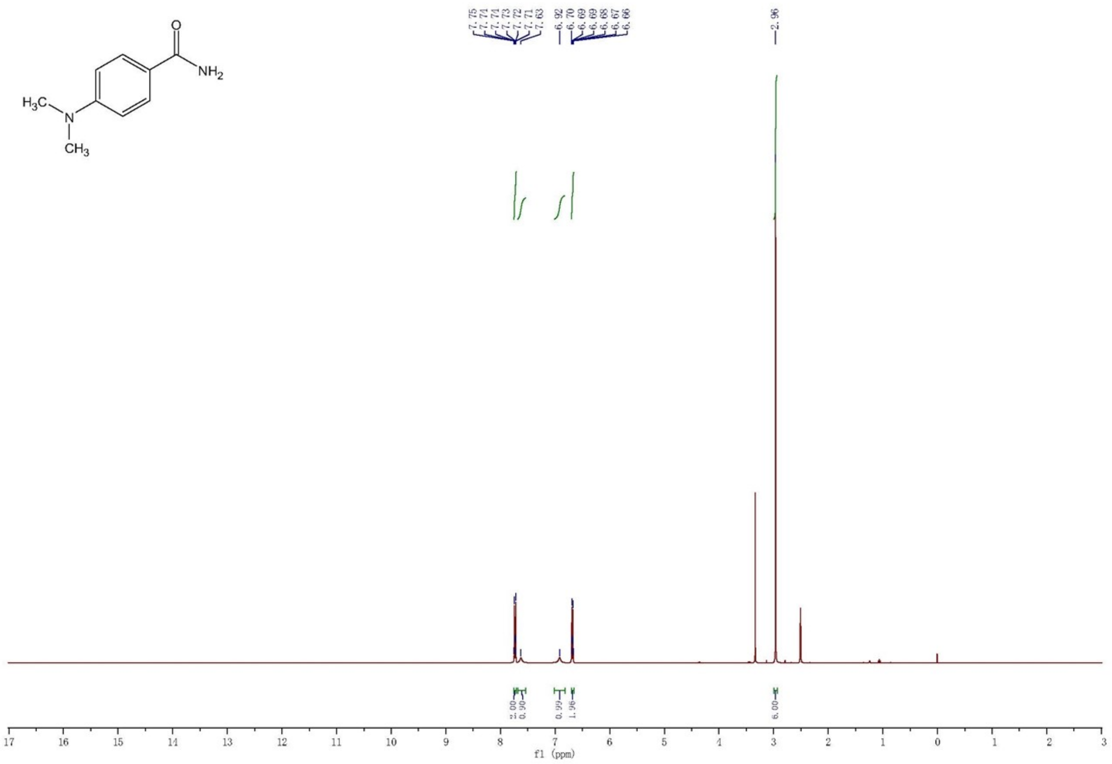

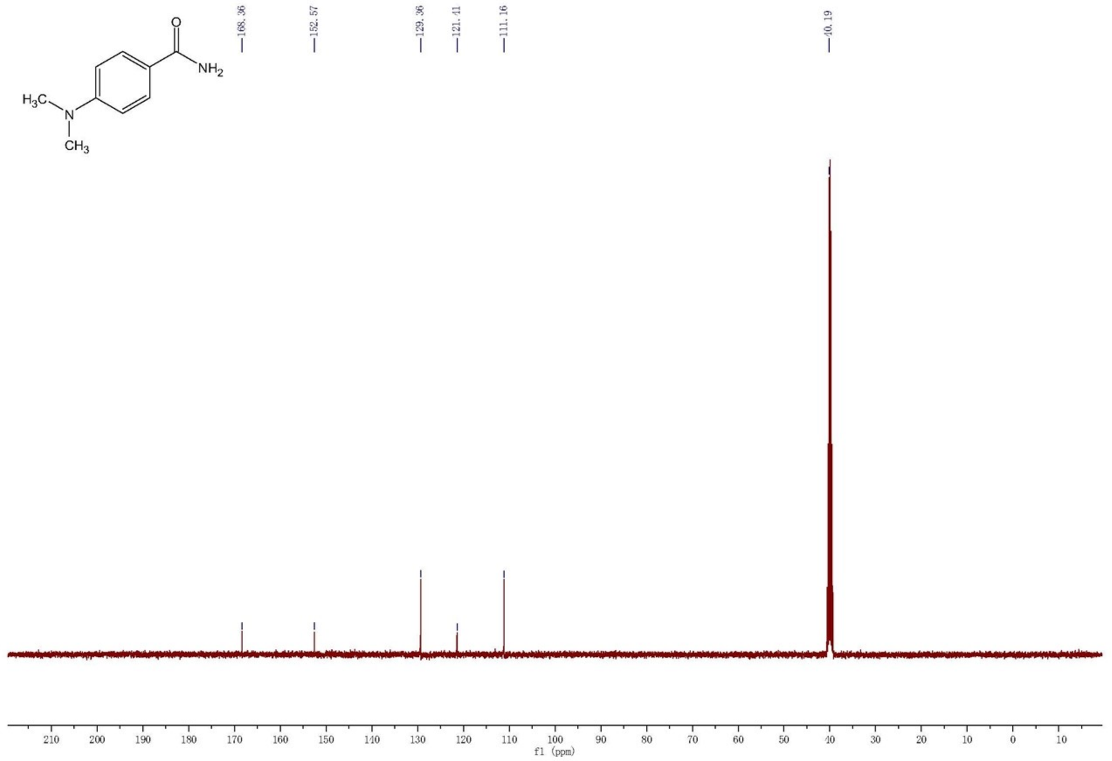

2r: 4-phenyl benzamide

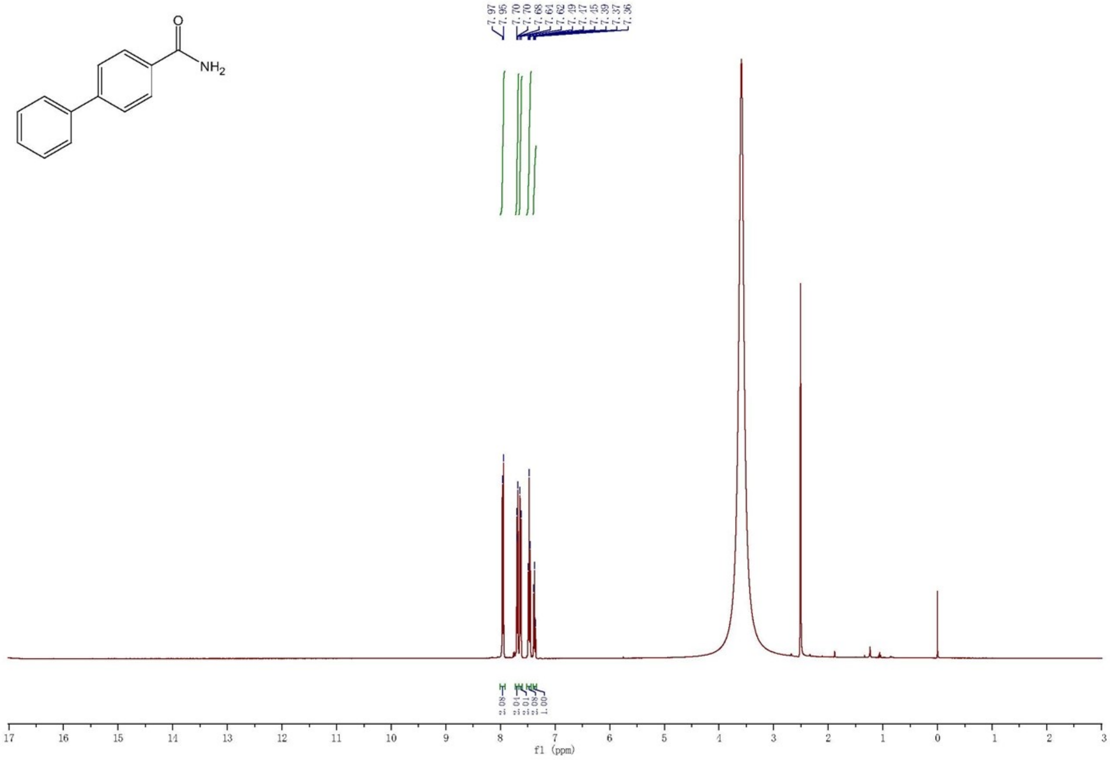

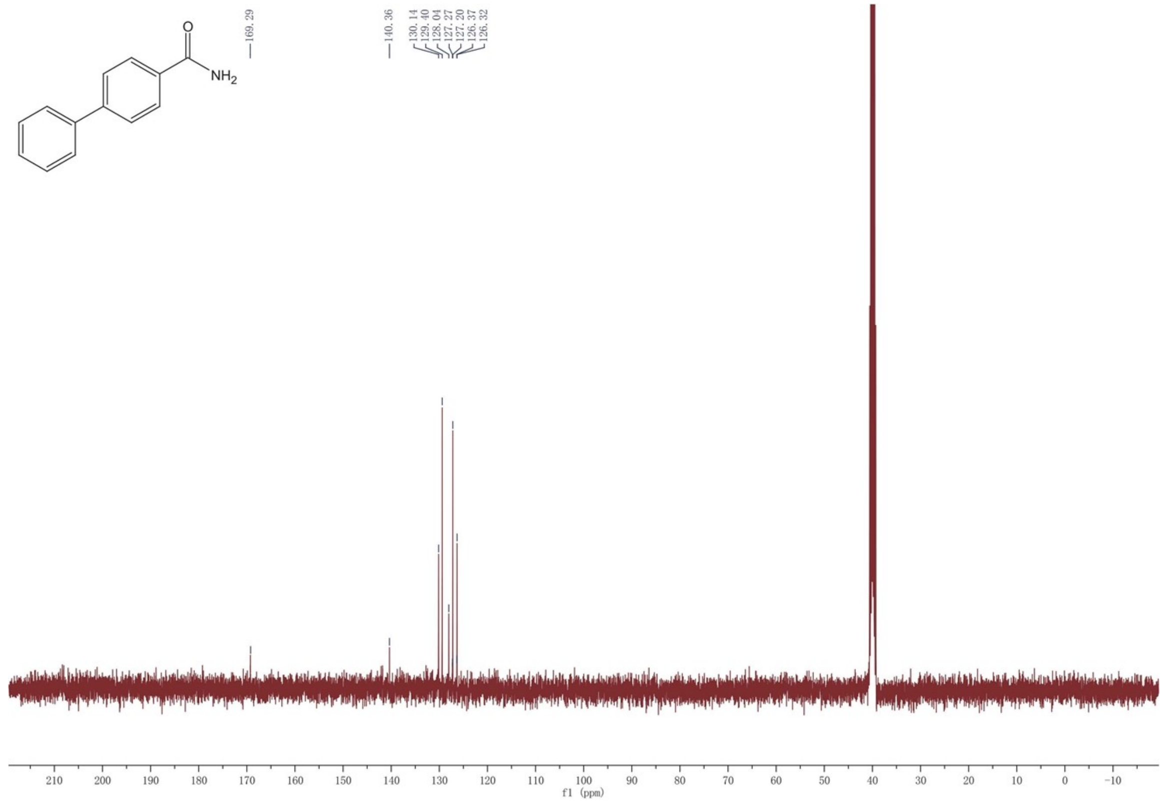

2s: 4-nitrobenzamide

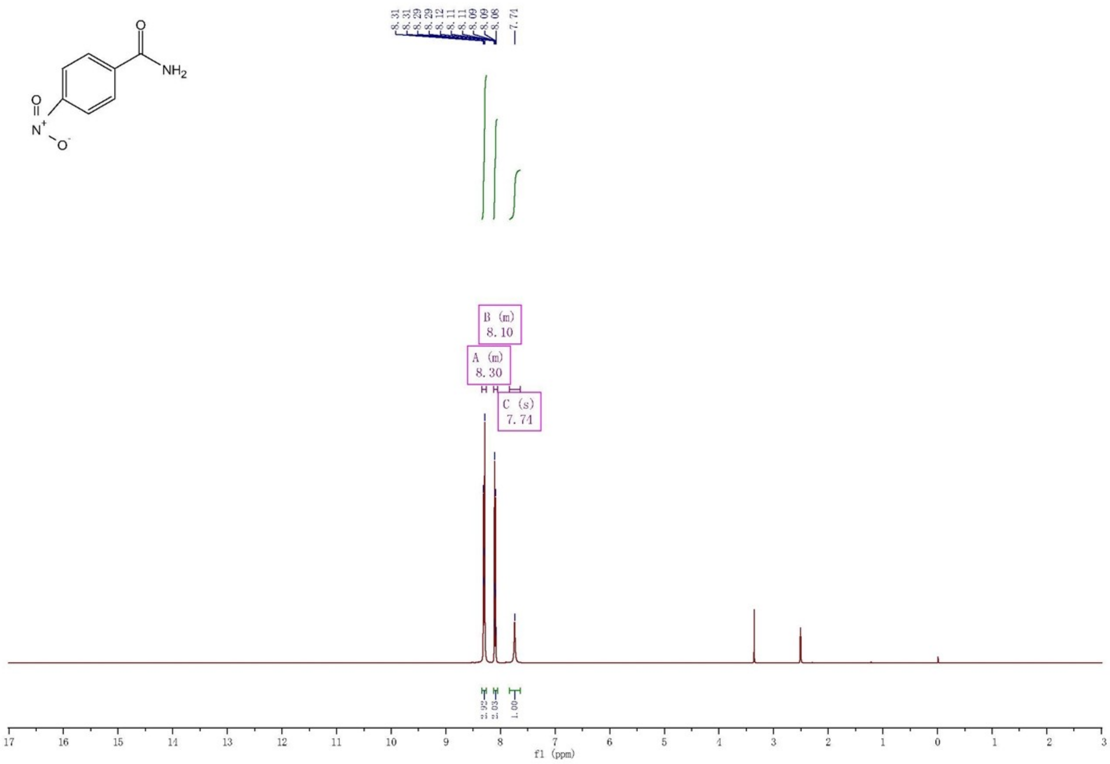

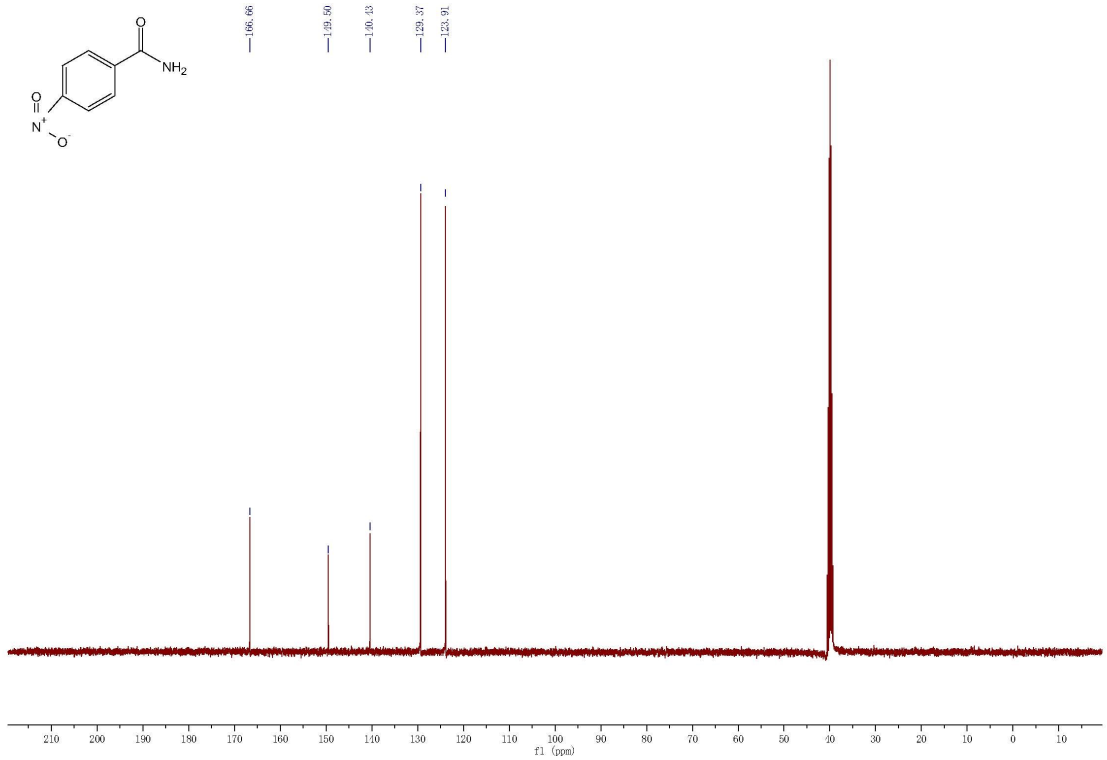

2t: 4-(trifluoromethyl)benzamide

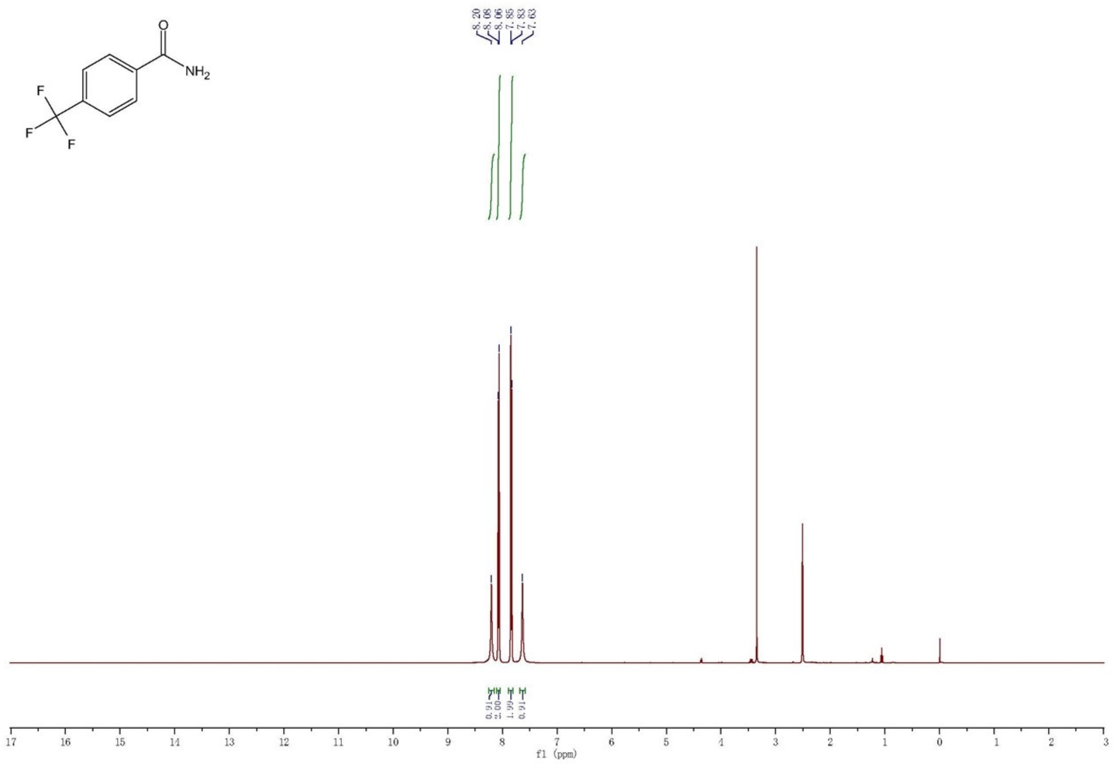

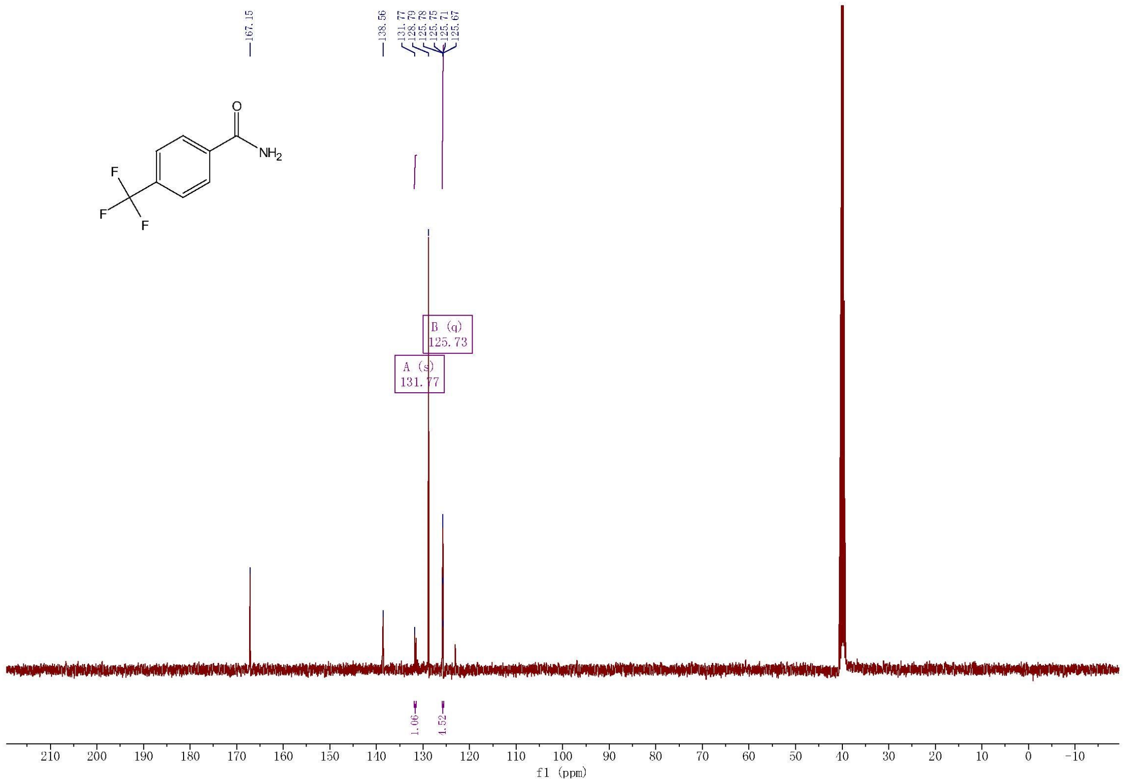

2u: 3,4,5-trimethoxybenzamide

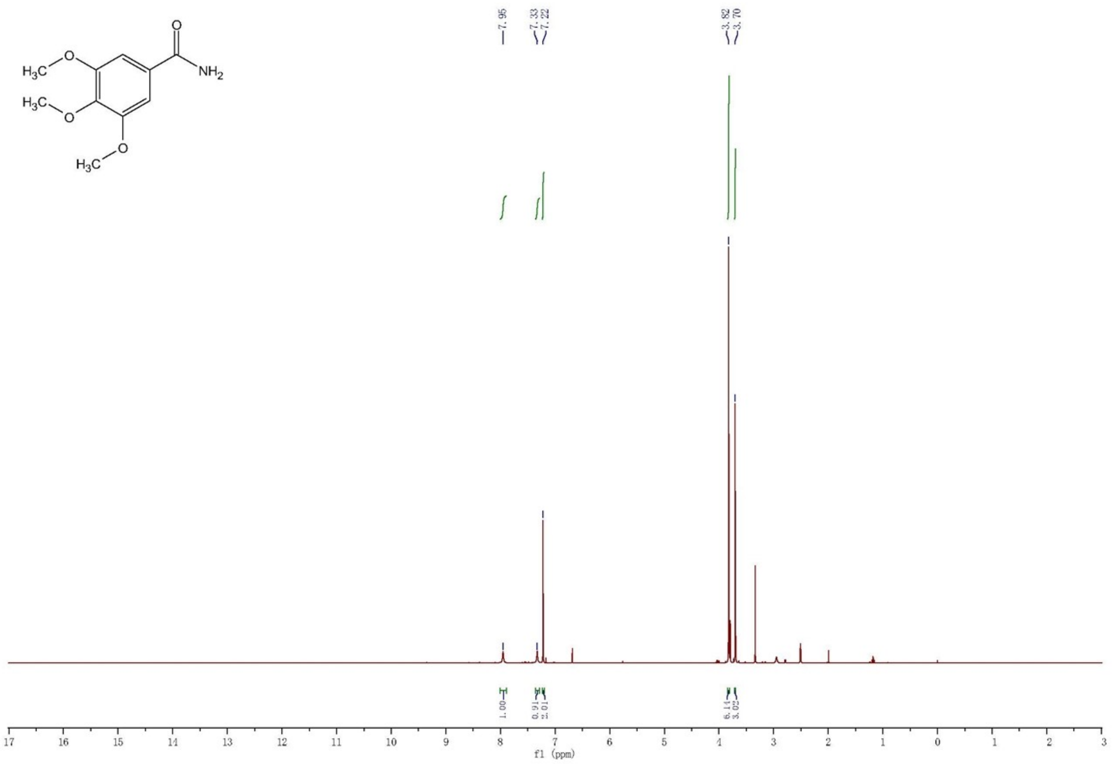

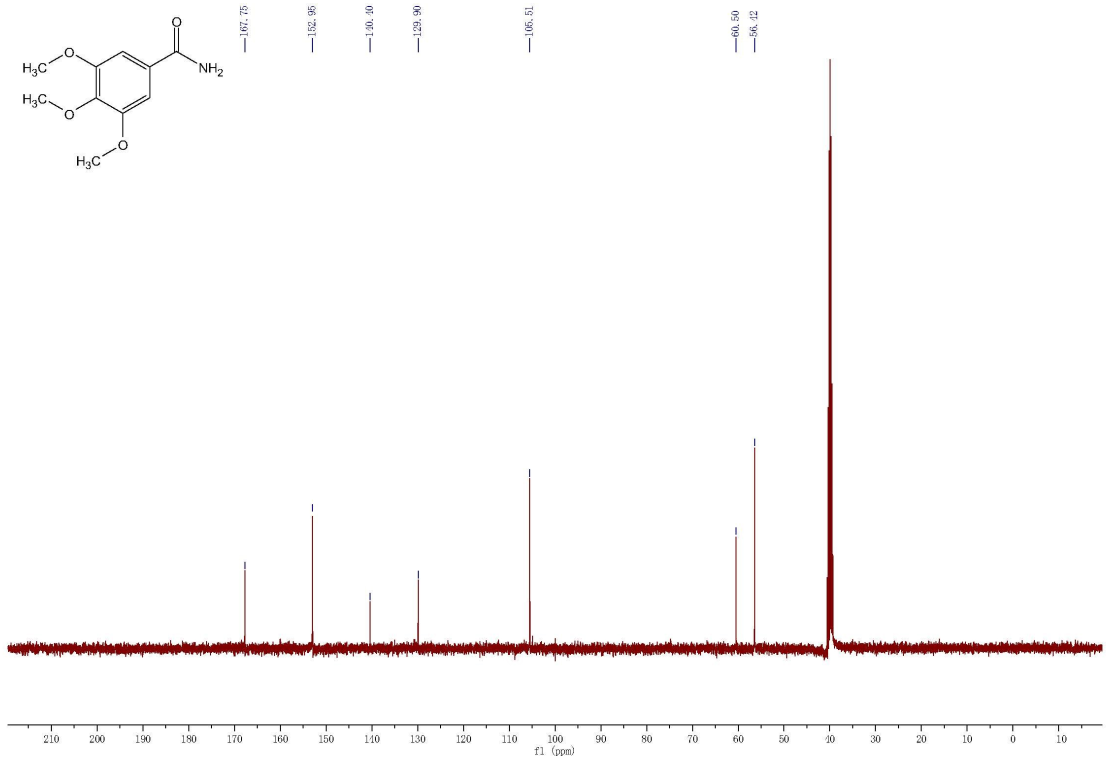

2w: thiophene-3-carboxamide

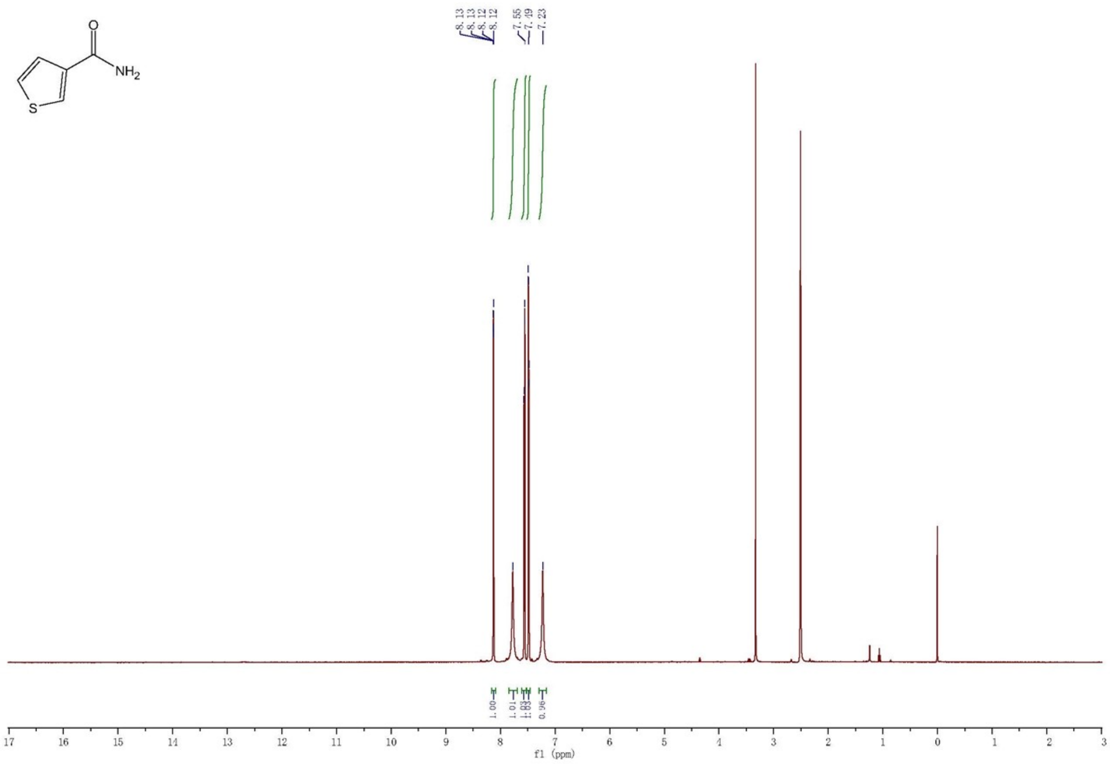

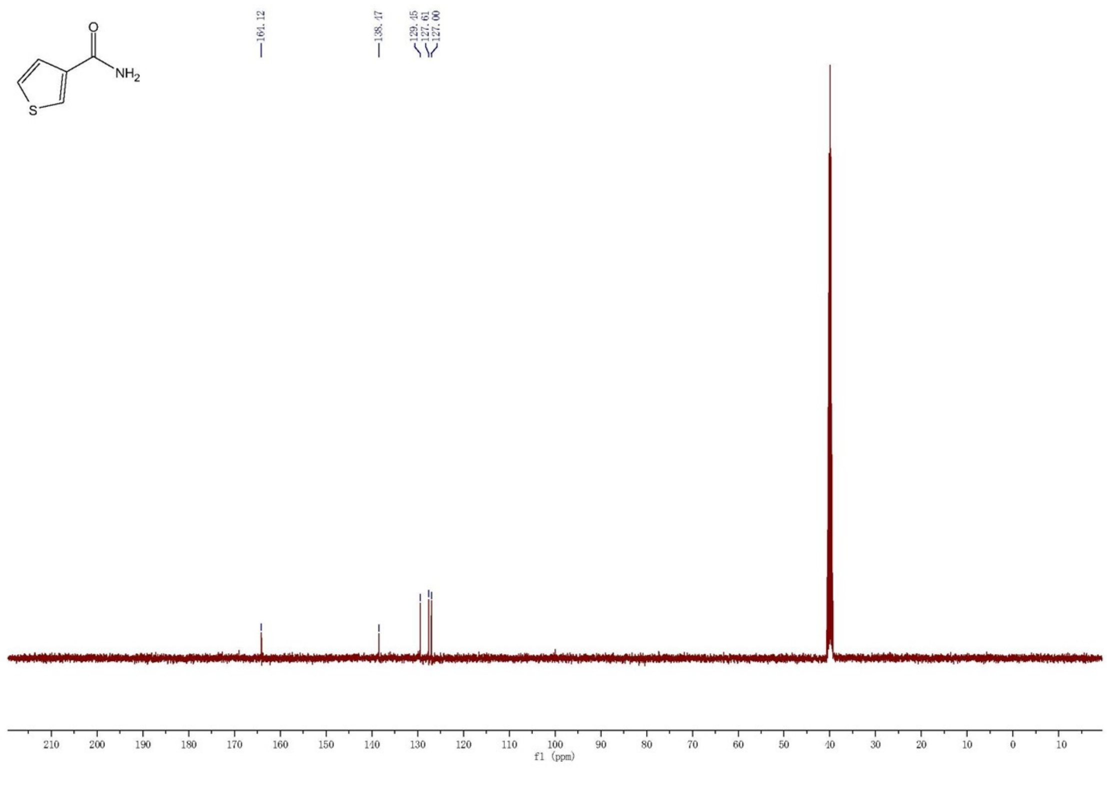

2x: nicotinamide

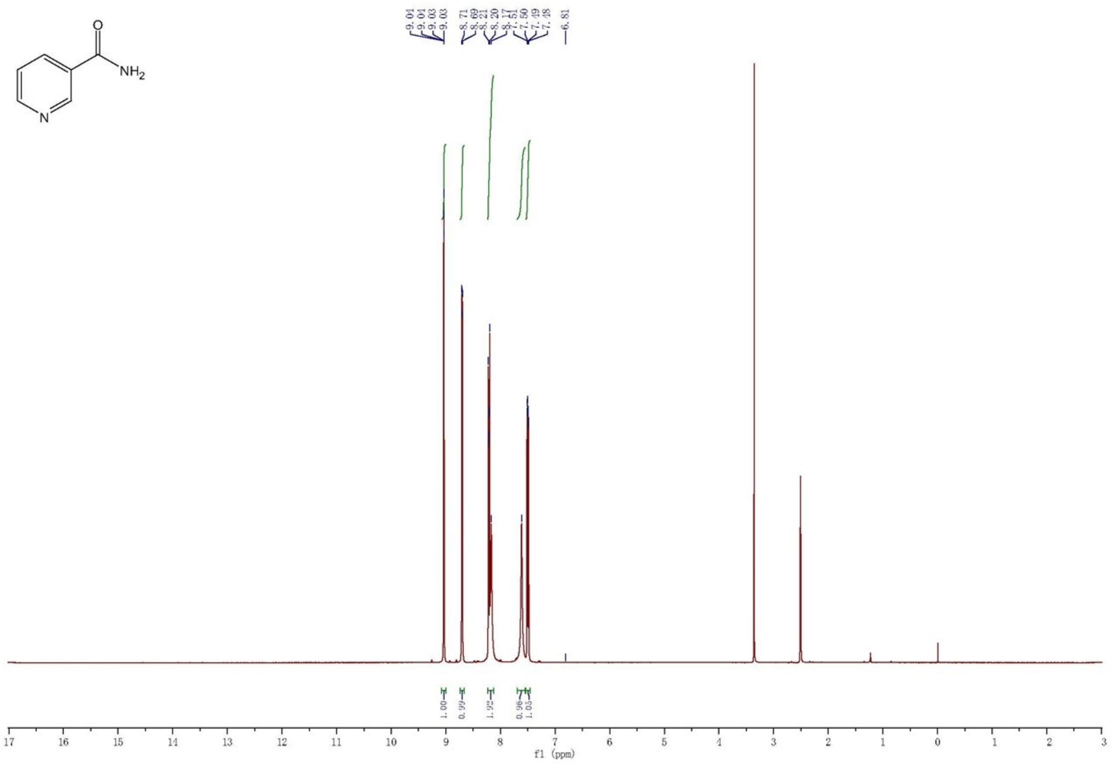

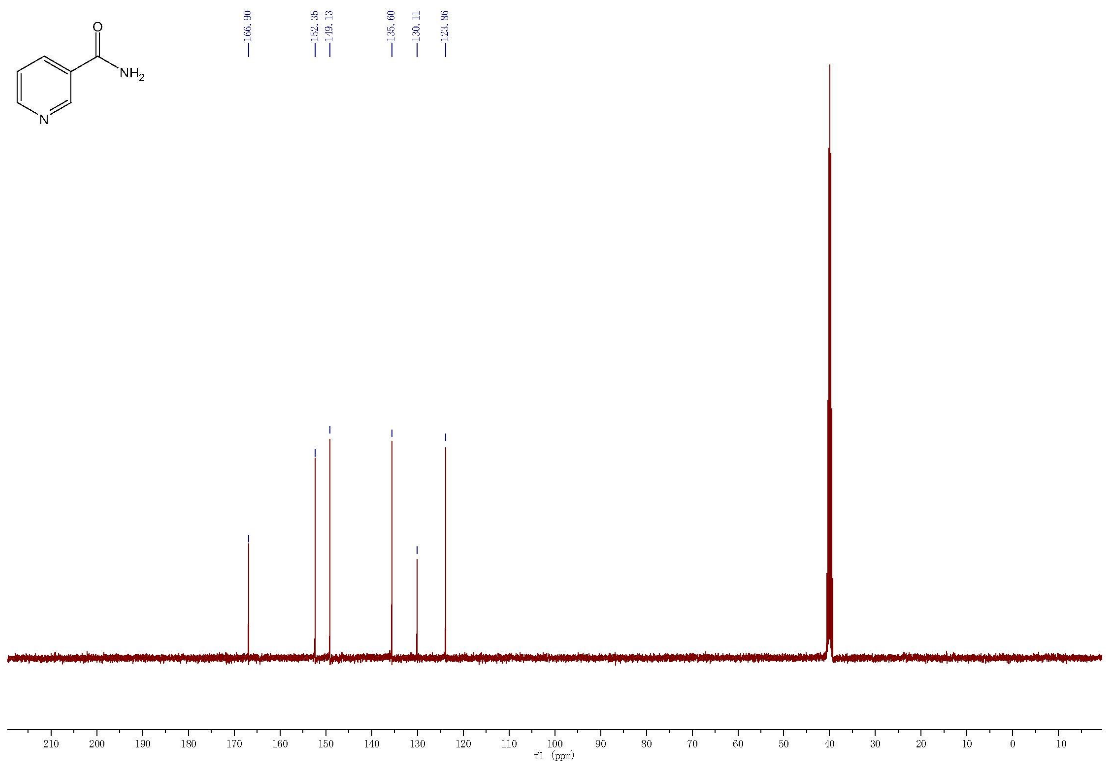

2z: 2-naphthamide

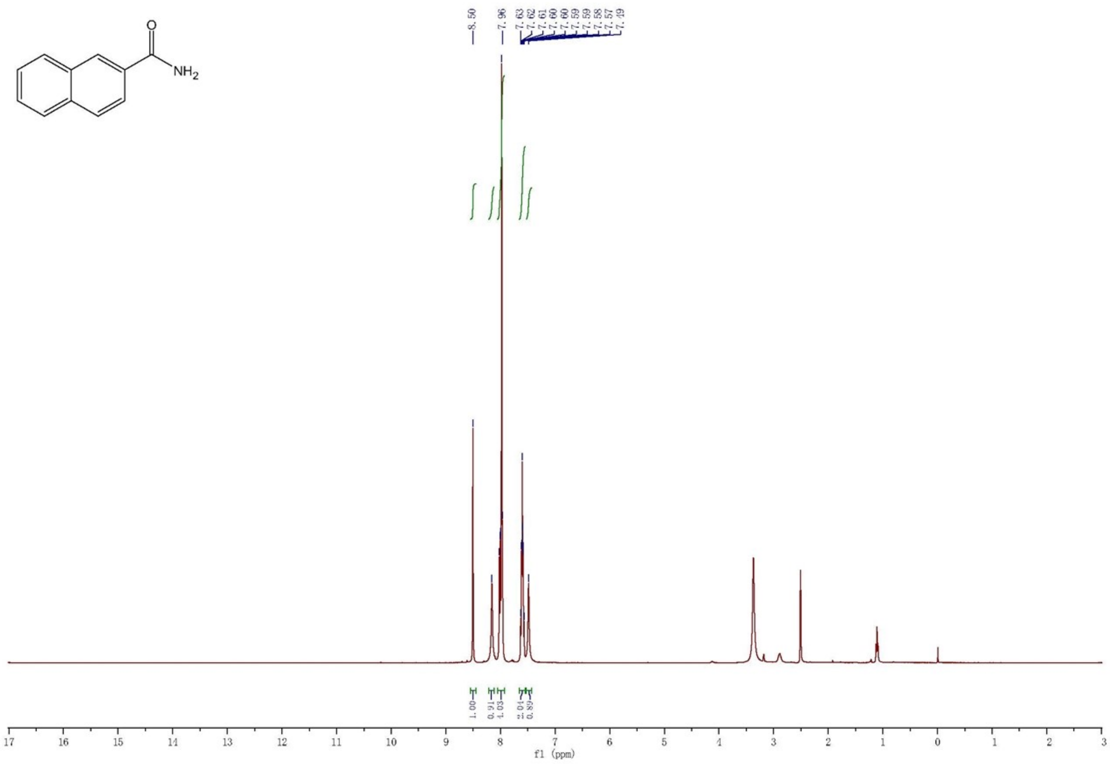

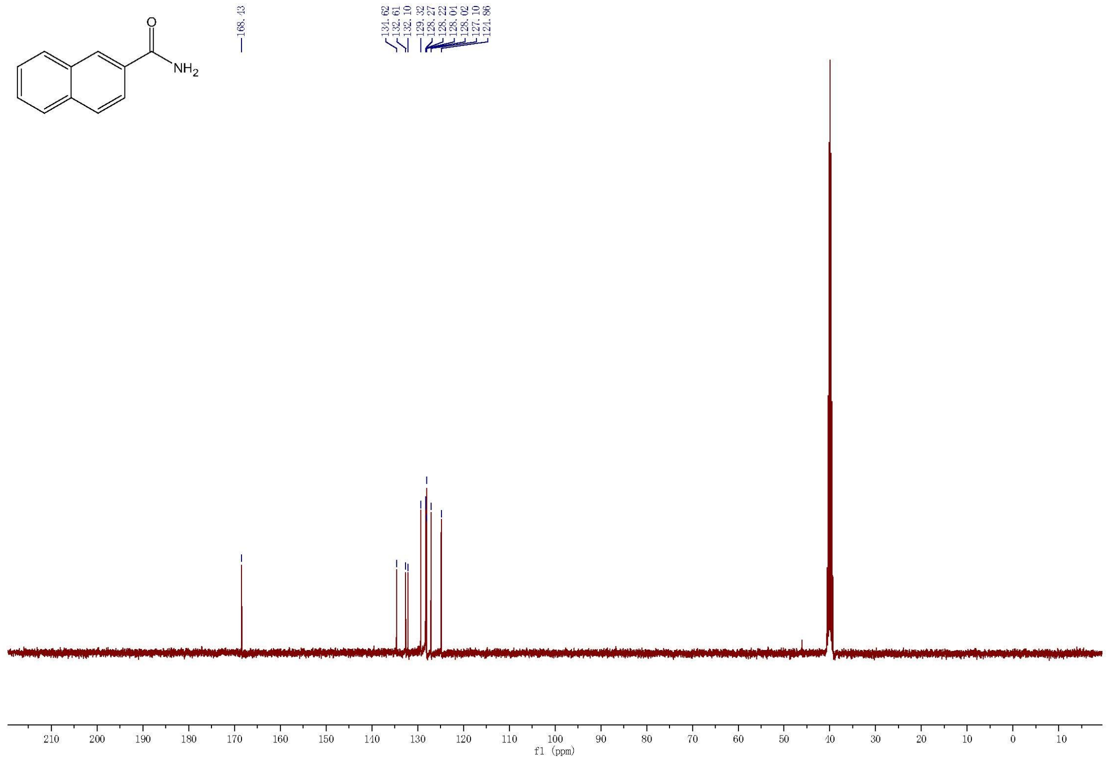

2aa: cyclopropanecarboxamide

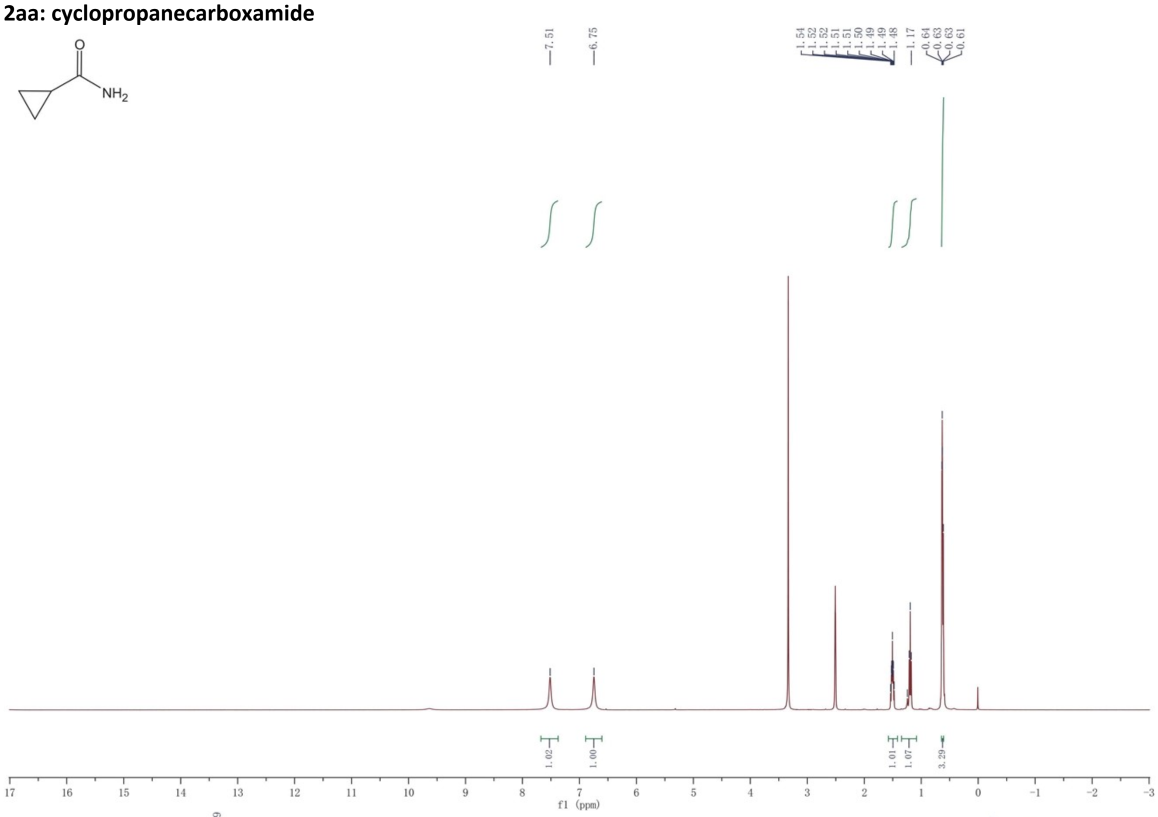

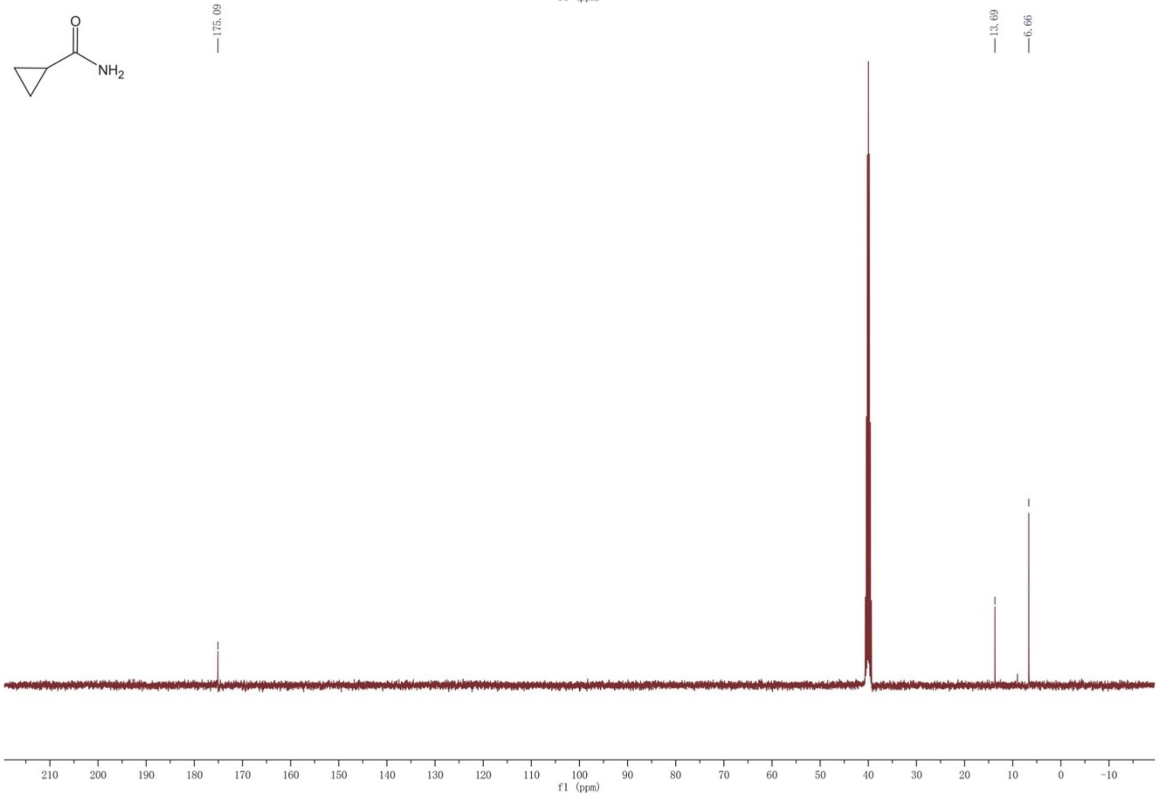

2bb: 2,2,3,3-tetramethylcyclopropanecarboxamide

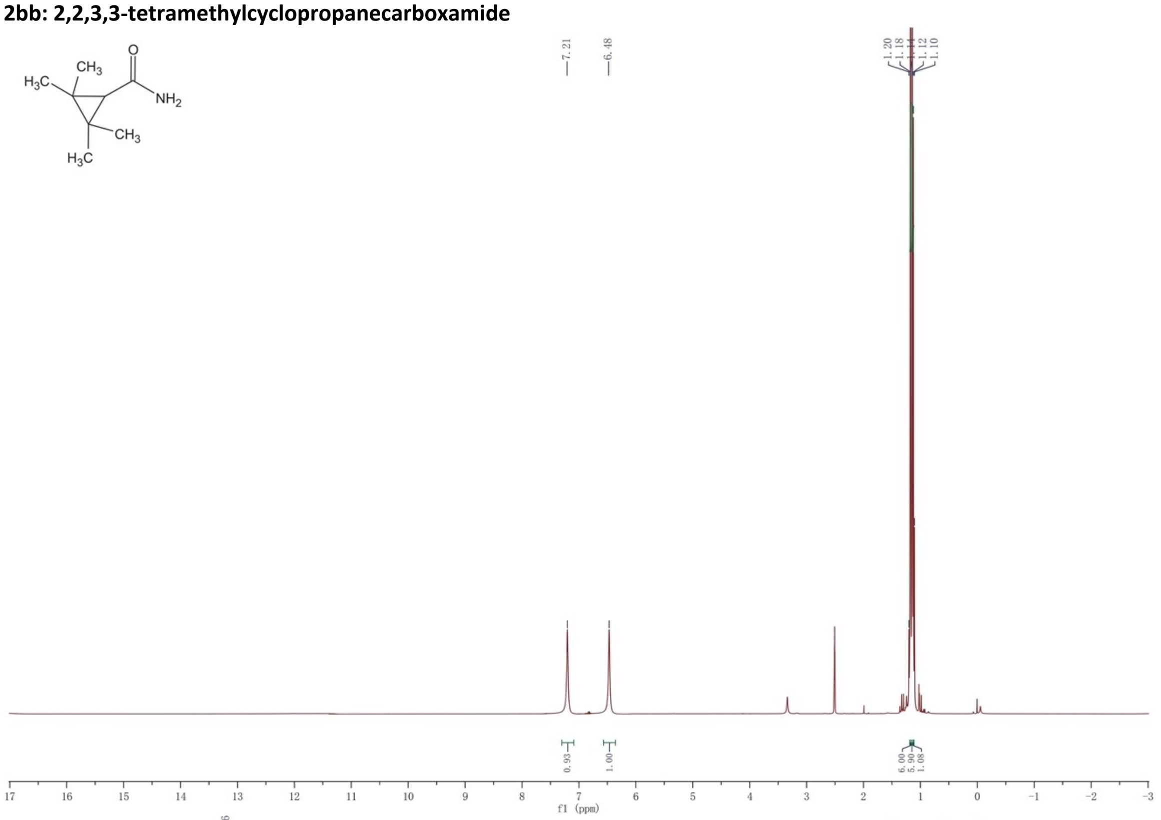

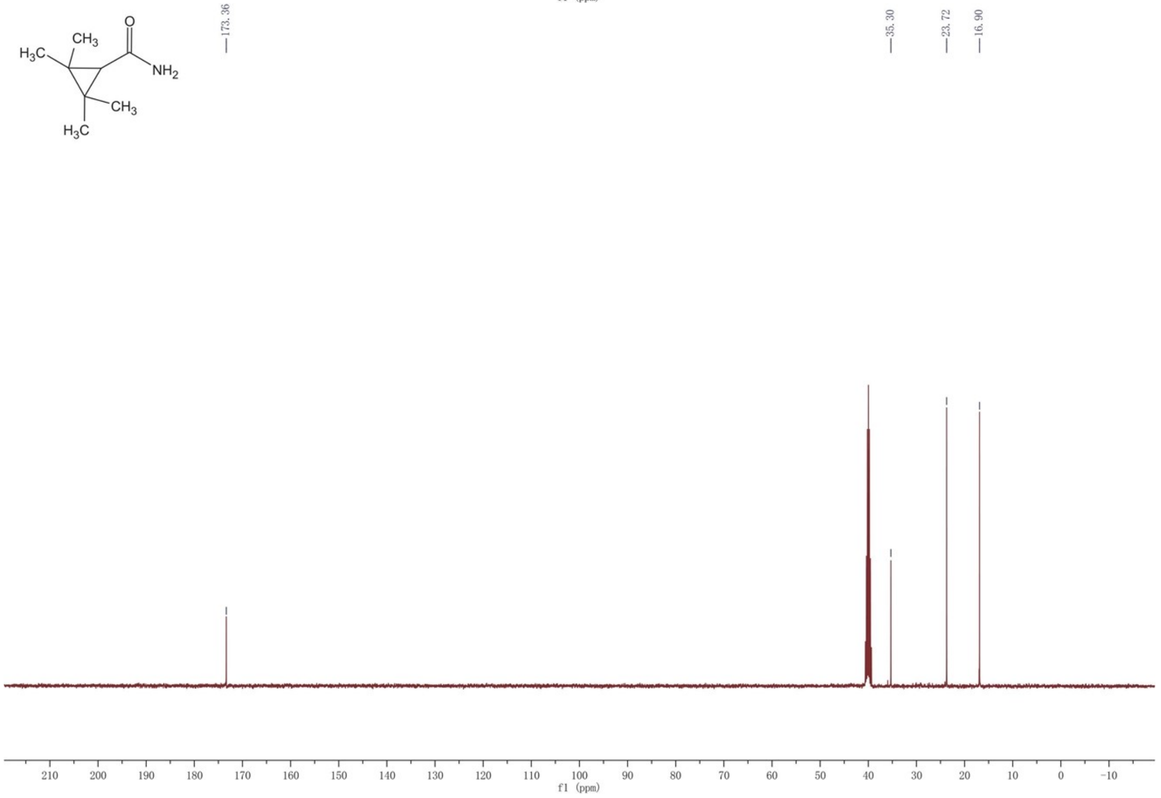

2cc: 3,3-dimethylbutanamide

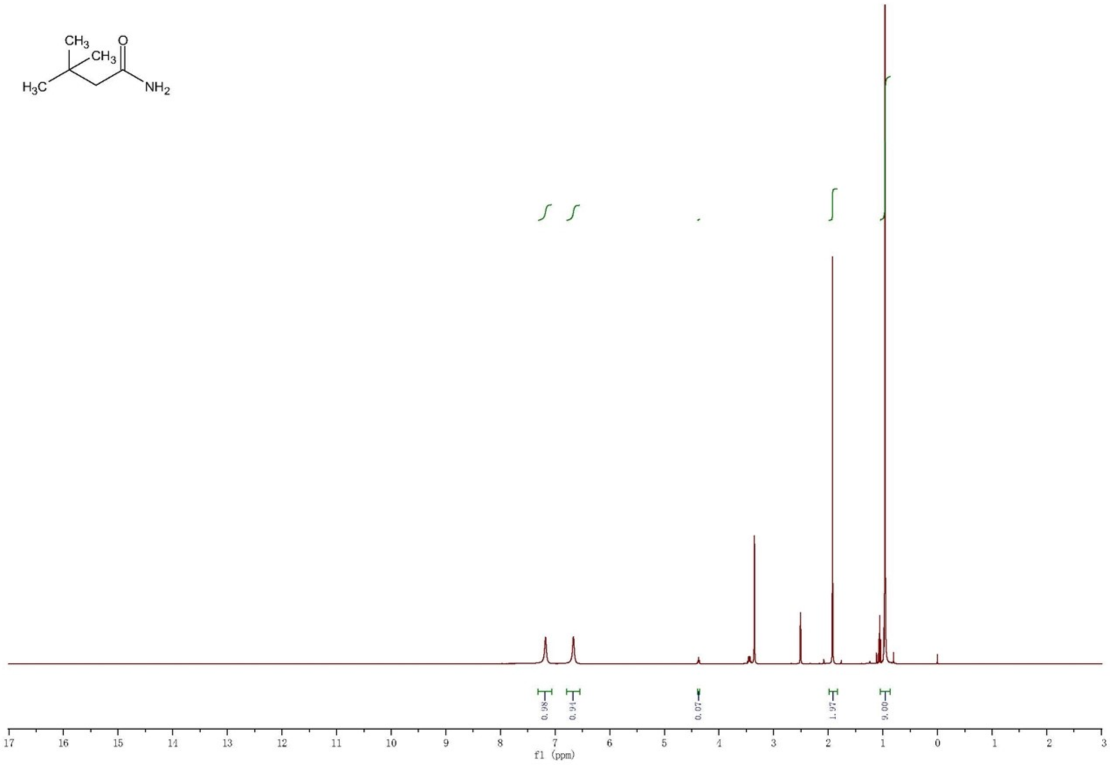

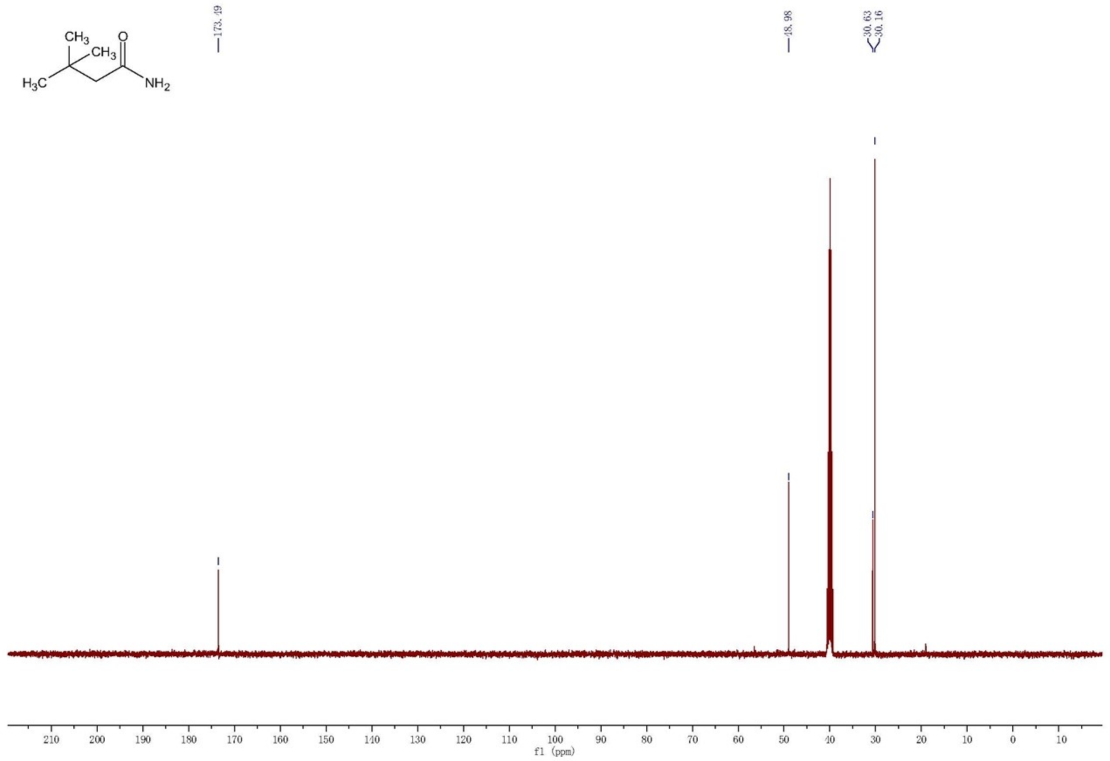

2dd: 5-methylhexanamide

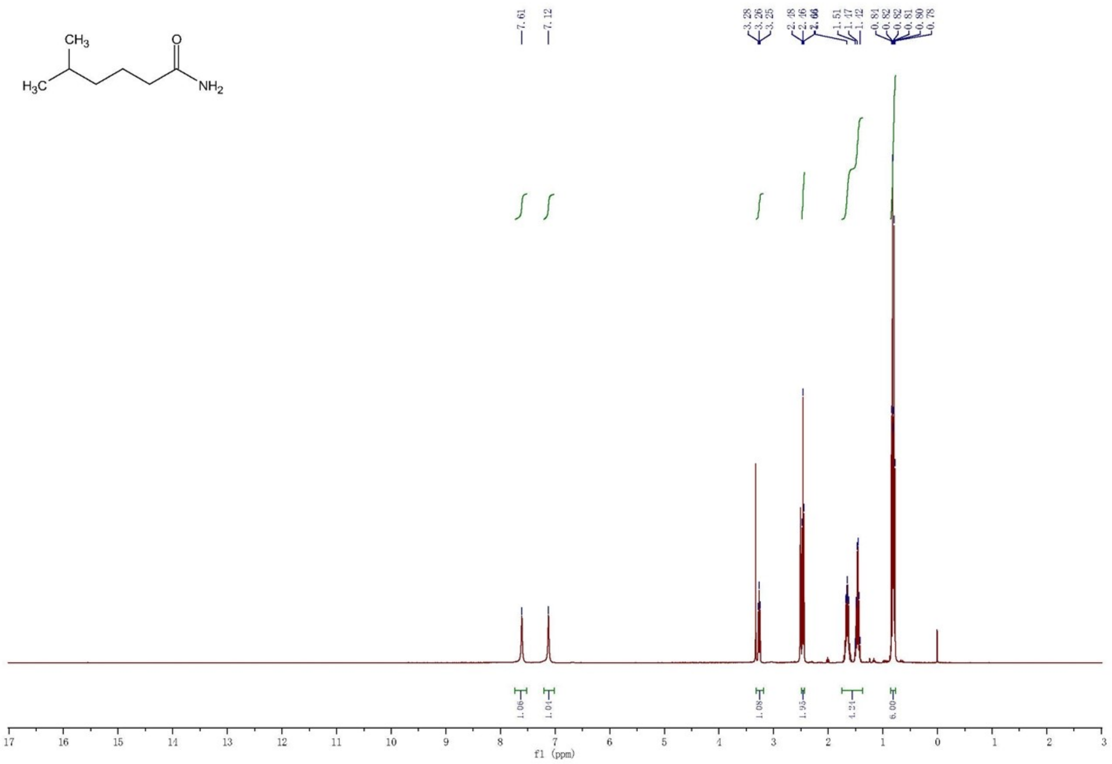

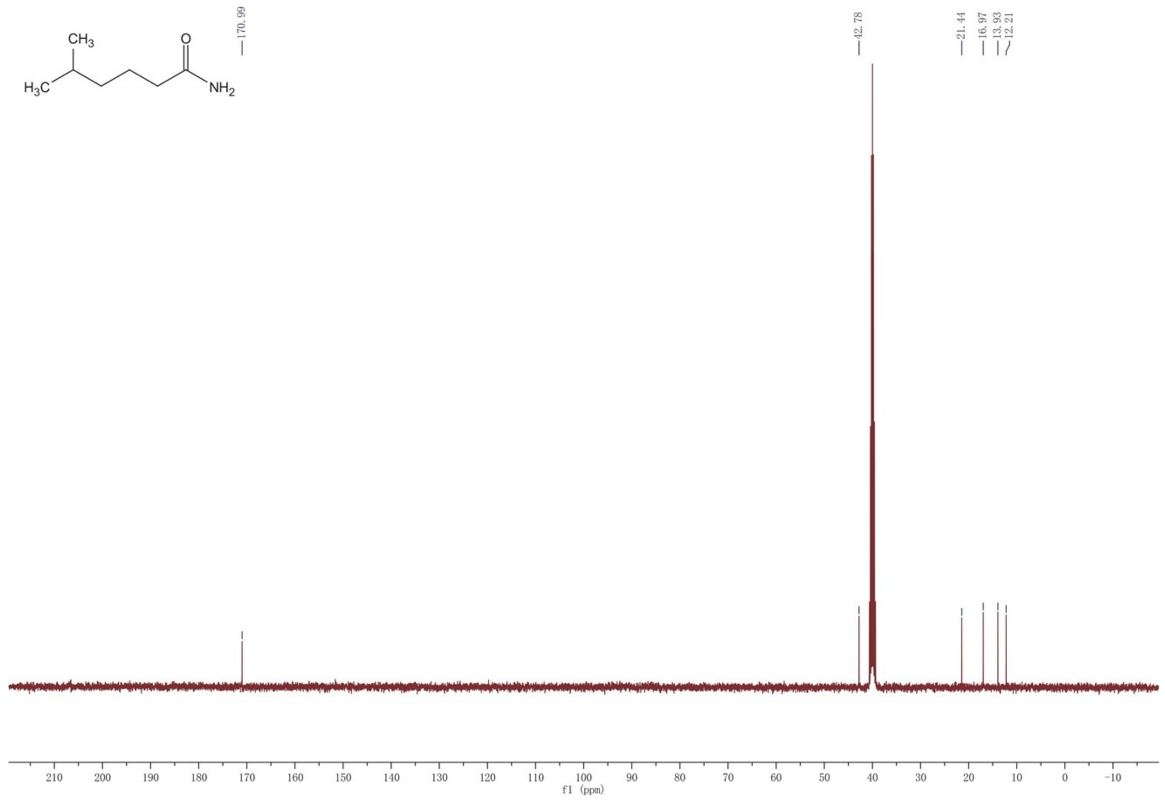

2ee: 6,7-dimethyloctanamide

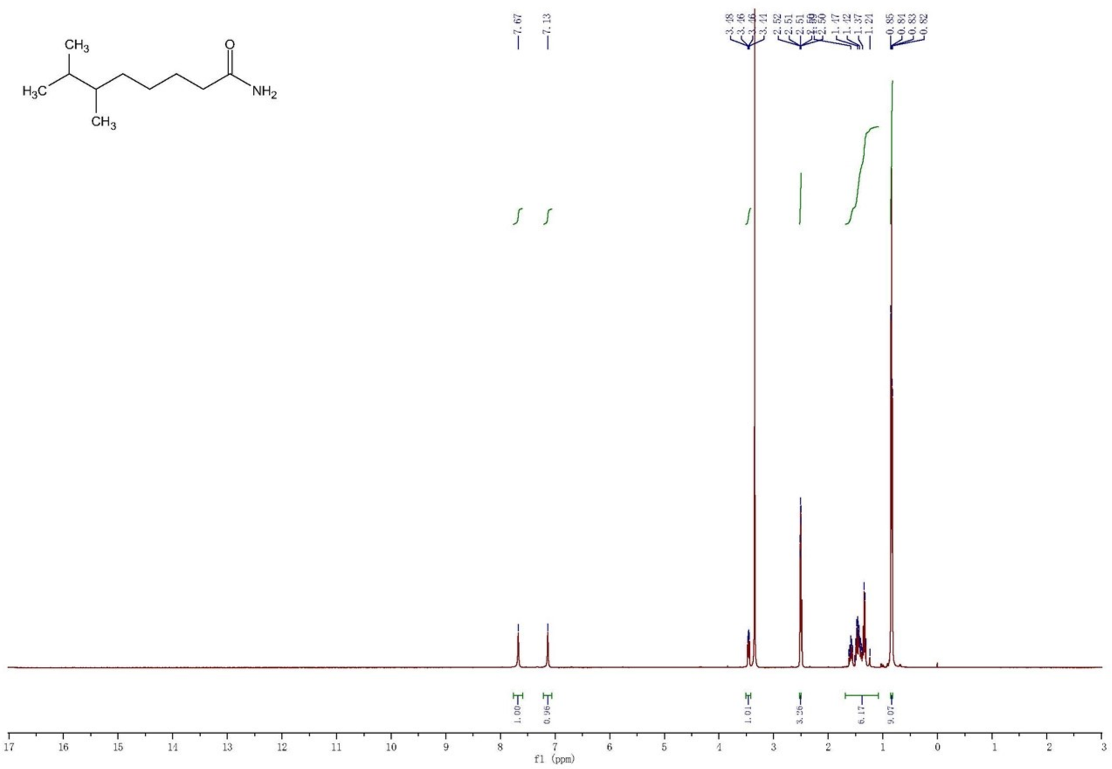

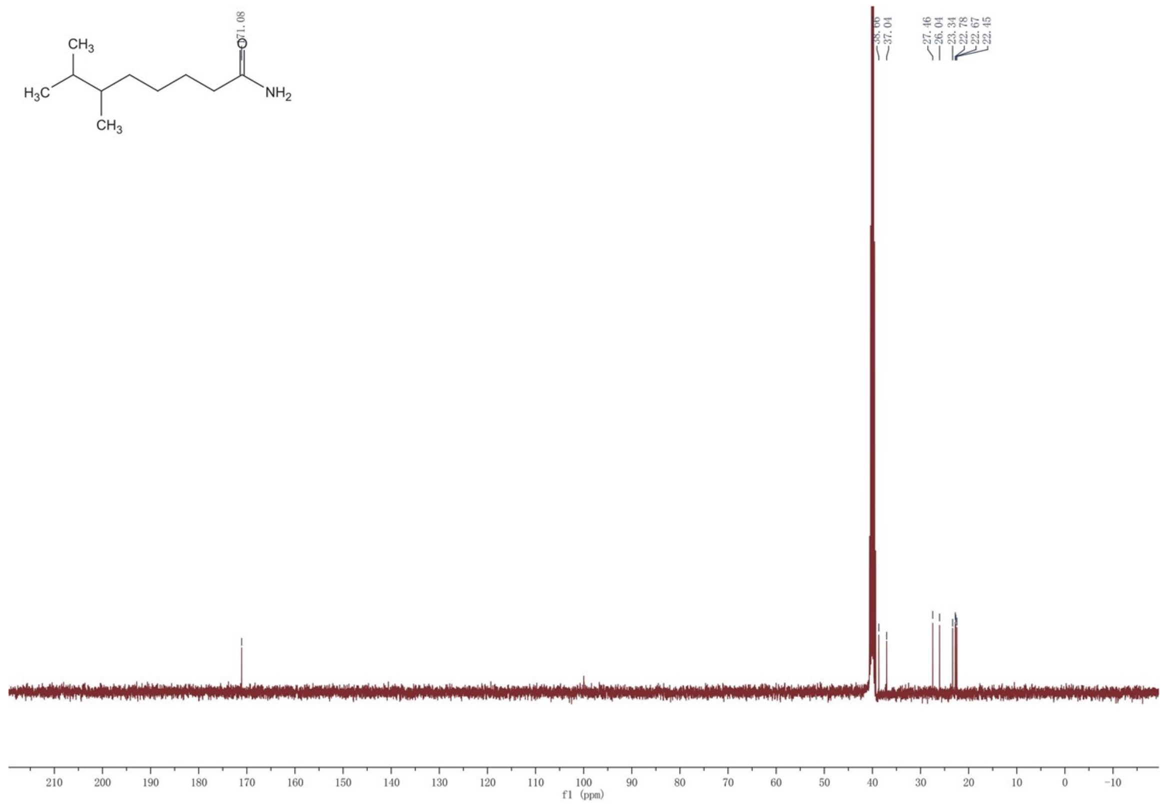

2ff: 2-phenylacetamide

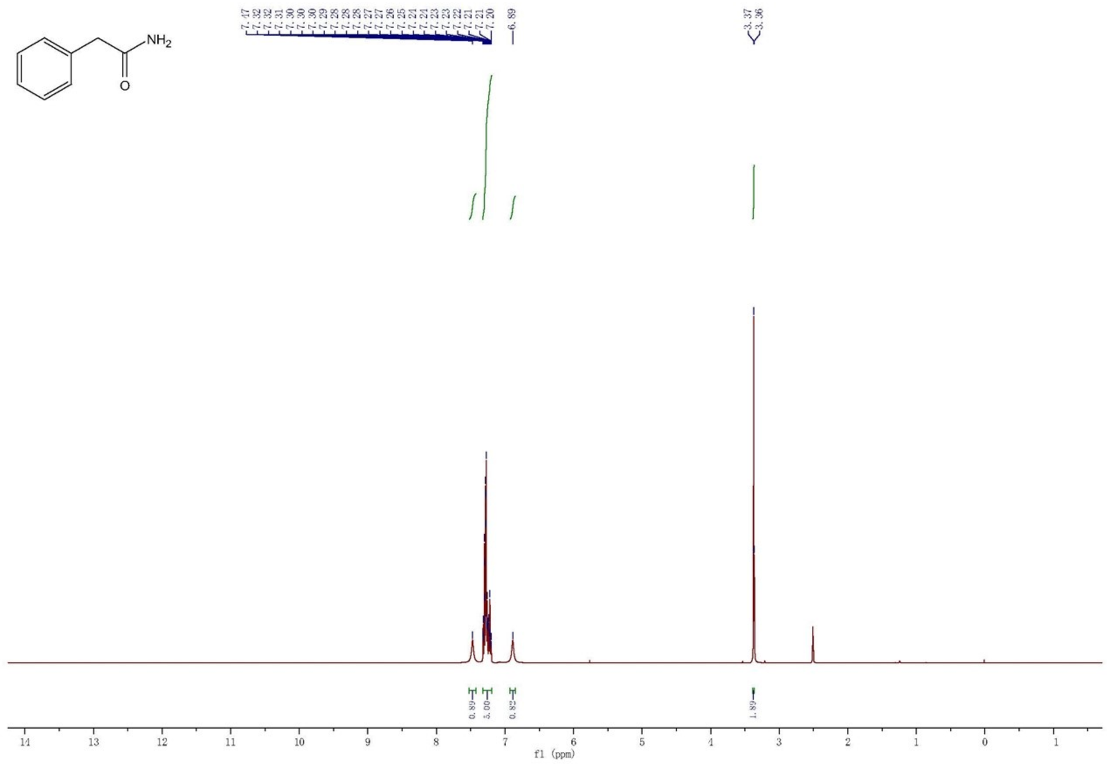

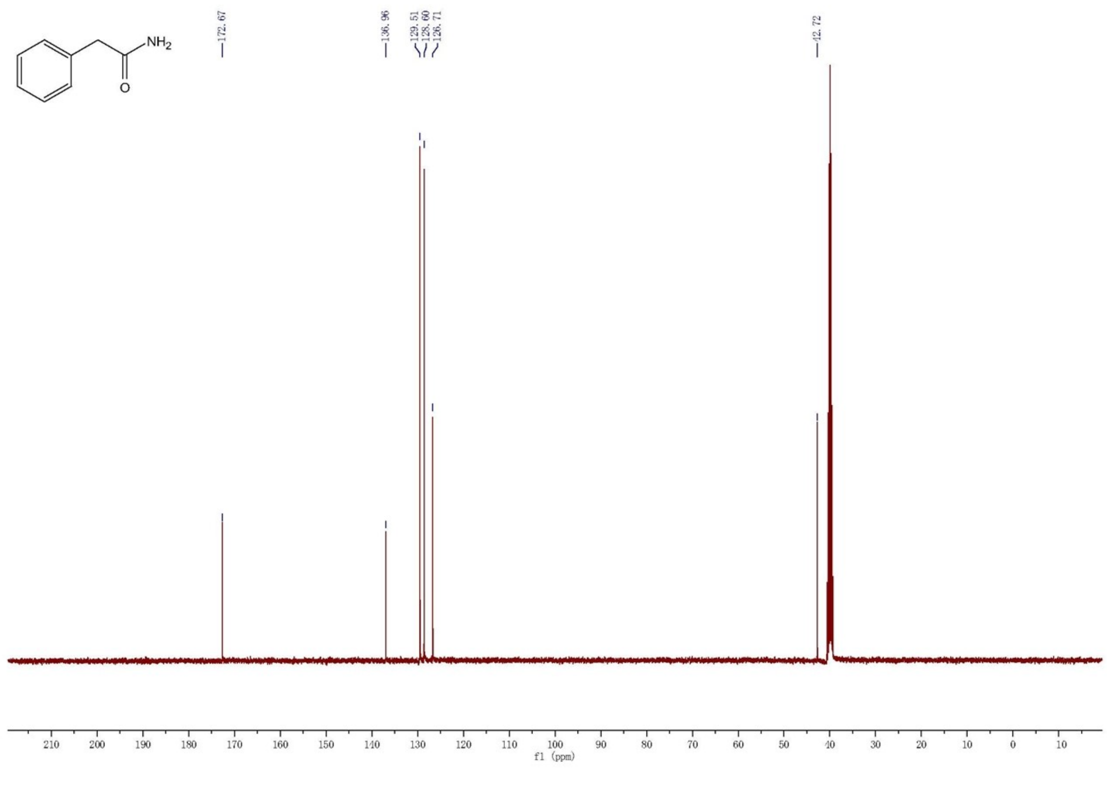

2gg: 4-phenylbutanamide

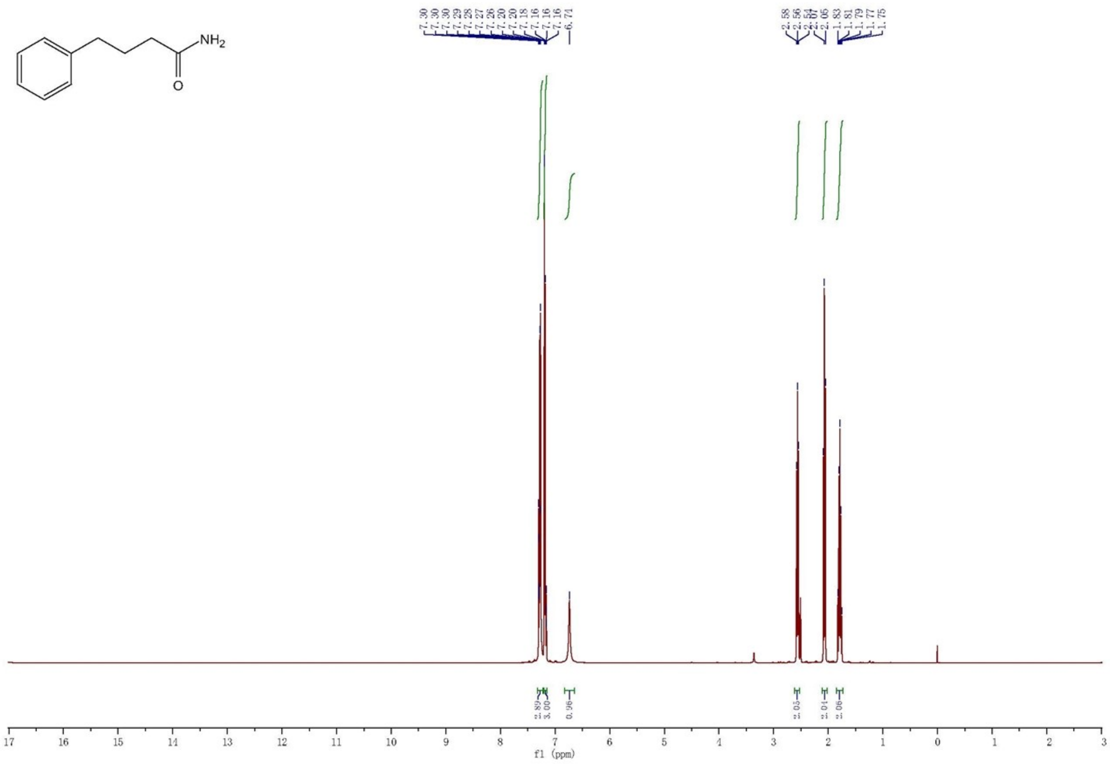

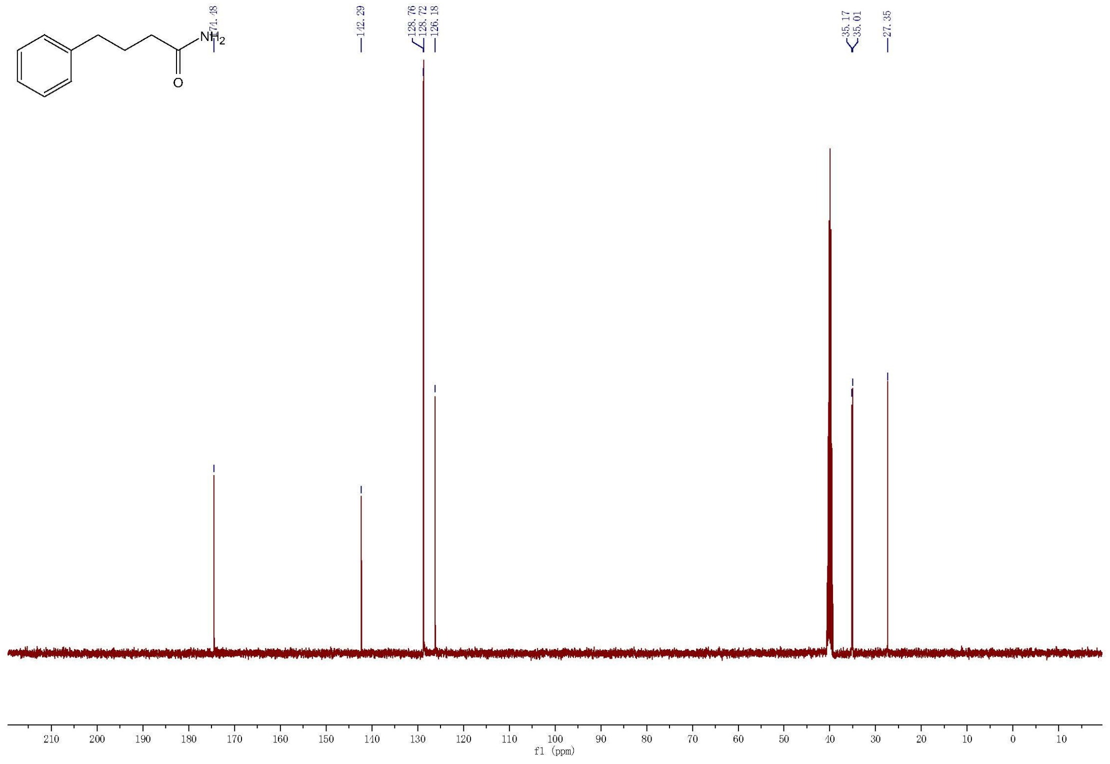

2hh: 2-(naphthalen-1-yl)acetamide

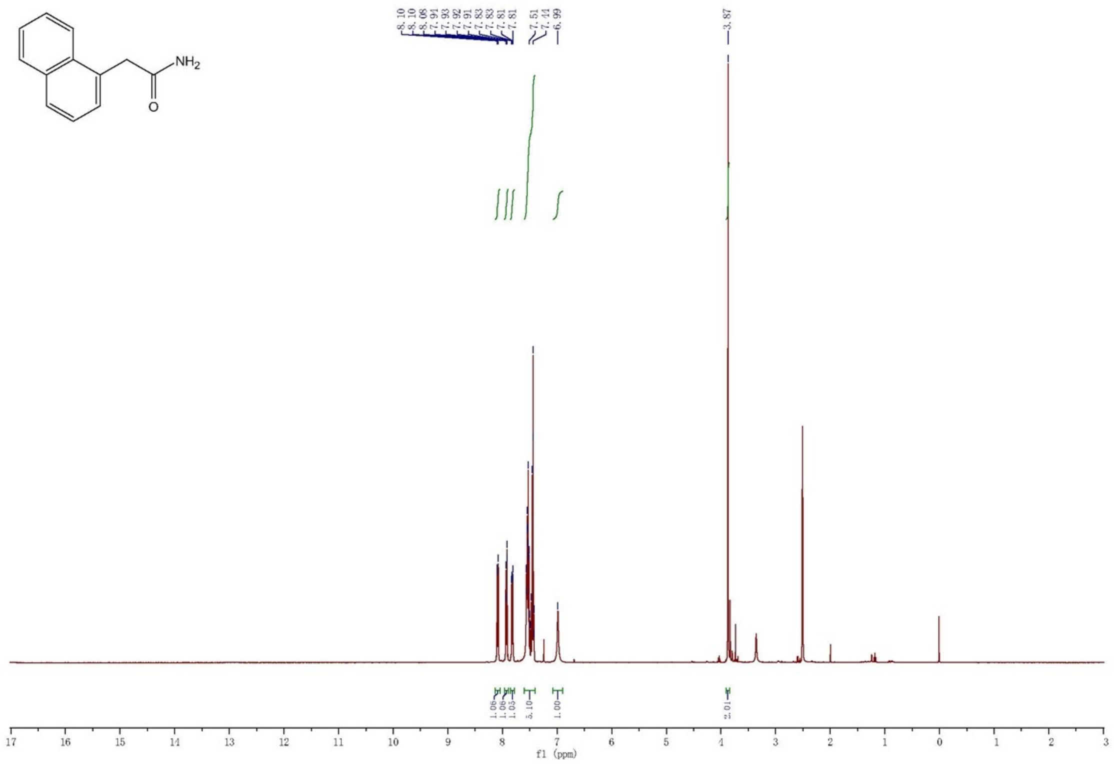

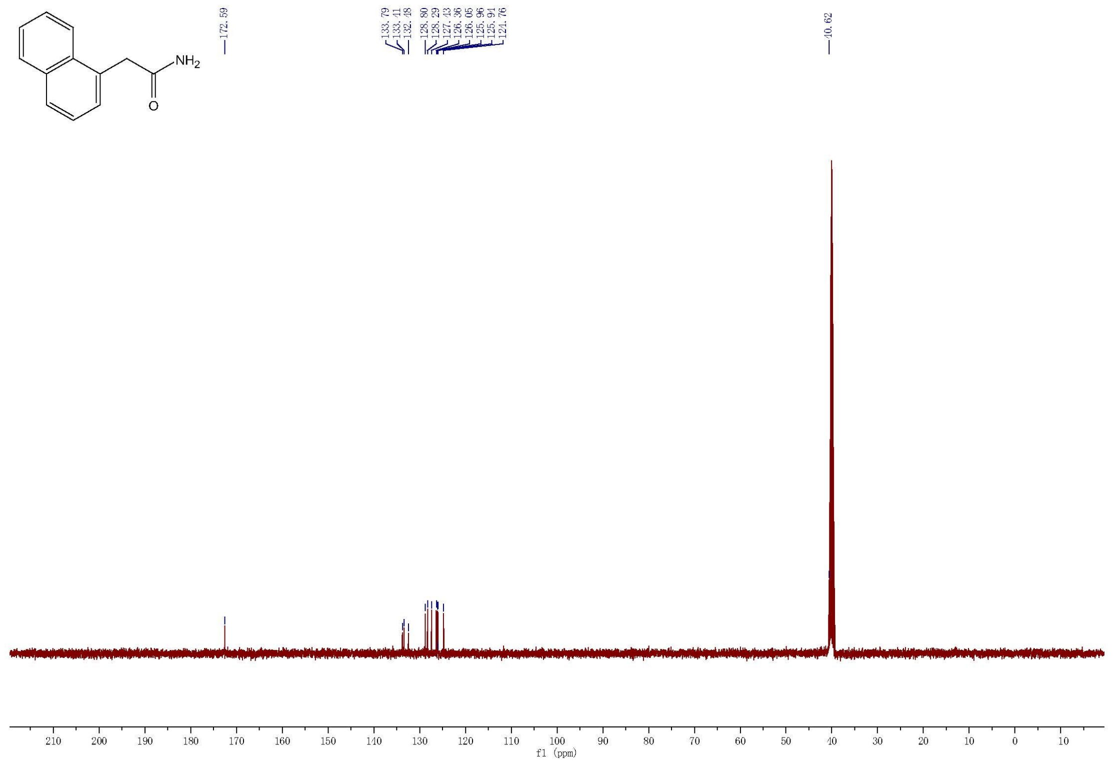

2ii: adamantane-1-carboxamide

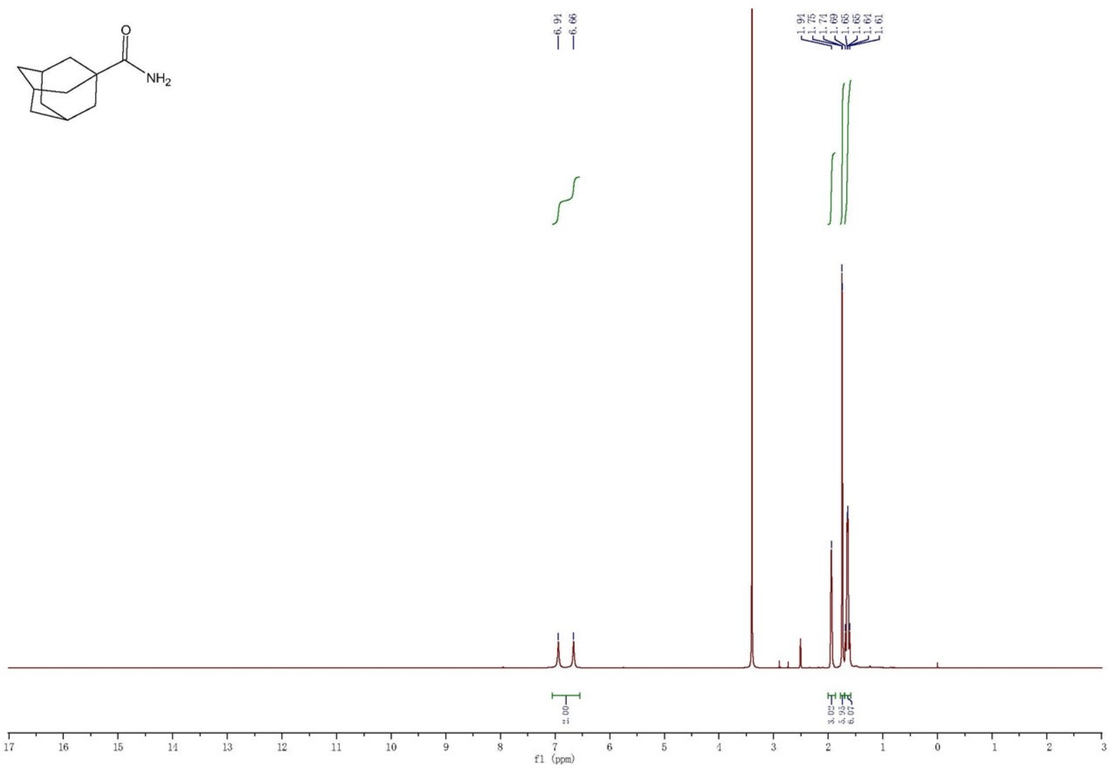

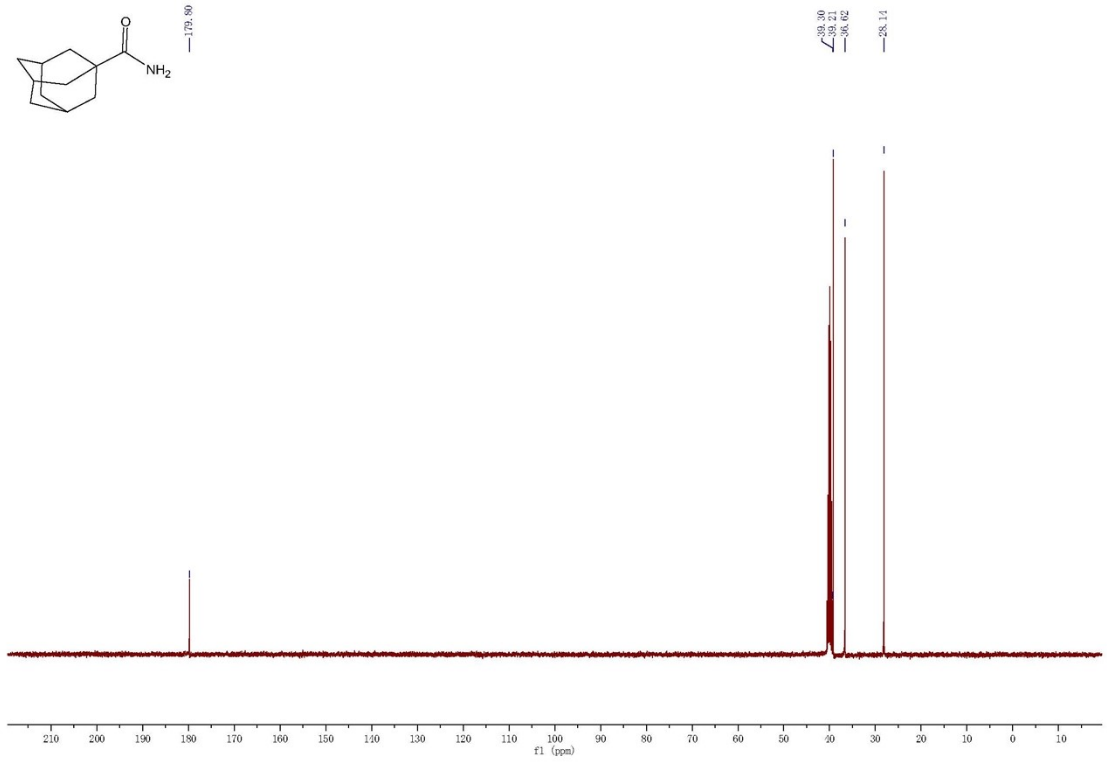

2y: 2-(2-oxopyrrolidin-1-yl)acetamide

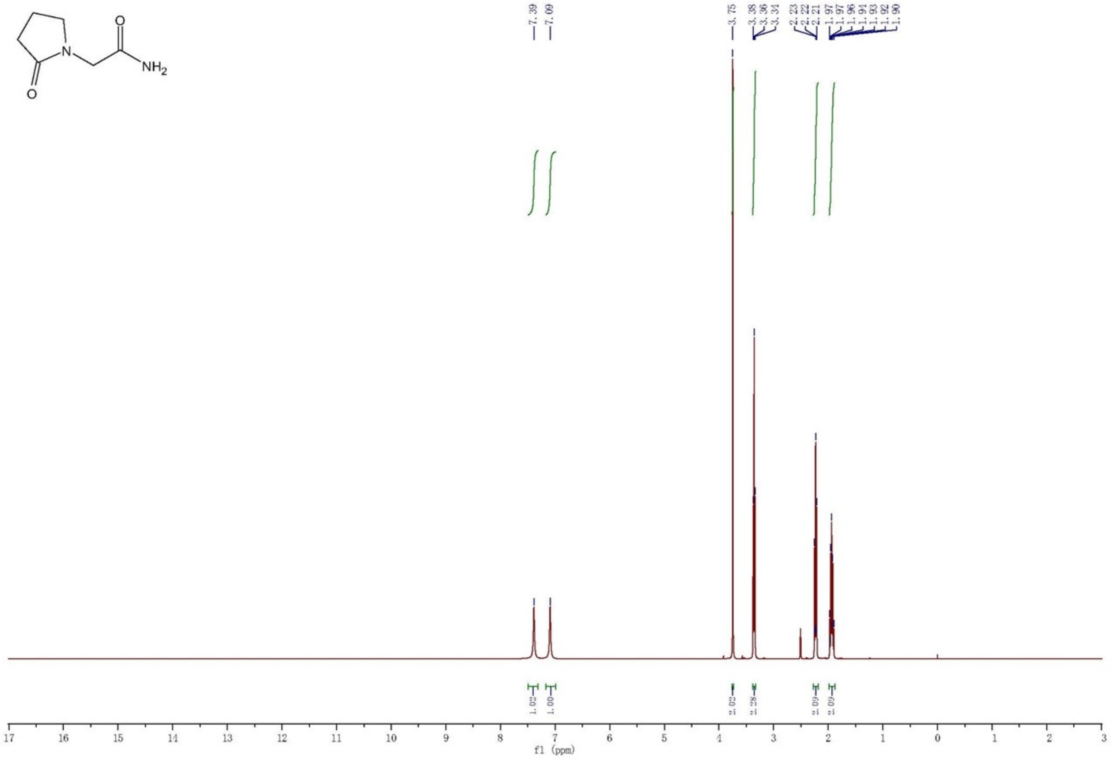

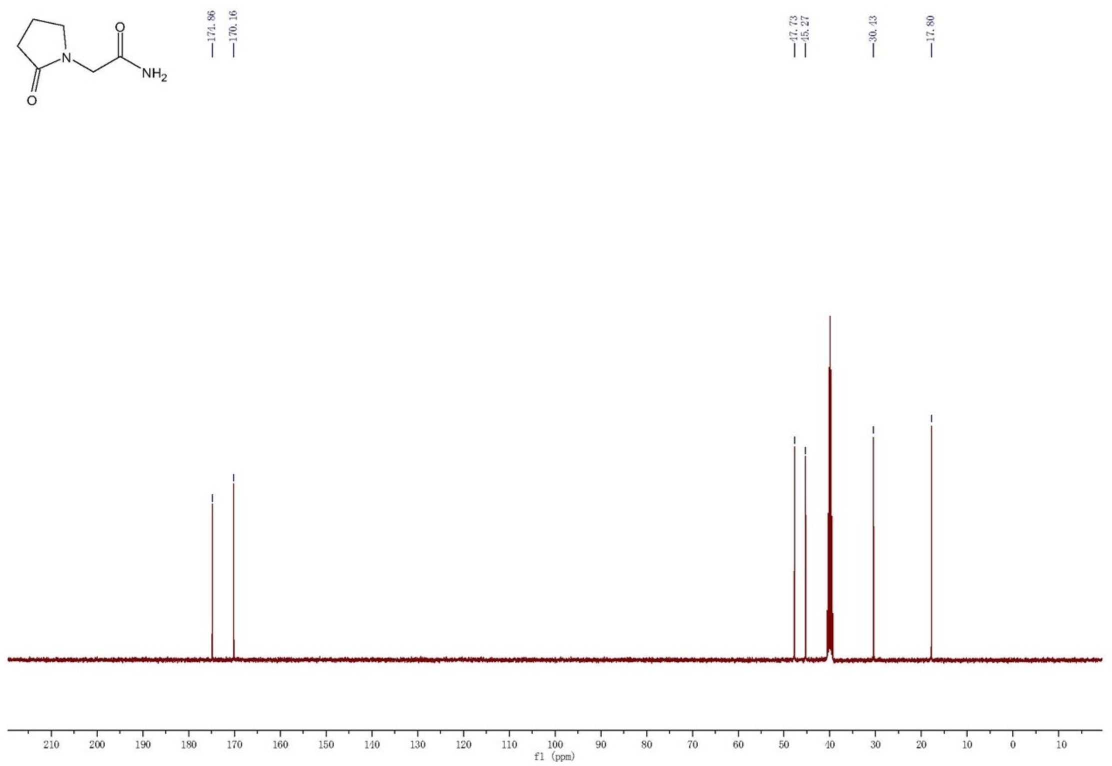

2jj: (R)-pyrrolidine-2-carboxamide

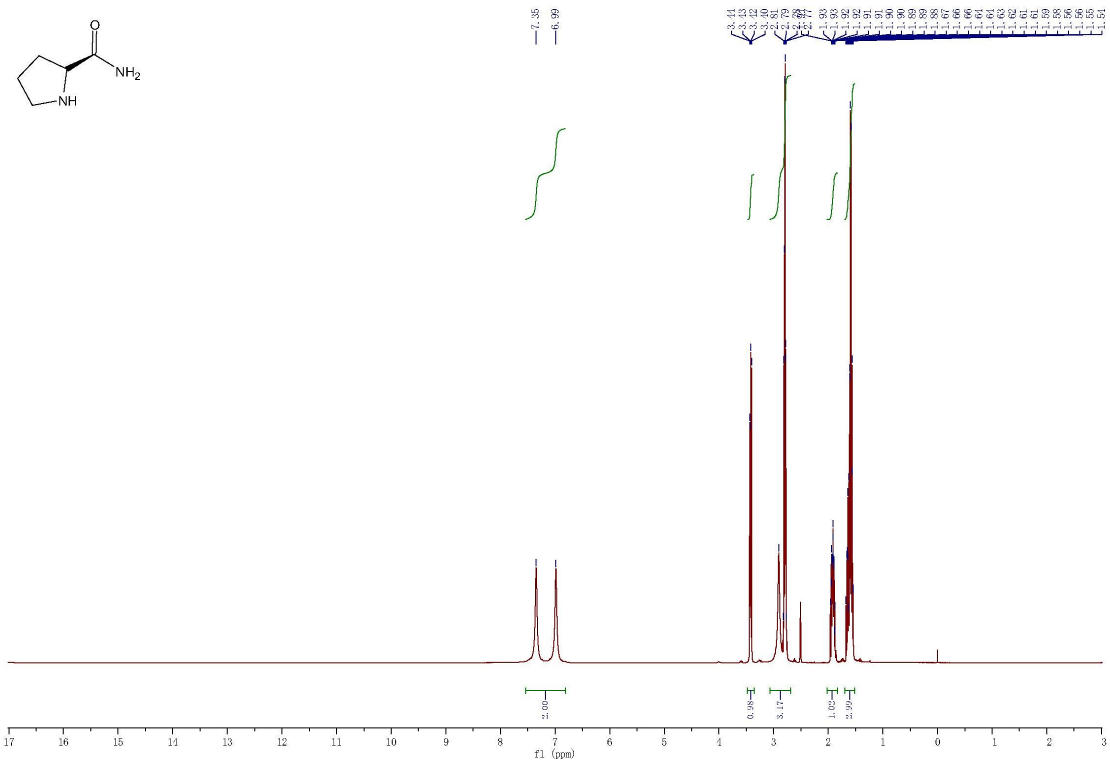

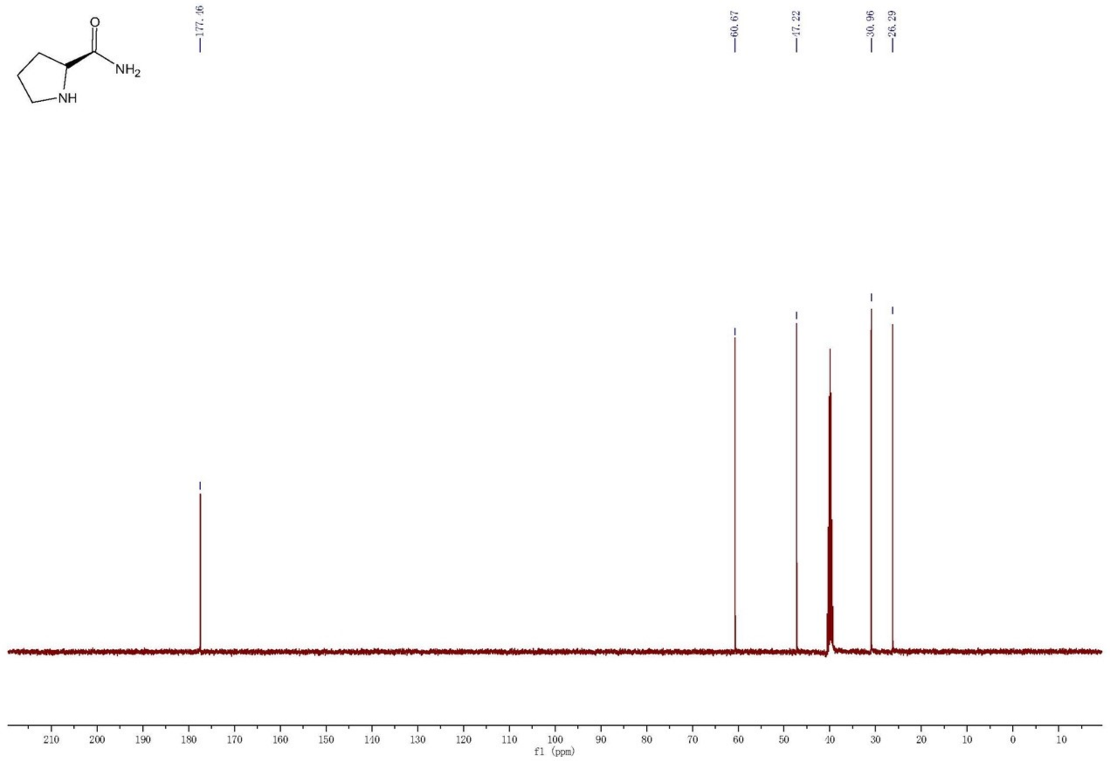

2v: 4-ethylbenzamide

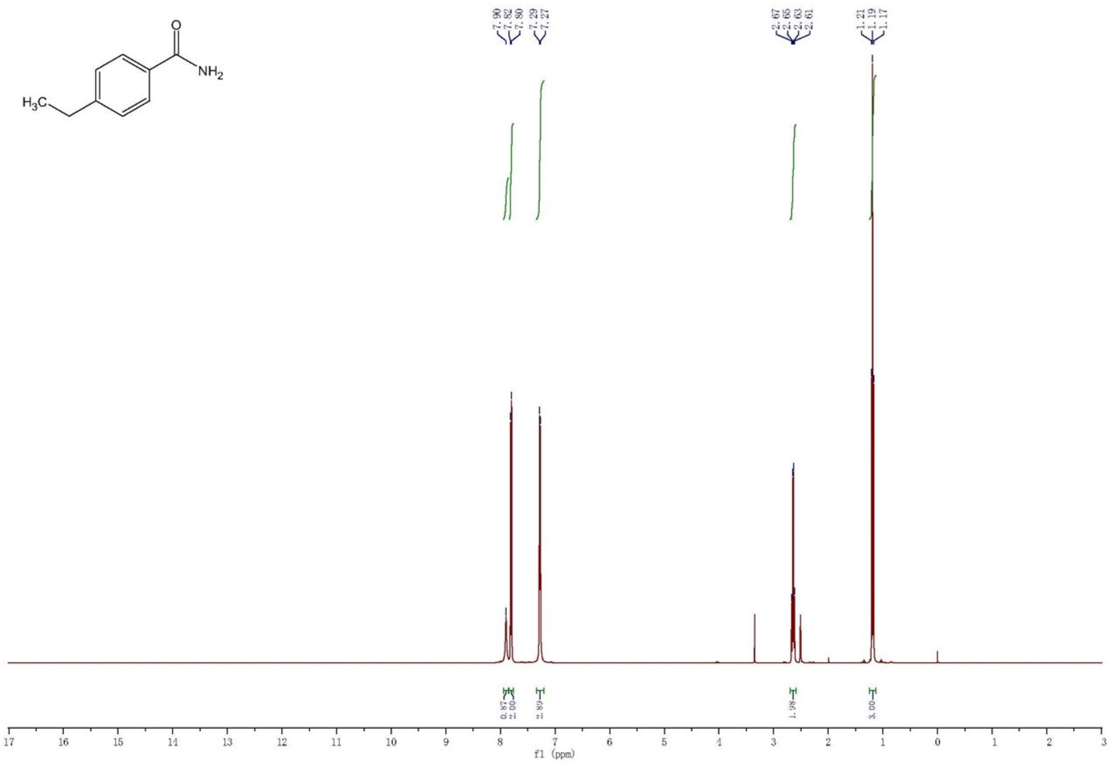

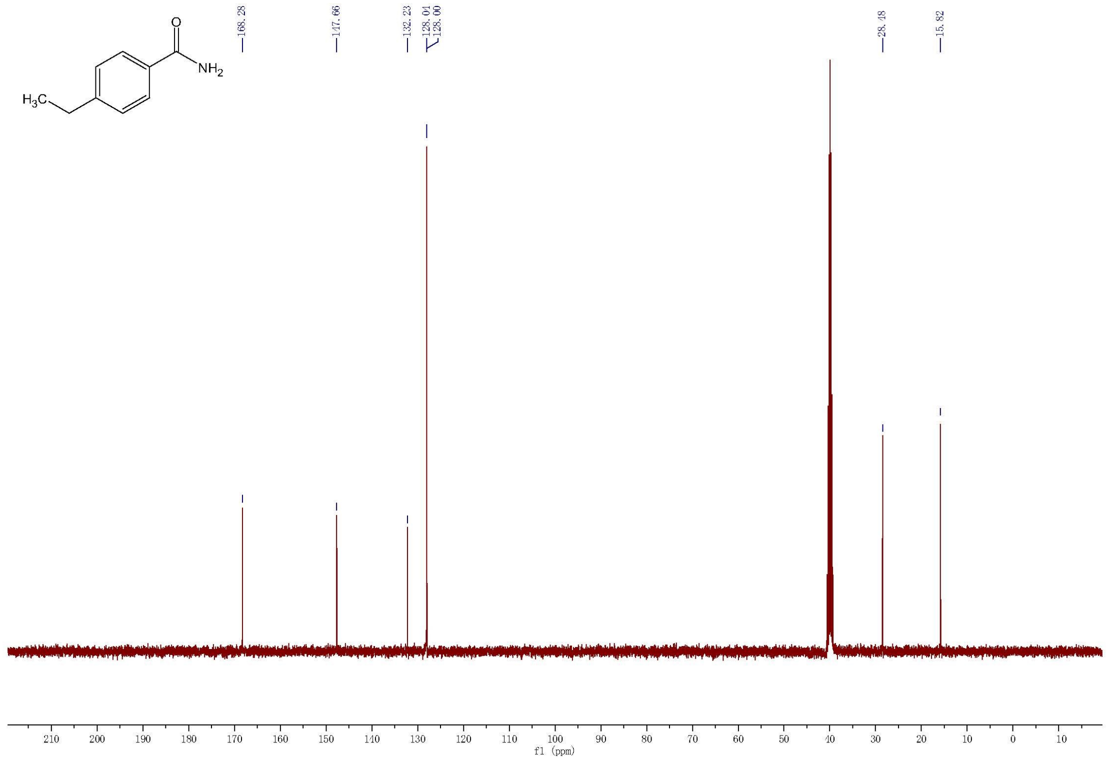

2kk: 4’-((1,7’-dimethyl-2’-propyl-1H,3’H-[2,5’-bibenzo[d]imidazol]-3’-yl)methyl)-[1,1’-biphenyl]-2-carboxamide

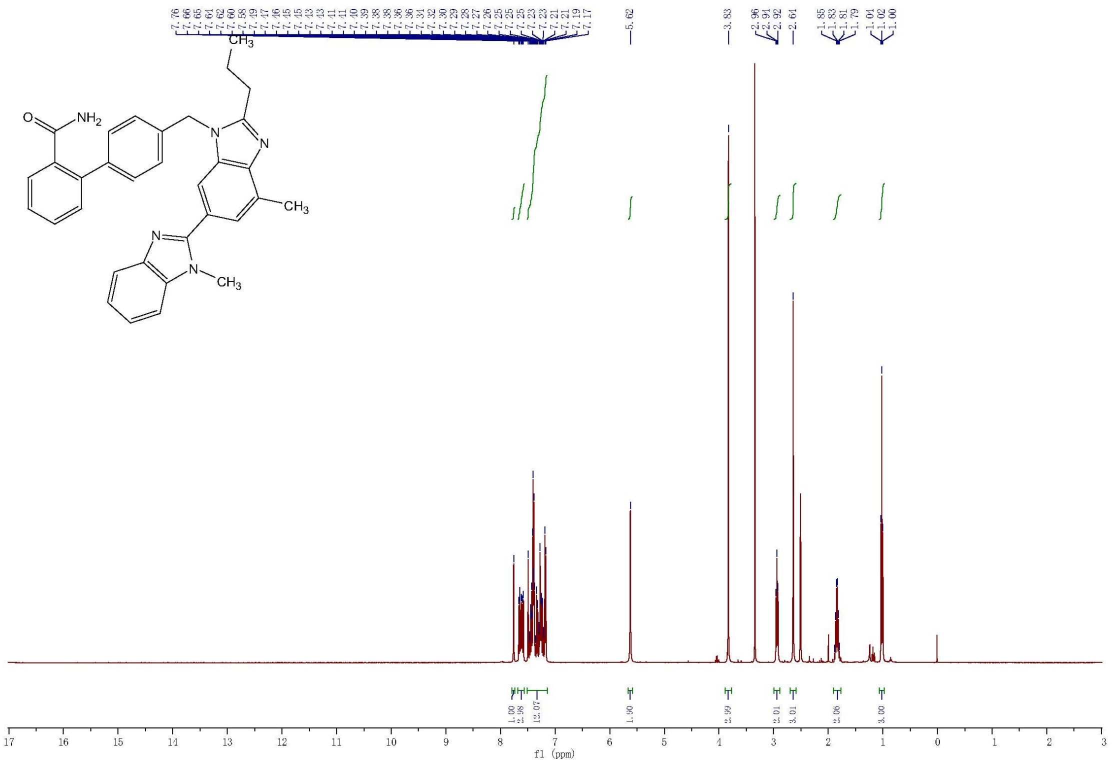

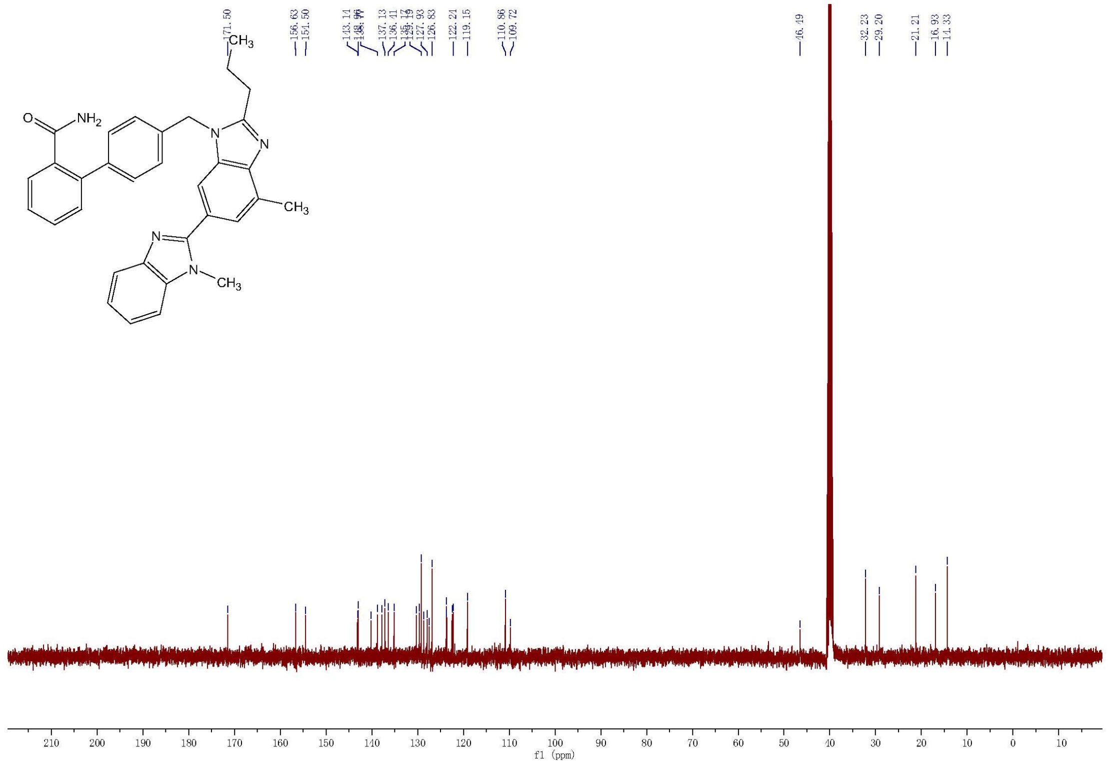

Supplement: RA-012-D2RA04709H-s001 [file RA-012-D2RA04709H-s001.pdf]
